# ASaiM: a Galaxy-based framework to analyze microbiota data

**DOI:** 10.1093/gigascience/giy057

**Published:** 2018-05-15

**Authors:** Bérénice Batut, Kévin Gravouil, Clémence Defois, Saskia Hiltemann, Jean-François Brugère, Eric Peyretaillade, Pierre Peyret

**Affiliations:** 1 Université Clermont Auvergne, EA 4678 CIDAM, 63000 Clermont-Ferrand, France (previous address); 2 Bioinformatics Group, Department of Computer Science, University of Freiburg, 79110 Freiburg, Germany; 3 Université Clermont Auvergne, INRA, MEDIS, 63000 Clermont-Ferrand, France; 4 Université Clermont Auvergne, CNRS, LMGE, 63000 Clermont–Ferrand, France; 5 Université Clermont Auvergne, CNRS, LIMOS, 63000 Clermont–Ferrand, France; 6 Department of Bioinformatics, Erasmus University Medical Center, Rotterdam, 3015 CE, Netherlands

**Keywords:** metagenomics, metataxonomics, user-friendly, Galaxy, Docker, microbiota, training

## Abstract

**Background:**

New generations of sequencing platforms coupled to numerous bioinformatics tools have led to rapid technological progress in metagenomics and metatranscriptomics to investigate complex microorganism communities. Nevertheless, a combination of different bioinformatic tools remains necessary to draw conclusions out of microbiota studies. Modular and user-friendly tools would greatly improve such studies.

**Findings:**

We therefore developed ASaiM, an Open-Source Galaxy-based framework dedicated to microbiota data analyses. ASaiM provides an extensive collection of tools to assemble, extract, explore, and visualize microbiota information from raw metataxonomic, metagenomic, or metatranscriptomic sequences. To guide the analyses, several customizable workflows are included and are supported by tutorials and Galaxy interactive tours, which guide users through the analyses step by step. ASaiM is implemented as a Galaxy Docker flavour. It is scalable to thousands of datasets but also can be used on a normal PC. The associated source code is available under Apache 2 license at https://github.com/ASaiM/framework and documentation can be found online (http://asaim.readthedocs.io).

**Conclusions:**

Based on the Galaxy framework, ASaiM offers a sophisticated environment with a variety of tools, workflows, documentation, and training to scientists working on complex microorganism communities. It makes analysis and exploration analyses of microbiota data easy, quick, transparent, reproducible, and shareable.

## Findings

### Background

The study of microbiota and microbial communities has been facilitated by the evolution of sequencing techniques and the development of metataxonomics, metagenomics, and metatranscriptomics. These techniques are giving insight into taxonomic profiles and genomic components of microbial communities. However, meta'omic data exploitation is not trivial due to the large amount of data, their complexity, the incompleteness of reference databases, and the difficulty to find, configure, use, and combine the dedicated bioinformatics tools, etc. Hence, to extract useful information, a sequenced microbiota sample has to be processed by sophisticated workflows with numerous successive bioinformatics steps [1]. Each step may require execution of several tools or software. For example, to extract taxonomic information with the widely used QIIME [2] or Mothur [3], at least 10 different tools with at least four parameters each are needed. Designed for amplicon data, both QIIME and Mothur cannot be directly applied to shotgun metagenomics data. In addition, the tools can be complex to use; they are command-line tools and may require extensive computational resources (memory, disk space). In this context, selecting the best tools, configuring them to use the correct parameters and appropriate computational resources, and combining them together in an analysis chain is a complex and error-prone process. These issues and the involved complexity are prohibiting scientists from participating in the analysis of their own data. Furthermore, bioinformatics tools are often manually executed and/or patched together with custom scripts. These practices raise doubts about a science gold standard: reproducibility [3, 4]. Web services and automated pipelines such as MG-RAST [5] and EBI metagenomics [6] offer solutions to the accessibility issue. However, these web services work as a black box and are lacking in transparency, flexibility, and even reproducibility as the version and parameters of the tools are not always available. Alternative approaches to improve accessibility, modularity, and reproducibility can be found in open-source workflow systems such as Galaxy [6-8]. Galaxy is a lightweight environment providing a web-based, intuitive, and accessible user interface to command-line tools, while automatically managing computation and transparently managing data provenance and workflow scheduling [6-8]. More than 5,500 tools can be used inside any Galaxy environment. For example, the main Galaxy server [9] integrates many genomic tools, and the few integrated metagenomics tools such as Kraken [10] or VSearch [11] have been showcased in the published windshield splatter analysis [12]. The tools can also be selected and combined to build Galaxy flavors focusing on specific type of analysis, for example, the Galaxy RNA workbench [13] or the specialized Galaxy server of the Huttenhower lab [14]. However, none of these solutions is dedicated to microbiota data analysis in general and with the community-standard tools.

In this context, we developed ASaiM (Auvergne Sequence analysis of intestinal Microbiota, RRID:SCR_015878), an Open-Source opinionated Galaxy-based framework. It integrates more than 100 tools and several workflows dedicated to microbiota analyses with an extensive documentation [15] and training support.

### Goals of ASaiM

ASaiM is developed as a modular, accessible, redistributable, sharable, and user-friendly framework for scientists working with microbiota data. This framework is unique in combining curated tools and workflows and providing easy access and support for scientists.

ASaiM is based on four pillars: (1) easy and stable dissemination via Galaxy, Docker, and Conda, (2) a comprehensive set of microbiota-related tools, (3) a set of predefined and tested workflows, and (4) extensive documentation and training to help scientists in their analyses.

### A framework built on the shoulders of giants

The ASaiM framework is built on existing tools and infrastructures and combines all their forces to create an easily accessible and reproducible analysis platform.

ASaiM is implemented as a portable virtualized container based on the Galaxy framework [8]. Galaxy provides researchers with means to reproduce their own workflows analyses, rerun entire pipelines, or publish and share them with others. Based on Galaxy, ASaiM is scalable from single CPU installations to large multi-node high performance computing environments and manages efficiently job submission as well as memory consumption of the tools. Deployments can be achieved by using a pre-built ASaiM Docker image, which is based on the Galaxy Docker project [16]. This ASaiM Docker flavour is customized with a variety of selected tools, workflows, interactive tours, and data that have been added as additional layers on top of the generic Galaxy Docker instance. The containerization keeps the deployment task to a minimum. The selected Galaxy tools are automatically installed from the Galaxy ToolShed [17] using the Galaxy API BioBlend [18], and the installation of the tools and their dependencies are automatically resolved using packages available through Bioconda [19]. To populate ASaiM with the selected microbiota tools, we migrated the 12 tools/suites of tools and their dependencies to Bioconda (e.g., HUMAnN2), integrated 16 suites (>100 tools) into Galaxy (e.g., HUMAn2 or QIIME with its approximately 40 tools), and updated the already available ones (Table 1).

**Table 1:** Available tools in ASaiM

Section	Subsection	Tools
File and meta tools	Data retrieval	EBISearch [20], ENASearch [21], SRA Tools
	Text manipulation	Tools from Galaxy ToolShed
	Sequence file manipulation	Tools from Galaxy ToolShed
	BAM/SAM file manipulation	SAM tools [22-24]
	BIOM file manipulation	BIOM-Format tools [25]
Genomics tools	Quality control	FastQC [26], PRINSEQ [27], Trim Galore! [28, Trimmomatic [29], MultiQC [30]
	Clustering	CD-Hit [31], Format CD-HIT outputs
	Sorting and prediction	SortMeRNA [32], FragGeneScan [33]
	Mapping	BWA [34], Bowtie [35]
	Similarity search	NCBI Blast+ [36, 37], Diamond [38]
	Alignment	HMMER3 [39]
Microbiota dedicated tools	Metagenomics data manipulation	VSEARCH [11], Nonpareil [40]
	Assembly	MEGAHIT [41], metaSPAdes [42], metaQUAST [43], VALET [44]
	Metataxonomic sequence analysis	Mothur [3], QIIME [2]
	Taxonomy assignation on WGS sequences	MetaPhlAn2 [45], Format MetaPhlan2, Kraken [10]
	Metabolism assignation	HUMAnN2 [46], Group HUMAnN2 to GO slim terms [47], Compare HUMAnN2 outputs, PICRUST [48], InterProScan
	Combination of functional and taxonomic results	Combine MetaPhlAn2 and HUMAnN2 outputs
	Visualization	Export2graphlan [49], GraPhlAn [50], KRONA [51]

This table presents the tools, organized in sections and subsections to help users. A more detailed table of the available tools and some documentation can be found in the online documentation (http://asaim.readthedocs.io/en/latest/tools/).

### Tools for microbiota data analyses

The tools integrated in ASaiM can be seen in Table 1. They are expertly selected for their relevance with regard to microbiota studies, such as Mothur (mothur, RRID:SCR_011947) [3], QIIME (QIIME, RRID:SCR_008249) [2], MetaPhlAn2 (MetaPhlAn, RRID:SCR_004915) [45], HUMAnN2 [46], or tools used in existing pipelines such as EBI Metagenomics’ one. We also added general tools used in sequence analysis such as quality control, mapping, or similarity search tools.

An effort in development was made to integrate these tools into Conda and the Galaxy environment (>100 tools integrated) with the help and support of the Galaxy community. We also developed two new tools to search and get data from EBI Metagenomics and ENA databases (EBISearch [20] and ENASearch [21]) and a tool to group HUMAnN2 outputs into Gene Ontology Slim Terms [47]. Tools inside ASaiM are documented [15] and organized to make them findable.

### Diverse source of data

An easy way to upload user-data into ASaiM is provided by a web interface or more sophisticatedly via FTP or SFTP. On the top, we added specialised tools that can interact with external databases like NCBI, ENA, or EBI Metagenomics to query them and download data into the ASaiM environment.

### Visualization of the data

An analysis often ends with summarizing figures that conclude and represent the findings. ASaiM includes standard interactive plotting tools to draw bar charts and scatter plots for all kinds of tabular data. Phinch visualization [52] is also included to interactively visualize and explore any BIOM file and generate different types of ready-to-publish figures. We also integrated two other tools to explore and represent the community structure: KRONA [51] and GraPhlAn [53]. Moreover, as in any Galaxy instance, other visualizations are included such as Phyloviz [54] for phylogenetic trees or the genome browser Trackster [55] for visualizing SAM/BAM, BED, GFF/GTF, WIG, bigWig, bigBed, bedGraph, and VCF datasets.

### Workflows

Each tool can be used separately in an explorative manner, the Galaxy tool form helping users in setting meaningful parameters. Tools can be also orchestrated inside workflows using the powerful Galaxy workflow manager. To assist in microbiota analyses, several workflows, including a few well-known pipelines, are offered and documented (tools and their default parameters) in ASaiM. These workflows can be used as is; customized either on the fly to tune the parameters or globally to change the tools, their order, and their default parameters; or even used as subworkflows. Moreover, users can also design novel meaningful workflows via the Galaxy workflow interface using the >100 available tools.

### Analysis of raw metagenomic or metatranscriptomic shotgun data

**Figure 1:**
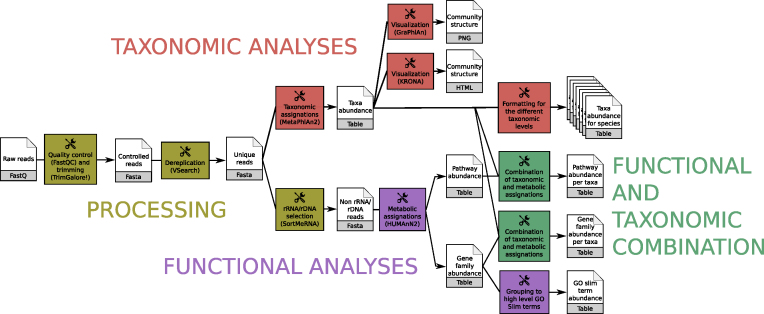
Main ASaiM workflow to analyze raw sequences. This workflow takes as input a dataset of raw shotgun sequences (in FastQ format) from microbiota, preprocess it (yellow boxes), extracts taxonomic (red boxes) and functional (purple boxes) assignations, and combines them (green boxes). Image available under CC-BY license (https://doi.org/10.6084/m9.figshare.5371396.v3).

The workflow quickly produces, from raw metagenomic or metatranscriptomic shotgun data, accurate and precise taxonomic assignations, wide extended functional results, and taxonomically related metabolism information (Fig. 1). This workflow consists of (i) processing with quality control/trimming (FastQC and Trim Galore!) and dereplication (VSearch [11]); (ii) taxonomic analyses with assignation (MetaPhlAn2 [45]) and visualization (KRONA, GraPhlAn); (iii) functional analyses with metabolic assignation and pathway reconstruction (HUMAnN2 [46]); (iv) functional and taxonomic combination with developed tools combining HUMAnN2 and MetaPhlAn2 outputs.

**Figure 2:**
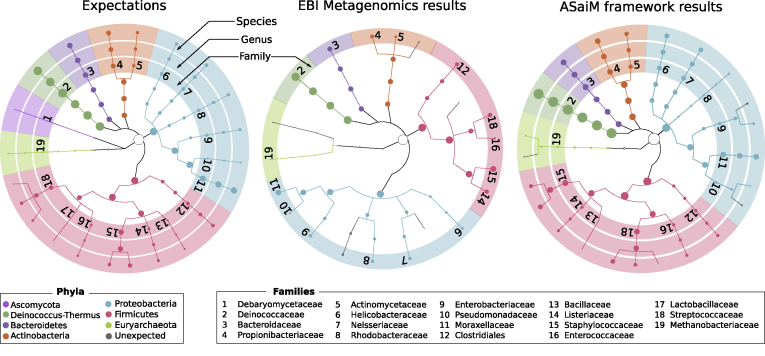
Comparisons of the community structure for SRR072233. This figure compares the community structure between the expectations (mapping of the sequences on the expected genomes), data found on EBI Metagenomics database (extracted with the EBI Metagenomics pipeline), and the results of the main ASaiM workflow (Fig. 1).

**Figure 3:**
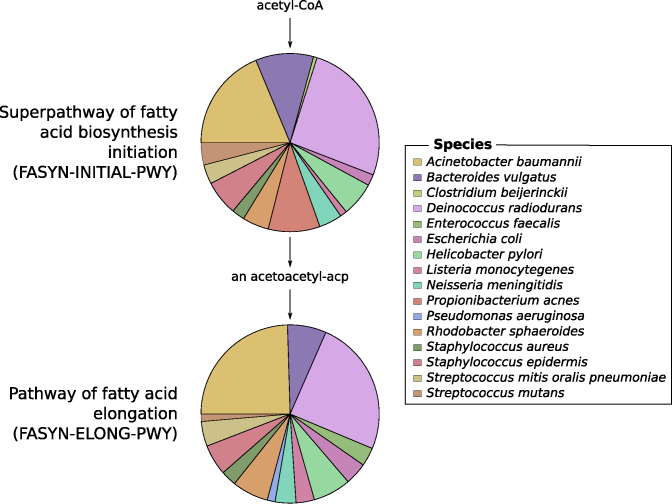
Example of an investigation of the relation between community structure and functions. The involved species and their relative involvement in fatty acid biosynthesis pathways have been extracted with ASaiM workflow (Fig. 1) for SRR072233.

This workflow has been tested on two mock metagenomic datasets with controlled communities (Supplementary material). We have compared the extracted taxonomic and functional information to such information extracted with the EBI metagenomics’ pipeline and to the expectations from the mock datasets to illustrate the potential of the ASaiM workflow. With ASaiM, we generate accurate and precise data for taxonomic analyses (Fig. 2), and we can access information at the level of the species. More functional information (e.g., gene families, gene ontologies, pathways) are also extracted with ASaiM compared to the ones available on EBI metagenomics. With this workflow, we can go one step further and investigate which taxons are involved in a specific pathway or a gene family (e.g., involved species and their relative involvement in different step of fatty acid biosynthesis pathways, Fig. 3).

For the tests, ASaiM was deployed on a computer with Debian GNU/Linux System, 8 cores Intel(R) Xeon(R) at 2.40 GHz and 32 Go of RAM. The workflow processed the 1,225,169 and 1,386,198 454 GS FLX Titanium reads of each datasets, with a stable memory usage, in 4h44 and 5h22 respectively (Supplementary material). The execution time is logarithmically linked to the input data size. With this workflow, it is then easy and quick to process raw microbiota data and extract diverse useful information.

### Assembly of metagenomics data

Microbiota data usually come with quite short reads. To reconstruct genomes or to get longer sequences for further analysis, microbiota sequences have to be assembled with dedicated metagenome assemblers. To help in this task, two workflows have been developed in ASaiM, each one using one of the well-performing assemblers [56-62]: MEGAHIT [41] and MetaSPAdes [42]. Both workflows consists of: (1) processing with quality control/trimming (FastQC and Trim Galore!); (2) assembly with either MEGAHIT or MetaSPAdes; (3) estimation of the assembly quality statistics with MetaQUAST [43]; (4) identification of potential assembly error signature with VALET; and (5) determination of percentage of unmapped reads with Bowtie2 (Bowtie, RRID:SCR_005476) [36] combined with MultiQC [30] to aggregate the results.

### Analysis of metataxonomic data

To analyze amplicon or internal transcribed spacer data, the Mothur and QIIME tool suites are available in ASaiM. We integrated the workflows described in tutorials of Mothur and QIIME as an example of metataxonomic data analyses as well as support for the training material.

### Running as in EBI Metagenomics

As the tools used in the EBI Metagenomics pipeline (version 3) are also available in ASaiM, we integrate them in a workflow with the same steps as the EBI Metagenomics pipeline. Analyses made in the EBI Metagenomics website can be then reproduced locally without having to wait for availability of EBI Metagenomics or to upload any data on EBI Metagenomics. However, the parameters must be defined by the user, as we cannot find them on EBI Metagenomics documentation. In ASaiM, the entire provenance and every parameter are tracked to guarantee the reproducibility.

### Documentation and training

A tool or software is easier to use if it is well documented. Hence, extensive documentation helps the users to be familiar with the tool and also prevents mis-usage. For ASaiM, we developed an extensive online documentation [15] , mainly to explain how to use it, how to deploy it, which tools are integrated with small documentation about these tools, which workflows are available, and how to use them.

In addition to this online documentation, training materials have been developed. Some Galaxy interactive tours are included inside the Galaxy instance to guide users through entire microbiota analyses in an interactive (step-by-step) way. We also developed several step-by-step tutorials to explain the concepts of microbiota analyses, the different tools and parameters, and ASaiM workflows with toy datasets. Hosted within the Galaxy Training Material [63], the tutorials are available online at [64] and also directly accessible from ASaiM and its documentation for self-training. These tutorials and ASaiM have been used during several workshops on metagenomics data analysis and some undergraduate courses to explain and use the EBI Metagenomics workflow in a reproducible way. ASaiM is also used as support for a citizen science and education project (BeerDeCoded [65]).

### Installation and running ASaiM

Running the containerized ASaiM simply requires the user to install Docker and to start the ASaiM image with:

$ docker run -d -p 8080:80 quay.io/bebatut/asaim-framework:latest

As Galaxy, ASaiM is production ready and can be configured to use external accessible computer clusters or cloud environments. It is also possible and easy to install all or only a subset of tools of the ASaiM framework on existing Galaxy instances, as we did on the European Galaxy instance [66]. More details about the installation and the use of ASaiM are available on the online documentation [15].

## Conclusion

ASaiM provides a powerful framework to easily and quickly analyze microbiota data in a reproducible, accessible, and transparent way. Built on a Galaxy instance wrapped in a Docker image, ASaiM can be easily deployed with its extensive set of tools and their dependencies, saving users from the hassle of installing all software. These tools are complemented with a set of predefined and tested workflows to address the main questions of microbiota research (assembly, community structure, and function). All these tools and workflows are extensively documented online [15] and supported by interactive tours and tutorials.

With this complete infrastructure, ASaiM offers a sophisticated environment for microbiota analyses to any scientist while promoting transparency, sharing, and reproducibility.

## Methods

For the tests, ASaiM was deployed on a computer with Debian GNU/Linux System, 8 cores Intel(R) Xeon(R) at 2.40 GHz and 32 Go of RAM. The workflow has been run on two mock community samples of the Human Microbiome Project containing a genomic mixture of 22 known microbial strains. The details of comparison analyses are described in the Supplementary Material.

## Availability of supporting data

Archival copies of the code and mock data are available in the *GigaScience* GigaDB repository [67].

## Availability of supporting source code and requirements

- Project name: ASaiM
- Project home page: https://github.com/ASaiM/framework
- Operating system(s): Platform independent
- Other requirements: Docker
- License: Apache 2
- RRID:SCR_015878GTN

All tools described herein are available in the Galaxy Toolshed (https://toolshed.g2.bx.psu.edu). The Dockerfile to automatically deploy ASaiM is provided in the GitHub repository (https://github.com/ASaiM/framework) and a pre-built Docker image is available at https://quay.io/repository/bebatut/asaim-framework.

## Supplementary Material

GIGA-D-17-00230_Original_Submission.pdf

GIGA-D-17-00230_Revision_1.pdf

GIGA-D-17-00230_Revision_2.pdf

Response_to_Reviewer_Comments_Original_Submission.pdf

Response_to_Reviewer_Comments_Revision_1.pdf

Reviewer_1_Report_(Original_Submission) -- Konstantinos Krampis, PhD 9/13/2017 Reviewed

Reviewer_2_Report_(Original_Submission) -- Efthymios Ladoukakis, Ph.D 10/4/2017 Reviewed

Reviewer_2_Report_(Revision_1) -- Efthymios Ladoukakis, Ph.D 1/23/2018 Reviewed

Reviewer_3_Report_(Original_Submission) -- Alessia Visconti 10/9/2017 Reviewed

Reviewer_3_Report_(Revision_1) -- Alessia Visconti 1/29/2018 Reviewed

Supplemental material

## Abbreviations

API: application programming interface; AsaiM: Auvergne Sequence analysis of intestinal Microbiota; CPU: central processing unit; Galaxy Training Network.
