# Supplementary material for: ASaiM: a Galaxy-based framework to analyze microbiota data
Source: Gigascience. 2018 May 15;7(6):giy057. doi: 10.1093/gigascience/giy057 (PMC6007547; doi:10.1093/gigascience/giy057)
Supplement: GIGA-D-17-00230_Revision_1.pdf [file giy057_giga-d-17-00230_revision_1.pdf]

GigaScience

ASaiM: a Galaxy-based framework to analyze microbiota data

--Manuscript Draft--

**Manuscript Number:**	GIGA-D-17-00230R1
**Full Title:**	ASaiM: a Galaxy-based framework to analyze microbiota data
**Article Type:**	Technical Note
**Funding Information:**	Conseil Régional d'Auvergne — Dr Bérénice Batut<br>European Regional Development Fund — Not applicable
**Abstract:**	New generation of sequencing platforms coupled to numerous bioinformatics tools has led to rapid technological progress in metagenomics and metatranscriptomics to investigate complex microorganism communities. Nevertheless, a combination of different bioinformatic tools remains necessary to draw conclusions out of microbiota studies. Modular and user-friendly tools would greatly improve such studies.<br><br>We therefore developed ASaiM, an Open-Source Galaxy-based framework dedicated to microbiota data analyses. ASaiM provides an extensive collection of tools to assemble, extract, explore and visualize microbiota information from raw metataxonomic, metagenomic or metatranscriptomic sequences. To guide the analyses, several customizable workflows are included and are supported by tutorials and Galaxy interactive tours, which guide users through the analyses step by step. ASaiM is implemented as Galaxy Docker flavour. It is scalable to thousands of datasets, but also can be used on a normal PC. The associated source code is available under Apache 2 license at https://github.com/ASaiM/framework and documentation can be found online (http://asaim.readthedocs.io).<br><br>Based on the Galaxy framework, ASaiM offers a sophisticated environment with a variety of tools, workflows, documentation and training to scientists working on complex microorganism communities. It makes analysis and exploration analyses of microbiota data easy, quick, transparent, reproducible and shareable.
**Corresponding Author:**	Bérénice Batut, Ph.D.<br>University of Freiburg<br>Freiburg, GERMANY
**Corresponding Author Secondary Information:**	
**Corresponding Author's Institution:**	University of Freiburg
**Corresponding Author's Secondary Institution:**	
**First Author:**	Bérénice Batut, Ph.D.
**First Author Secondary Information:**	
**Order of Authors:**	Bérénice Batut, Ph.D.
	Kévin Gravouil
	Clémence Defois
	Saskia Hiltemann
	Jean-François Brugère
	Eric Peyretaillade
	Pierre Peyret
**Order of Authors Secondary Information:**	
**Response to Reviewers:**	We thanked the editor and the reviewers for their suggestions and constructive critiques.

Powered by Editorial Manager® and ProduXion Manager® from Aries Systems Corporation

Editor

“Your manuscript "ASaiM: a Galaxy-based framework to analyze raw shotgun data from microbiota" (GIGA-D-17-00230) has been assessed by our reviewers. Although it is certainly of interest, we are unable to consider it for publication without some revisions. The reviewers have raised a number of points which we believe would improve the manuscript and may allow a revised version to be published in GigaScience. Particularly there are some suggestions to change the focus, so you will need to decide to focus purely on the shotgun sequencing, or take a broader approach and potentially change it to a more general toolkit (potentially also stressing the educative aspects too).”

We think that ASaiM should be general toolkit for the analysis of microbiota data. Indeed, it is currently used for diverse metagenomics projects (either shotgun or amplicon), like the Beer DeCoded project which analyzes the ITS sequences of the beer microbiota in a pedagogic way or the assembly of metagenomics datasets from EBI Metagenomics to extract CRISPR subtypes. So, we added some tools and workflows for ITS analysis and metagenomic assembly and are currently working on integration of binning tools. We also changed the title to indicate the general purpose of ASaiM: “ASaiM: a Galaxy-based framework to analyze microbiota data”.
To stress the educative aspects, we also added a short paragraph explaining in more detail how ASaiM is used for a citizen science project (Beer DeCoded) or in training courses, for example to understand and use the EBI metagenomic workbench in a reproducible way for teaching undergrads.

“In addition, we are now asking authors to register any new software application or pipeline in the SciCrunch.org database to receive a RRID (Research Resource Identification Initiative ID) number, and include this in your manuscript. This will facilitate tracking, reproducibility and re-use of your tool.”

The tool has been submitted to SciCrunch: RRID:SCR_015878. We added the information in the manuscript.

Reviewer #1

“Excellent paper, useful collections of tools, focused approach and well organized with great documentation.”

We thank the reviewer for this nice comment.

“Enough background for a software paper, my suggestion would be if you can mention a little more on metagenomics pipelines available on the main Galaxy server, in addition to an example of specialized Galaxy servers for metagenomics - for example the Metaphlan group they have such a specialized server: https://huttenhower.sph.harvard.edu/galaxy/”

We added in the introduction a sentence about the main Galaxy server and the metagenomics tools available there. We also mentioned the server of the Huttenhower lab. Moreover, we are in contact with the administrators of usegalaxy.org and we will ensure that all workflows and trainings will also work on their server.

“In addition I was really excited to see the provenance mentioned. Since the documentation is so extensive (and excellent!), perhaps the authors could add a section on how to save a docker container where data has been processed with their tool (also how to bundle the volumes with the data), so that the whole package and be distributed (and provide analysis provenance), to collaborators, with a publication etc.”

We tried to keep the documentation on the Docker usage simple and not redundant

with the already extensive documentation available for the Galaxy Docker project (https://github.com/bgruening/docker-galaxy-stable). In the online documentation, we added more links in the documentation to this Docker documentation, especially with the questions the reviewer asked, and added a sentence to refer to this online documentation in the manuscript.
To answer the question directly, it is possible to store, archive and share the entire /export folder of a Galaxy Docker image. This can then be easily shared, uploaded to Zenodo etc. and reused with any other Galaxy Docker container.

“Overall an excellent paper !”

Reviewer #2

“Some spelling errors:

line 56: blocking scientist -> blocking scientists
line 124 an web-interface -> a web-interface
line 135: visualization -> visualizations
line 135: such Phyloviz -> such as Phyloviz
line 157 Figure 2): we -> Figure2) and we
line 175-176: We integrate then also a workflow -> We also integrated them in a workflow

in report (supp. material) targeted abundances may be not reflect -> targeted abundances may not reflect”

Thanks for reporting these mistakes, we addressed all of them in the revised version.

Further remarks:

1) “The title is a bit lacking in context. ASAIM is clearly dedicated only towards the taxonomic and functional analysis of metagenomic data (either from amplicon sequencing or from shotgun sequencing). It would be beneficial for the reader to deduce that from the title.”

ASaiM is a community starting point for all people interested in metagenomic research. During the last months other tools related to metagenomic assembly as MetaSPAdes or MEGAHIT and some tools for binning were added, partially by the community, but also on request from collaborators. The objectives of ASaiM is to offer a comprehensive and general workbench for microbiota analysis and thus we would like to have a slightly more general title. However, we changed the title slightly to: “ASaiM: a Galaxy-based framework to analyze microbiota data”

2) “It's not quite clear the innovative part of the platform. Besides collecting all those preexisting tools in an organized manner under Galaxy's umbrella what was the added contribution of ASAIM's team? Did you develop new wrapper/parser scripts for some/all of these tools in order to integrate them with Galaxy? What is the added value of the 3 new tools you developped? The GO slim term tool seems to be one of the final tools (purple) in your workflow (is that correct?). What about the other two for searching EBI and ENA databases? Are they part of one of the workflows or just additional standalone tools?”

The ASaiM team migrated 12 tools/suites of tools and their dependencies to Bioconda (e.g. HUMAnN2, MetaPhlAn2, GraPhlAN), integrated 16 suites (>100 tools) into Galaxy (e.g. HUMAn2 or QIIME with its around forty tools), i.e. developing the wrappers for these tools. We also checked and updated the wrappers of the existing tools. Moreover, several Galaxy datatypes, (interactive) training material and a visualization were developed and integrated into Galaxy.
The 3 tools we developed were needed to close missing steps in workflows or to make it more convenient for users to access publicly available data.

The GO slim tool is used to aggregate the gene family abundances into GO terms and is indeed one of the final steps in the workflow.
The EBISearch and ENASearch tools are standalone tools to allow users to query ENA and EBI Metagenomics databases (data, metadata) and transfer to directly into Galaxy. They are not integrated into the one of our predefined workflows because the inputs of the workflows could be local data or data from external database such as ENA and we can not determine that before.
To complement the tools and workflows, the ASaiM team created also documentation and tutorials.

3) ”The comparison between ASAIM and EBI analysis seems rather trivial. It's not a comparison of the two platforms rather than a comparison of the two different tools they are using (QIIME and Metaphlan). It would make much more sense a comparison between EBI's workflow run in the exact same way as an ASAIM/Galaxy workflow with the same tools.”

We would like to do this, but currently it is not possible to know the exact parameters which are used in the EBI Metagenomics workflow. This latter workflow is, unfortunately, currently a blackbox in contrary to ASaiM whose one of the objectives is to make microbiota research more transparent and reproducible.

4) “The same goes for functional analysis (where you mention comparison is not feasible). You just present results derived from two different methods with no comparable points.”

For the same reason as stated above we are very limited in what we can compare. Moreover. the functional information are extracted with two different types of information. EBI Metagenomics extracts the InterProScan gene families. In ASaiM, we extract with HUMAnN2 the UniRef gene families. It complexifies any comparisons.

5) “In line 200 the command you state
docker run -d -p 8080:80 quay.io/bebatut/asaim

is different than the one stated in your webpage where the installation instructions are:

docker run -d -p 8080:80 quay.io/bebatut/asaim-framework

while the "asaim" command doesn't work (not authorized error) the "asaim-framework" seems to work”

We apologize for this mistake. We fixed the command mentioned in the manuscript to fit to the one in the instructions.

6) “In supplementary material report page 3 contains a table that is not well displayed”

Thanks for reporting this. We fixed the table.

7) “Installation was not succesful so actual testing of the tool was not possible. Installation in a new CentOS distribution (3.10.0-514) under a Virtuabox engine failed. It could be useful to mention in your docs how to install and start the docker engine before attempting to download the ASAIM package especially for those with little or no command line knowledge.”

As the installation of the Docker engine can vary between different operating systems and is changing over time we think the best way is to link to the upstream documentation under https://docs.docker.com/engine/installation. We also added a link to a video explaining how to use Kitematic for Galaxy Docker, for the non-linux users.

“At some point during the installation process there was an error saying:

"failed to register layer: ApplyLayer exit status 1 stdout: stderr: write /tool_deps/_conda/envs/__picrust@1.1.1/lib/python2.7/site-packages/mpi4py/MPI.so: no space left on device."

Not sure how that's possible with 34GB available free space. Does ASAIM include databases that take up more space than that? If that's the case you should probably include that in the Requirements section in your webpage and inform the reviewers as well in order for us to be able to succesfully install and properly test it."

We apologize for this unfortunate experience.
ASaiM includes numerous tools and reference databases for HUMAnN2 and MetaPhlAn2 and this increases the required disk space to 40GB. We forgot to mention this in our documentation and addressed this issue. In the meantime we are working hard to make this experience easier in the near future. The latest ASaiM Docker release already supports the CVMFS filesystem, with which we can easily mount in TB of reference data into every image. The data is then only downloaded if it get accessed by tools. We will extend this over the next releases.

Reviewer #3

"The manuscript describes an alternative workflow for the processing of shotgun metagenomics and metatranscriptomic data, called ASaiM.
ASaiM integrates multiple tools for the analysis and manipulation of raw metagenomics and metatranscriptomic data, that are available, both as single tools and combined in multiple pipelines, within the Galaxy workflow and with a Docker and conda support. ASaiM comes with a very impressive documentation and it is of high importance in the metagenomics community, where most of the analyses are carried out using in-house scripts that, as pointed out by the authors, hinder reproducibility.

However, several other metagenomics pipelines are already available: MG-RAST and the EBI metagenomics pipeline, that the authors briefly discuss in the Introduction, but also MOCAT2, MetAMOS, and another Galaxy metagenomic pipeline. How does ASaiM compare within this wider ecosystem? MOCAT2, for instance, comes with a set of preset parameters, stored in a single file, that already improve reproducibility, and the EBI metagenomics pipeline clearly shows the software version (e.g., https://www.ebi.ac.uk/metagenomics/pipelines/3.0), allowing provenance."

Provenance is way more than just the version of the used tool in a workflow. Every single parameter or the version of the used reference database can have a huge influence on the results.
But even if the various webservers would allow for a complete provenance it's hard or impossible to run those pipelines locally or on a local cluster. ASaiM is changing this by offering all tools of the different pipelines in one workbench, that can be deployed locally, on a cluster or in a cloud. The different pipelines can even be mixed if necessary, allowing for a unmatched flexibility and reproducibility. Moreover, ASaiM will ensure that the entire provenance is tracked and every single parameter, the exact version of the tools and input data is tracked and can be reproduced and compared.
The reviewer mentioned MOCAT2. This command line tool is a great tool. However, it focuses only on metagenomic data (not for metataxonomic or metatranscriptomic data, as we would like) and its command-line use is a limitation for its use for all scientists working with microbiota data. We will work on integrating it into ASaiM.
With EBI Metagenomics, the versions of software are available but not the parameters or the versions of databases used. For this reason, we did not set up any parameters in the workflow developed to reproduce the one on EBI Metagenomics. We think it is a big issue for reproducibility, as the parameters and the databases can have a big impact on the results.

"Also, the authors point out that the main problems in analysing metagenomics data are, first, the selection and configuration of the necessary tools, then the definition of the correct computational resources, and, finally, the definition of a correct analysis workflow. However, in this reviewer's opinion, ASaiM does not fully address these

Powered by Editorial Manager® and ProduXion Manager® from Aries Systems Corporation

limitations. The authors implement about 25 tools for the processing of metagenomics data but give little explanation on the reasons these specific tools have been selected, or which tools should be used when multiple tools within the same class are available. Novices in the field would surely appreciate these pieces of information as a way to select the correct software for the problem at hand."

Information about this was added in the documentation and in the tutorials we developed with the Galaxy Training Network. We follow the idea to offer a variety of different tools, even if they have overlapping functionality, to enable a lot of flexibility and freedom in data analysis. In this regard we want to offer easy access to a lot of different software. If a user needs guidance and the amount of tools is just overwhelming, we provide workflows for different use-cases and training material, in which we choose specialised tools and leave other out. However, we think the power of an analysis should be in the hand of the user and different steps in a workflow should/could be interchangeable.

"Regarding the workflows included in ASaiM, one is a reimplementation of the EBI workflow, one cannot be used for analysing metagenomic shotgun data, and only one is novel (that this reviewer supposes is the one called very generally "ASaiM"). This reviewer would suggest the authors to focus more on describing this novel workflow, and to remove all the references to QIIME and Mothur tools (or to 16S data analysis in general) since these are not able to analyse shotgun metagenomics data and may generate confusion."

We think that ASaiM should be general toolkit for the analysis of microbiota data, not only for shotgun data. Microbiota analyses are usually not only focused on one type of analysis (metagenomics, metatranscriptomics, metataxonomics). We usually need to combine tools developed for different purposes to analyze our data. For example to compute abundance statistics such as alpha or beta diversity, we can apply the QIIME tools on the BIOM files generated by metagenomics tools such as MetaPhlAn. ASaiM is currently used in diverse microbiota projects (shotgun, amplicon and ITS data). We would like then to keep the mention of the QIIME and Mothur tools, and their workflow. We also added two workflows for metagenomic assembly (one using MEGAHIT and one using MetaSPAdes), including quality control, assembly and assembly checking (statistics, mapping and identification of potential assembly error signatures).

"For instance, it would be interesting to know how the workflow can be customised, whether default parameters are available and how they have been selected, and have more detailed and exhaustive information on time and computational requirements (and not only on two samples)."

We clarified the customization of workflows in the manuscript:
"To assist in microbiota analyses, several default workflows are proposed and documented (tools, default parameters) in ASaiM. These workflows can be used as they are, customized either on the fly to tune the parameters or globally to change the tools, their order and their default parameters, or even used as subworkflows.".
We added more details in the documentation and also in the tutorials about the choices of default parameters for the tools.
Exhaustive information on time and computational requirements are difficult to extract. They greatly depend on the input data. Currently for the shotgun workflow, the main time-consuming task is HUMAnN2 and its execution time is not linear with input size. We added a sentence in the manuscript to mention that.
In general ASaiM is configured by default to run on normal personal computers, but because ASaiM is utilizing the Galaxy framework all tools and workflows can be easily configured to scale out and use entire clusters or other available compute resources. Here, we are referring to the upstream documentation of Galaxy or the Docker Galaxy project.

"Also, it is not clear what improvements are brought by ASaiM and what are due to the usage of Galaxy (reproducibility, provenance, being user-friendly), or of HUMAnN2 (ability to infer the taxonomic profiles up to the species level, availability of genes and

Powered by Editorial Manager® and ProduXion Manager® from Aries Systems Corporation

pathways abundances tables). For instance, how the proposed 'functional and taxonomic combination analysis' block differs with that proposed within the HUMAnN2 pipeline?"

ASaiM is a collection of existing tools that are combined into a dedicated Galaxy instance. On top of these tools we have build workflows and training material. Thanks to Galaxy and Docker, ASaiM can be easily shipped, deployed, but also customized for anyone. The ASaiM team maintains the tools, updates them, integrates new tools (> 100), datatypes and visualizations and develops documentation and training to help researchers to deal with microbiota data. We clarified the manuscript in this direction. The "functional and taxonomic combination analysis" block is the Galaxy implementation of the HUMAnN2 pipeline, but inside a workflow to help its execution on many samples and after several pre-processing steps (quality control, sorting, MetaPhlAn2), without the need to care about the computational details. It is a turnkey solution.

"More in general, this reviewer's main concern regards the focus of the manuscript. Are the authors interested in presenting the Galaxy implementation of a variety of metagenomics tools? Or to present a novel reproducible pipeline for the analysis of metagenomics data? Are they interested in metagenomics or metagenetics (16S) analysis? In this reviewer's opinion, the manuscript would surely benefit in focusing on a single message, while additional features (such as the analysis of metagenetics data) should be only briefly mentioned."

We are interesting in presenting ASaiM as an environment for people working on any type of microbiota data: a Galaxy implementation including a variety of microbiota related tools, workflow, documentation and training, whch is easy to distribute with its Docker image, for example for a publication of an analysis. We tried to make this message clearer in the manuscript, with for example a slightly different title "ASaiM: a Galaxy-based framework to analyze microbiota data"

"The manuscript includes some imprecision, with several concepts repeated multiple times, and would surely benefit from a proofreading by a native speaker:"

1. "Lines 40-43. Metagenomics and metatranscriptomics techniques do not allow to get insight into metabolic components, but only on the inferred functions of the micro-organisms present in one sample (as done, for instance, by HUMAnN2). To measure the metabolic components, one should use another approach, namely metametabolomics. It is also not clear what 'phylogenetic properties' are. Do the authors mean taxonomical profiles?"

We changed the sentence to clarify it: "These techniques are giving insight into taxonomic profiles and genomic components of microbial communities."

2. "Line 44. The authors mention 'high variability'. What is the feature showing this 'high variability'?"

High variability is referring to the diversity of organisms in one sample, uneven sequencing depth of the different organisms and other things that makes metagenomic research hard. We changed the word to use "their complexity".

3. "Line 52. Can the authors give examples of what they call 'computational resources specially for the metagenomics datasets'?"

We meant need for lot of memory and disk space, the use of cluster or cloud. They are not specific for metagenomic datasets, but probably highly required for metagenomics. We changed the sentence to:
"They are command-line tools and may require extensive computational resources (memory, disk space)".

Powered by Editorial Manager® and ProduXion Manager® from Aries Systems Corporation

	4. “Line 140. What is a 'data reduction step'?”<br><br>A data reduction step is the reduction of the input data: removal of bad quality sequences and trimming, removal of duplicated sequences (dereplication), sorting of the sequences. We removed this term, to avoid confusion.<br><br>4. “This reviewer suggests removing the 'Installation and running section' and simply refers to the documentation, as done in other cases.”<br><br>We decided to have this section because it shows that using ASaiM is not really difficult and also to mention that tools and workflows can be added to any already existing Galaxy instance. We significantly shortened this section and referenced the documentation. Thanks for this recommendation.
**Additional Information:**	
**Question**	**Response**
Are you submitting this manuscript to a special series or article collection?	No
**Experimental design and statistics**<br><br>Full details of the experimental design and statistical methods used should be given in the Methods section, as detailed in our Minimum Standards Reporting Checklist. Information essential to interpreting the data presented should be made available in the figure legends.<br><br>Have you included all the information requested in your manuscript?	Yes
**Resources**<br><br>A description of all resources used, including antibodies, cell lines, animals and software tools, with enough information to allow them to be uniquely identified, should be included in the Methods section. Authors are strongly encouraged to cite Research Resource Identifiers (RRIDs) for antibodies, model organisms and tools, where possible.<br><br>Have you included the information requested as detailed in our Minimum Standards Reporting Checklist?	Yes
**Availability of data and materials**<br><br>All datasets and code on which the conclusions of the paper rely must be either included in your submission or deposited in publicly available repositories	Yes

(where available and ethically appropriate), referencing such data using a unique identifier in the references and in the “Availability of Data and Materials” section of your manuscript.<br><br>Have you have met the above requirement as detailed in our Minimum Standards Reporting Checklist?	

Manuscript Click here to download Manuscript ASaiM_GigaSciences.docx

ASaiM: a Galaxy-based framework to analyze microbiota data

Bérénice Batut[1,*], Kévin Gravouil[2,3,4], Clémence Defois[2], Saskia Hiltemann[5], Jean-François Brugère[2], Eric Peyretaillade[2] and Pierre Peyret[2,*]

Author affiliations

[1]Bioinformatics Group, Department of Computer Science, University of Freiburg, Germany

[2]Université Clermont Auvergne, INRA, MEDIS, F-63000 Clermont-Ferrand, France

[3]Université Clermont Auvergne, CNRS, LMGE, F-63000 Clermont–Ferrand, France

[4]Université Clermont Auvergne, CNRS, LIMOS, F-63000 Clermont–Ferrand, France

[5]Department of Bioinformatics, Erasmus University Medical Center, Rotterdam, 3015 CE, Netherlands

Correspondence should be addressed to B.B. (berenice.batut@gmail.com) and P.P. (pierre.peyret@uca.fr)

Abstract

Background

New generation of sequencing platforms coupled to numerous bioinformatics tools has led to rapid technological progress in metagenomics and metatranscriptomics to investigate complex microorganism communities. Nevertheless, a combination of different bioinformatic tools remains necessary to draw conclusions out of microbiota studies. Modular and user-friendly tools would greatly improve such studies.

Findings

We therefore developed ASaiM, an Open-Source Galaxy-based framework dedicated to microbiota data analyses. ASaiM provides an extensive collection of tools to assemble, extract, explore and visualize microbiota information from raw metataxonomic, metagenomic or metatranscriptomic sequences. To guide the analyses, several customizable workflows are included and are supported by tutorials and Galaxy interactive tours, which guide users through the analyses step by step. ASaiM is implemented as Galaxy Docker flavour. It is scalable to thousands of datasets, but also can be used on a normal PC. The associated source code is available under Apache 2 license at https://github.com/ASaiM/framework and documentation can be found online (http://asaim.readthedocs.io).

Conclusions

Based on the Galaxy framework, ASaiM offers a sophisticated environment with a variety of tools, workflows, documentation and training to scientists working on complex microorganism communities. It makes analysis and exploration analyses of microbiota data easy, quick, transparent, reproducible and shareable.

Keywords

Metagenomics, Metataxonomics, User-friendly, Galaxy, Docker, Microbiota,

Findings

Background

The study of microbiota and microbial communities has been facilitated by the evolution of sequencing techniques and the development of metataxonomics, metagenomics and metatranscriptomics. These techniques are giving insight into taxonomic profiles and genomic components of microbial communities. However, meta'omic data exploitation is not trivial due to the large amount of data, their complexity, the incompleteness of reference databases, the difficulty to find, configure, use and combine the dedicated bioinformatics tools, etc. Hence, to extract useful information, a sequenced microbiota sample has to be processed by sophisticated workflows with numerous successive bioinformatics steps [1]. Each step may require execution of several tools or software. For example, to extract taxonomic information with the widely used QIIME [2] or Mothur [3], at least 10 different tools with at least 4 parameters each are needed. Designed for amplicon data, both QIIME and Mothur can not be directly applied to shotgun metagenomics data. In addition, the tools can be complex to use; they are command-line tools and may require extensive computational resources (memory, disk space). In this context, selecting the best tools, configuring them to use the correct parameters and appropriate computational resources and combining them together in an analysis chain is a complex and error-prone process. These issues and the involved complexity are prohibiting scientists from participating in the analysis of their own data. Furthermore, bioinformatics tools are often manually executed and/or patched together with custom scripts. These practices raise doubts about a science gold standard: reproducibility [3,4]. Web services and automated pipelines such as MG-RAST [5] and EBI metagenomics [6] offer solutions to the accessibility issue. However, these web services

work as a black box and are lacking in transparency, flexibility and even reproducibility as the version and parameters of the tools are not always available. Alternative approaches to improve accessibility, modularity and reproducibility can be found in open-source workflow systems such as Galaxy [6–8]. Galaxy is a lightweight environment providing a web-based, intuitive and accessible user interface to command-line tools, while automatically managing computation and transparently managing data provenance and workflow scheduling [6–8]. More than 4,500 tools can be used inside any Galaxy environment. For example, the main Galaxy server (http://usegalaxy.org) integrates many genomic tools whose few metagenomics tools such as Kraken [9] or VSearch [10] and was used for example in the windshield splatter analysis [11]. The tools can also be selected and combined to build Galaxy flavors focusing on specific type of analysis, *e.g.* the Galaxy RNA workbench [12] or the specialized Galaxy server of the Huttenhower lab (http://huttenhower.sph.harvard.edu/galaxy). However, none of these solutions are dedicated to microbiota data analysis in general, with the community-standard tools.

In this context, we developed ASaiM (Auvergne Sequence analysis of intestinal Microbiota, RRID:SCR_015878), an Open-Source opinionated Galaxy-based framework. It integrates more than 100 tools and several workflows dedicated to microbiota analyses with an extensive documentation (http://asaim.readthedocs.io) and training support.

Goals of ASaiM

ASaiM is developed as a modular, accessible, redistributable, sharable and user-friendly framework for scientists working with microbiota data. This framework is unique in combining curated tools and workflows and providing easy access and support for scientists.

ASaiM is based on four pillars: 1) easy and stable dissemination via Galaxy, Docker and Conda, 2) a comprehensive set of microbiota related tools, 3) a set of predefined and tested workflows, and 4) extensive documentation and training to help scientists in their analyses.

A framework built on the shoulders of giants

The ASaiM framework is built on existing tools and infrastructures and combine all their forces to create an easily accessible and reproducible analysis platform. ASaiM is implemented as a portable virtualized container based on Galaxy framework [8]. Galaxy provides researchers with means to reproduce their own workflows analyses, rerun entire pipelines, or publish and share them with others. Based on Galaxy, ASaiM is scalable from single CPU installations to large multi-node high performance computing environments. Deployments can be achieved by using a pre-built ASaiM Docker image, which is based on the Galaxy Docker project (http://bgruening.github.io/docker-galaxy-stable) or by installing all needed components into an already existing Galaxy instance. This ASaiM Docker instance is customized with a variety of selected tools, workflows, interactive tours and data that have been added as additional layers on top of the generic Galaxy Docker instance. The containerization keeps the deployment task to a minimum. The selected Galaxy tools are automatically installed from the Galaxy ToolShed [13] (https://toolshed.g2.bx.psu.edu) using the Galaxy API BioBlend [14] and the installation of the tools and their dependencies are automatically resolved using packages available through Bioconda (https://bioconda.github.io). To populate ASaiM with the selected tools, we migrated then 12 tools/suites of tools and their dependencies to Bioconda (*e.g.* HUMAnN2), integrated 16 suites (>100 tools) into Galaxy (*e.g.* HUMAn2 or QIIME with its approximately forty tools) and updated the already available ones (Table 1).

Tools for microbiota data analyses

The tools integrated in ASaiM can be seen in Table 1. They are expertly selected for their relevance with regard to microbiota studies, such as Mothur [3], QIIME [2], MetaPhlAn2 [15], HUMAnN2 [16] or tools used in existing pipelines such as EBI Metagenomics' one. We also added general tools used in sequence analysis such as quality control, mapping or similarity search tools.

111

112 **Table 1:** Available tools in ASaiM

Section	Subsection	Tools
File and meta tools	Data retrieval	EBISearch, ENASearch [17], SRA Tools
	Text manipulation	Tools from Galaxy ToolShed
	Sequence file manipulation	Tools from Galaxy ToolShed
	BAM/SAM file manipulation	SAM tools [18–20]
	BIOM file manipulation	BIOM-Format tools [21]
Genomics tools	Quality control	FastQC, PRINSEQ [22], Trim Galore!, Trimmomatic [23], MultiQC [24]
	Clustering	CD-Hit [25], Format CD-HIT outputs
	Sorting and prediction	SortMeRNA [26], FragGeneScan [27]
	Mapping	BWA [28,29], Bowtie [30]
	Similarity search	NCBI Blast+ [31,32], Diamond [33]
	Alignment	HMMER3
Microbiota dedicated tools	Metagenomics data manipulation	VSEARCH [10], Nonpareil [34]
	Assembly	MEGAHIT [35], metaSPAdes [36], metaQUAST [37], VALET
	Metataxonomic sequence analysis	Mothur [3], QIIME [2]
	Taxonomy assignation on WGS sequences	MetaPhlAn2 [15], Format MetaPhlan2, Kraken [9]
	Metabolism assignation	HUMAnN2 [16], Group HUMAnN2 to GO slim terms, Compare HUMAnN2 outputs, PICRUST [38], InterProScan
	Combination of functional and taxonomic results	Combine MetaPhlAn2 and HUMAnN2 outputs
	Visualization	Export2graphlan, GraPhlAn [39], KRONA [40]

113 This table presents the tools, organized in section and subsections to help users. A more detailed

114 table of the available tools and some documentation can be found in the online documentation

(http://asaim.readthedocs.io/en/latest/tools/)

An effort in development was made to integrate these tools into Conda and the Galaxy environment (> 100 tools integrated), with the help and support of the Galaxy community. We also developed two new tools to search and get data from EBI Metagenomics and ENA databases (EBISearch and ENASearch [17]) and a tool to group HUMAnN2 outputs into Gene Ontology Slim Terms. Tools inside ASaiM are organized to make them findable and documented (http://asaim.readthedocs.io/en/latest/tools/).

Diverse source of data

An easy way to upload user-data into ASaiM is provided by a web-interface or more sophisticated via FTP or SFTP. Moreover, we added specialised tools that can interact with external databases like NCBI, ENA or EBI Metagenomics to query them and download data into the ASaiM environment.

Visualization of the data

An analysis often ends with summarizing figures that conclude and represent the findings. ASaiM includes standard interactive plotting tools to draw bar charts and scatter plots from all kinds of tabular data. Phinch visualization is also included to interactively visualize and explore any BIOM file, and generate different types of ready-to-publish figures. We also integrated two other tools to explore and represent the community structure: KRONA [40] and GraPhlAn. Moreover, as in any Galaxy instance, other visualizations are included such as Phyloviz for phylogenetic trees or the genome browser Trackster for visualizing SAM/BAM, BED, GFF/GTF, WIG, bigWig, bigBed, bedGraph, and VCF datasets.

Workflows

Each tool can be used separately in an explorative manner or multiple tools can be orchestrated inside workflows passing raw data to information extraction and visualization.

To assist in microbiota analyses, several default workflows are proposed and documented (tools and their default parameters) in ASaiM. These workflows can be used as is, customized either on the fly to tune the parameters or globally to change the tools, their order and their default parameters, or even used as subworkflows.

Analysis of raw metagenomic or metatranscriptomic shotgun data

The workflow quickly produces, from raw metagenomic or metatranscriptomic shotgun data, accurate and precise taxonomic assignations, wide extended functional results and taxonomically related metabolism information (Figure 1). This workflow consists of i) processing with quality control/trimming (FastQC and Trim Galore!) and dereplication (VSearch [10]; ii) taxonomic analyses with assignation (MetaPhlAn2 [15]) and visualization (KRONA , GraPhlAn); iii) functional analyses with metabolic assignation and pathway reconstruction (HUMAnN2 [16]); iv) functional and taxonomic combination with developed tools combining HUMAnN2 and MetaPhlAn2 outputs.

This workflow has been tested on two mock metagenomic datasets with controlled communities (Supplementary material). We have compared the extracted taxonomic and functional information to such information extracted with the EBI metagenomics' pipeline and to the expectations from the mock datasets. With ASaiM, we generate more accurate and precise data for taxonomic analyses (Figure 2) and we can access information at the level of the species. More functional information (*e.g.* gene families, gene ontologies, pathways) are also extracted with ASaiM compared to the ones available on EBI metagenomics. With this workflow, we can go one step further and investigate which taxons are involved in a specific pathway or a gene family (*e.g.* involved species and their relative involvement in different step of fatty acid biosynthesis pathways, Figure 3).

For the tests, ASaiM was deployed on a computer with Debian GNU/Linux System, 8 cores Intel(R) Xeon(R) at 2.40 GHz and 32 Go of RAM. The workflow processed the 1,225,169 and 1,386,198 454 GS FLX Titanium reads of each datasets, with a stable memory usage, in 4h44 and 5h22 respectively (Supplementary material). The execution time is logarithmically

linked to the input data size. With this workflow, it is then easy and quick to process raw microbiota data and extract diverse useful information.

Assembly of metagenomics data

Microbiota data usually come with quite short reads. To reconstruct genomes or to get longer sequences for further analysis, microbiota sequences have to be assembled with dedicated metagenome assemblers. To help in this task, two workflows have been developed in ASaiM, each one using one of the well-performing assemblers [41–47]: MEGAHIT [35] and MetaSPAdes [36]. Both workflows consists of: 1) processing with quality control/trimming (FastQC and Trim Galore!); ii) assembly with either MEGAHIT or MetaSPAdes; iii) estimation of the assembly quality statistics with MetaQUAST [37]; iv) identification of potential assembly error signature with VALET; v) determination of percentage of unmapped reads with Bowtie2 [30] combined with MultiQC [24] to aggregate the results.

Analysis of metataxonomic data

To analyze amplicon or ITS data, the Mothur and QIIME tool suites are available to ASaiM. We integrated the workflows described in tutorials of Mothur and QIIME, as example of metataxonomic data analyses as well as support for the training material.

Running as in EBI metagenomics

As the tools used in the EBI Metagenomics pipeline are also available in ASaiM, we integrate them in a workflow with the same steps as the EBI Metagenomics pipeline. Analyses made in EBI Metagenomics website can be then reproduced locally, without having to wait for availability of EBI Metagenomics or to upload any data on EBI Metagenomics. However the parameters must be defined by the user as we can not find them on EBI Metagenomics documentation. In ASaiM, the entire provenance and every parameters are tracked to guarantee the reproducibility.

Documentation and training

A tool or software is easier to use if it is well documented. Hence extensive documentation helps the users to be familiar with the tool and also prevents mis-usage. For ASaiM, we developed an extensive online documentation (http://asaim.readthedocs.io), mainly to explain how to use it, how to deploy it, which tools are integrated with small documentation about these tools, which workflows are integrated and how to use them.

In addition to this online documentation, Galaxy Interactive Tours are included inside the Galaxy instance, which guide users through an entire analysis in an interactive (step-by-step) way. We developed few tours dedicated specifically to microbiota analyses and ASaiM workflows, to complement developed tutorials and trainings. Several step-by-step tutorials explain different microbiota analyses and ASaiM workflows with toy datasets. Hosted in the Galaxy Training Network (GTN) GitHub repository (https://github.com/galaxyproject/training-material), the tutorials are available online at http://training.galaxyproject.org/topics/metagenomics and also directly from ASaiM and its documentation for self-training. They have been used during several workshops on metagenomics data analysis. In parallel, ASaiM has been used in training courses to understand and use the EBI Metagenomics workflow in a reproducible way for teaching undergrads, and as foundation in a citizen science project (Beer DeCoded [48]).

Installation and running ASaiM

Running the containerized ASaiM simply requires to install Docker and to start the ASaiM image with:

```
$ docker run -d -p 8080:80 quay.io/bebatut/asaim-framework:latest
```

As Galaxy, ASaiM is production-ready and can be configured to use external accessible computer clusters or cloud environments. It is also possible and easy to install all or only a subset of tools of the ASaiM framework on existing Galaxy instances, as we did on the Freiburg Galaxy instance. More details about the installation and the use of ASaiM are

available on the online documentation (http://asaim.readthedocs.io/en/latest/installation.html).

Conclusion

ASaiM provides a powerful framework to easily and quickly analyze microbiota data in a reproducible, accessible and transparent way. Built on a Galaxy instance wrapped in a Docker image, ASaiM can be easily deployed with its extensive set of tools and their dependencies. These tools are complemented with a set of predefined and tested workflows to address the main microbiota questions (assembly, community structure and functions). All these tools and workflows are extensively documented online (http://asaim.readthedocs.io) and supported by Interactive Tours and tutorials.

With this complete infrastructure, ASaiM offers a sophisticated environment for microbiota analyses to any scientists, while promoting transparency, sharing and reproducibility.

Methods

For the tests, ASaiM was deployed on a computer with Debian GNU/Linux System, 8 cores Intel(R) Xeon(R) at 2.40 GHz and 32 Go of RAM. The workflow has been run on two mock community samples of Human Microbiome Project (HMP), containing a genomic mixture of 22 known microbial strains. The details of comparison analyses are described in the Supplementary Material.

Availability of supporting source code and requirements

- Project name: ASaiM
- Project home page: https://github.com/ASaiM/framework
- Operating system(s): Platform independent
- Other requirements: Docker
- License: Apache 2

All tools described herein are available in the Galaxy Toolshed (https://toolshed.g2.bx.psu.edu). The Dockerfile to automatically install deploy ASaiM is provided in the GitHub repository and a pre-built Docker image is available at https://quay.io/repository/bebatut/asaim-framework.

Declarations

Competing interests

The author(s) declare that they have no competing interests.

Funding

The Auvergne Regional Council and the European Regional Development Fund have supported this work.

Authors' contributions

BB, KG, CD, SH, JFB, EP, PP contributed equally to the conceptualization, to the methodology and to the writing process. JFB, PP contributed equally to the funding acquisition. BB, KG, SH contributed equally to the software development and BB, KG, CD and JFB to the validation.

Acknowledgements

The authors would like to thank EA 4678 CIDAM, UR 454 INRA, M2iSH, LIMOS, AuBi, Mésocentre, de.NBI for their involvement in this project, as well as Réjane Beugnot, Thomas Eymard, David Parsons and Björn Grüning for their help.

References

1. Ladoukakis E, Kolisis FN, Chatziioannou AA. Integrative workflows for metagenomic

analysis. Front Cell Dev Biol. 2014;2:70.

2. Kuczynski J, Stombaugh J, Walters WA, González A, Gregory Caporaso J, Knight R. Using QIIME to Analyze 16S rRNA Gene Sequences from Microbial Communities. Current Protocols in Microbiology. 2012. p. 1E.5.1–1E.5.20.

3. Schloss PD, Westcott SL, Ryabin T, Hall JR, Hartmann M, Hollister EB, et al. Introducing mothur: open-source, platform-independent, community-supported software for describing and comparing microbial communities. Appl. Environ. Microbiol. 2009;75:7537–41.

4. Nekrutenko A, Taylor J. Next-generation sequencing data interpretation: enhancing reproducibility and accessibility. Nat. Rev. Genet. 2012;13:667–72.

5. Meyer F, Paarmann D, D'Souza M, Olson R, Glass EM, Kubal M, et al. The metagenomics RAST server – a public resource for the automatic phylogenetic and functional analysis of metagenomes. BMC Bioinformatics. 2008;9:386.

6. Hunter S, Corbett M, Denise H, Fraser M, Gonzalez-Beltran A, Hunter C, et al. EBI metagenomics--a new resource for the analysis and archiving of metagenomic data. Nucleic Acids Res. 2014;42:D600–6.

7. Goecks J, Nekrutenko A, Taylor J, Galaxy Team. Galaxy: a comprehensive approach for supporting accessible, reproducible, and transparent computational research in the life sciences. Genome Biol. 2010;11:R86.

8. Afgan E, Baker D, van den Beek M, Blankenberg D, Bouvier D, Čech M, et al. The Galaxy platform for accessible, reproducible and collaborative biomedical analyses: 2016 update. Nucleic Acids Res. 2016;44:W3–10.

9. Wood DE, Salzberg SL. Kraken: ultrafast metagenomic sequence classification using exact alignments. Genome Biol. 2014;15:R46.

10. Rognes T, Flouri T, Nichols B, Quince C, Mahé F. VSEARCH: a versatile open source tool for metagenomics. PeerJ. 2016;4:e2584.

11. Kosakovsky Pond S, Wadhawan S, Chiaromonte F, Ananda G, Chung W-Y, Taylor J, et al. Windshield splatter analysis with the Galaxy metagenomic pipeline. Genome Res. 2009;19:2144–53.

12. Grüning BA, Fallmann J, Yusuf D, Will S, Erxleben A, Eggenhofer F, et al. The RNA workbench: best practices for RNA and high-throughput sequencing bioinformatics in Galaxy. Nucleic Acids Res. [Internet]. 2017; Available from: http://dx.doi.org/10.1093/nar/gkx409

13. Blankenberg D, Von Kuster G, Bouvier E, Baker D, Afgan E, Stoler N, et al. Dissemination of scientific software with Galaxy ToolShed. Genome Biol. 2014;15:403.

14. Sloggett C, Goonasekera N, Afgan E. BioBlend: automating pipeline analyses within Galaxy and CloudMan. Bioinformatics. 2013;29:1685–6.

15. Truong DT, Franzosa EA, Tickle TL, Scholz M, Weingart G, Pasolli E, et al. MetaPhlAn2 for enhanced metagenomic taxonomic profiling. Nat. Methods. 2015;12:902–3.

16. Abubucker S, Segata N, Goll J, Schubert AM, Izard J, Cantarel BL, et al. Metabolic reconstruction for metagenomic data and its application to the human microbiome. PLoS Comput. Biol. 2012;8:e1002358.

17. Batut B, Grüning B. ENASearch: A Python library for interacting with ENA's API. The Journal of Open Source Software. 2017;2:418.

18. Li H. A statistical framework for SNP calling, mutation discovery, association mapping and population genetical parameter estimation from sequencing data. Bioinformatics. 2011;27:2987–93.

19. Li H. Improving SNP discovery by base alignment quality. Bioinformatics. 2011;27:1157–8.

20. Li H, Handsaker B, Wysoker A, Fennell T, Ruan J, Homer N, et al. The Sequence Alignment/Map format and SAMtools. Bioinformatics. 2009;25:2078–9.

21. McDonald D, Clemente JC, Kuczynski J, Rideout JR, Stombaugh J, Wendel D, et al. The Biological Observation Matrix (BIOM) format or: how I learned to stop worrying and love the ome-ome. Gigascience. 2012;1:7.

22. Schmieder R, Edwards R. Quality control and preprocessing of metagenomic datasets. Bioinformatics. 2011;27:863–4.

23. Bolger AM, Lohse M, Usadel B. Trimmomatic: a flexible trimmer for Illumina sequence data. Bioinformatics. 2014;30:2114–20.

24. Ewels P, Magnusson M, Lundin S, Käller M. MultiQC: summarize analysis results for multiple tools and samples in a single report. Bioinformatics. 2016;32:3047–8.

25. Fu L, Niu B, Zhu Z, Wu S, Li W. CD-HIT: accelerated for clustering the next-generation sequencing data. Bioinformatics. 2012;28:3150–2.

26. Kopylova E, Noé L, Touzet H. SortMeRNA: fast and accurate filtering of ribosomal RNAs in metatranscriptomic data. Bioinformatics. 2012;28:3211–7.

27. Rho M, Tang H, Ye Y. FragGeneScan: predicting genes in short and error-prone reads. Nucleic Acids Res. 2010;38:e191–e191.

28. Li H, Durbin R. Fast and accurate short read alignment with Burrows-Wheeler transform. Bioinformatics. 2009;25:1754–60.

29. Li H, Durbin R. Fast and accurate long-read alignment with Burrows-Wheeler transform. Bioinformatics. 2010;26:589–95.

30. Press AR. Ultrafast and Memory-Efficient Alignment of Short DNA Sequences to the Human Genome. CreateSpace; 2015.

31. Camacho C, Coulouris G, Avagyan V, Ma N, Papadopoulos J, Bealer K, et al. BLAST+: architecture and applications. BMC Bioinformatics. 2009;10:421.

32. Cock PJA, Chilton JM, Grüning B, Johnson JE, Soranzo N. NCBI BLAST+ integrated into Galaxy. Gigascience. 2015;4:39.

33. Buchfink B, Xie C, Huson DH. Fast and sensitive protein alignment using DIAMOND. Nat. Methods. 2015;12:59–60.

34. Rodriguez-R LM, Konstantinidis KT. Nonpareil: a redundancy-based approach to assess the level of coverage in metagenomic datasets. Bioinformatics. 2014;30:629–35.

35. Li D, Luo R, Liu C-M, Leung C-M, Ting H-F, Sadakane K, et al. MEGAHIT v1.0: A fast

and scalable metagenome assembler driven by advanced methodologies and community practices. Methods. 2016;102:3–11.

36. Nurk S, Meleshko D, Korobeynikov A, Pevzner PA. metaSPAdes: a new versatile metagenomic assembler. Genome Res. 2017;27:824–34.

37. Mikheenko A, Saveliev V, Gurevich A. MetaQUAST: evaluation of metagenome assemblies. Bioinformatics. 2016;32:1088–90.

38. Langille MGI, Zaneveld J, Caporaso JG, McDonald D, Knights D, Reyes JA, et al. Predictive functional profiling of microbial communities using 16S rRNA marker gene sequences. Nat. Biotechnol. 2013;31:814–21.

39. Asnicar F, Weingart G, Tickle TL, Huttenhower C, Segata N. Compact graphical representation of phylogenetic data and metadata with GraPhlAn. PeerJ. 2015;3:e1029.

40. Ondov BD, Bergman NH, Phillippy AM. Interactive metagenomic visualization in a Web browser. BMC Bioinformatics. 2011;12:385.

41. Awad S, Irber L, Titus Brown C. Evaluating Metagenome Assembly on a Simple Defined Community with Many Strain Variants [Internet]. 2017. Available from: http://dx.doi.org/10.1101/155358

42. Greenwald WW, Klitgord N, Seguritan V, Yooseph S, Venter JC, Garner C, et al. Utilization of defined microbial communities enables effective evaluation of meta-genomic assemblies. BMC Genomics. 2017;18:296.

43. Olson ND, Treangen TJ, Hill CM, Cepeda-Espinoza V, Ghurye J, Koren S, et al. Metagenomic assembly through the lens of validation: recent advances in assessing and improving the quality of genomes assembled from metagenomes. Brief. Bioinform. [Internet]. 2017; Available from: http://dx.doi.org/10.1093/bib/bbx098

44. Quince C, Walker AW, Simpson JT, Loman NJ, Segata N. Shotgun metagenomics, from sampling to analysis. Nat. Biotechnol. 2017;35:833–44.

45. Sczyrba A, Hofmann P, Belmann P, Koslicki D, Janssen S, Dröge J, et al. Critical Assessment of Metagenome Interpretation-a benchmark of metagenomics software. Nat. Methods. 2017;14:1063–71.

46. van der Walt AJ, Van Goethem MW, Ramond J-B, Makhalanyane TP, Reva O, Cowan DA. Assembling Metagenomes, One Community At A Time [Internet]. 2017. Available from: http://dx.doi.org/10.1101/120154

47. Vollmers J, Wiegand S, Kaster A-K. Comparing and Evaluating Metagenome Assembly Tools from a Microbiologist’s Perspective - Not Only Size Matters! PLoS One. 2017;12:e0169662.

48. Sobel J, Henry L, Rotman N, Rando G. BeerDeCoded: the open beer metagenome project. F1000Res. 2017;6:1676.

Figure 1: Main ASaiM workflow to analyze raw sequences.

This workflow takes as input a dataset of raw shotgun sequences (in FastQ format) from microbiota, preprocess it (yellow boxes), extracts taxonomic (red boxes) and functional (purple boxes) assignations and combines them (green boxes).

Image available under CC-BY license (https://doi.org/10.6084/m9.figshare.5371396.v3)

Figure 2: Comparisons of the community structure for SRR072233.

This figure compares the community structure between the expectations (mapping of the sequences on the expected genomes), data found on EBI Metagenomics database (extracted with the EBI Metagenomics pipeline) and the results of the main ASaiM workflow (Figure 1).

Figure 3: Example of an investigation of the relation between community structure and functions.

The involved species and their relative involvement in fatty acid biosynthesis pathways have been extracted with ASaiM workflow (Figure 1) for SRR072233

Figure 1: Main ASaiM workflow to analyze raw sequences.

Click here to download Figure main_workflow.pdf

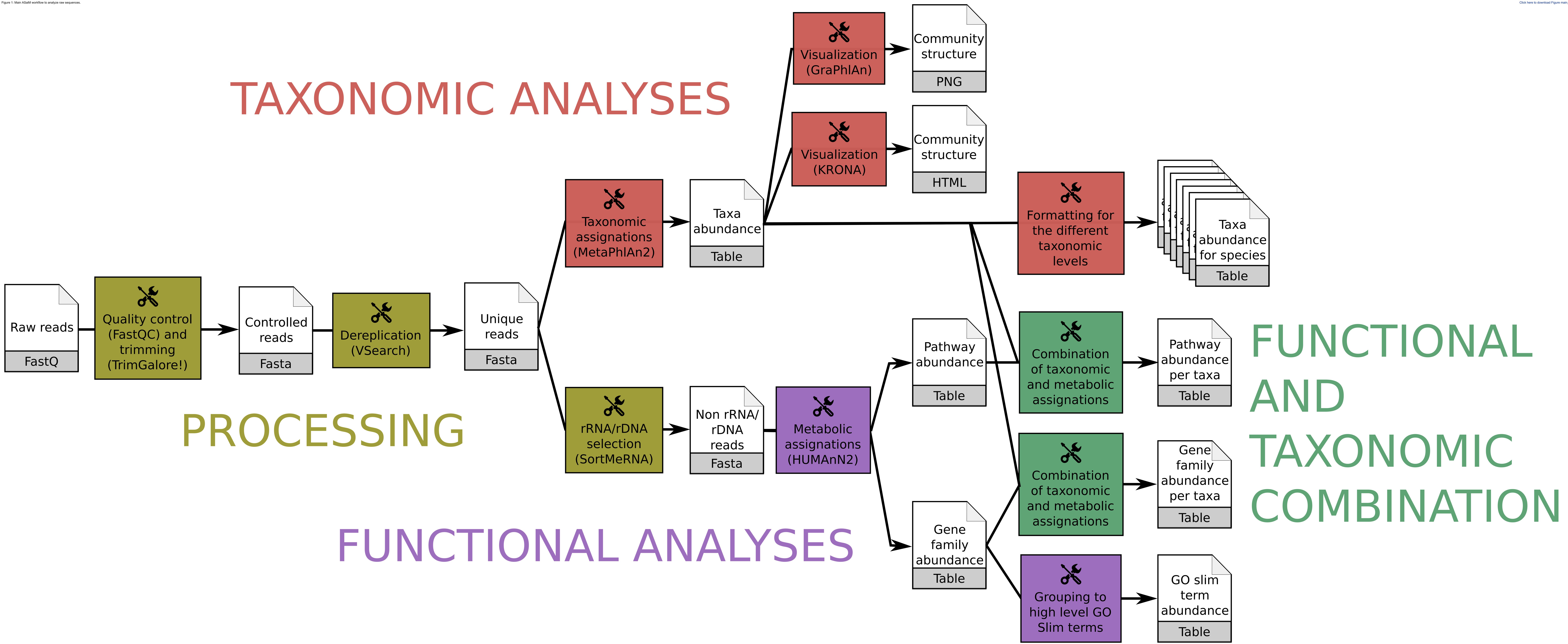

Figure 2: Comparisons of the community structure for SRR072233

Click here to download Figure hmp_taxonomic_results.pdf

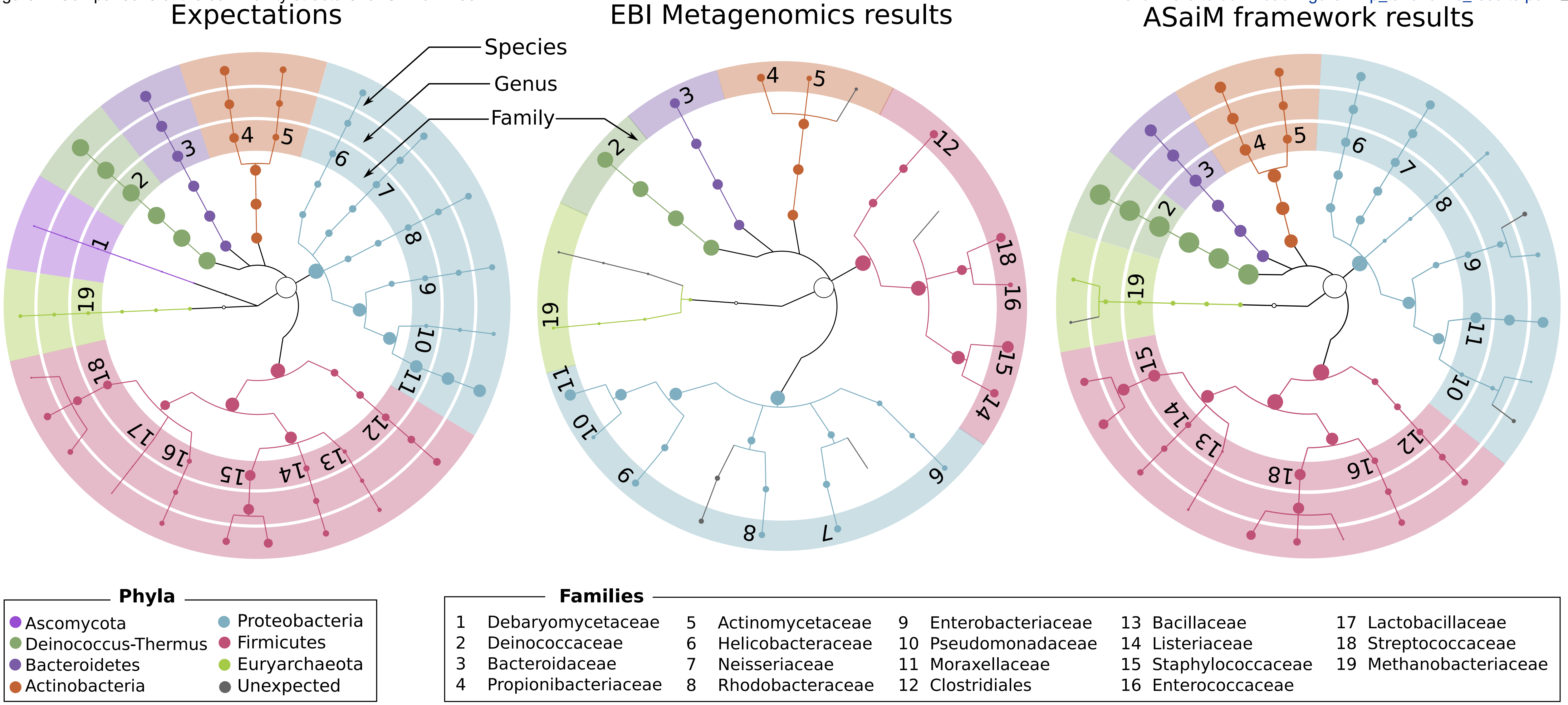

Figure 3: Example of an investigation of the relation between community structure and functions

Click here to download Figure hmp_taxonomically_related_functional_results.pdf

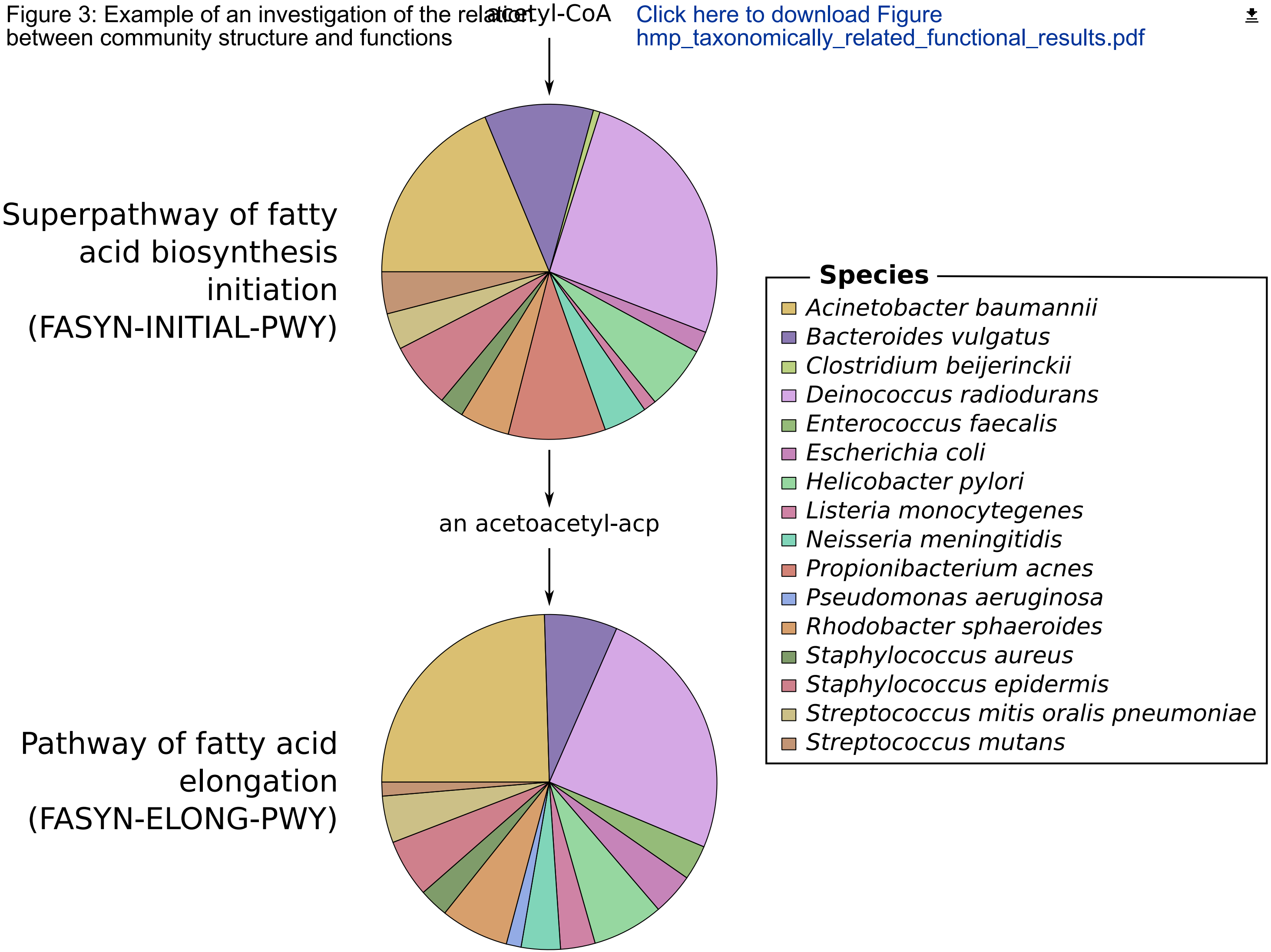

Click here to access/download
Supplementary Material
sup_mat_1.pdf
