# Supplementary material for: ASaiM: a Galaxy-based framework to analyze microbiota data
Source: Gigascience. 2018 May 15;7(6):giy057. doi: 10.1093/gigascience/giy057 (PMC6007547; doi:10.1093/gigascience/giy057)
Supplement: GIGA-D-17-00230_Revision_2.pdf [file giy057_giga-d-17-00230_revision_2.pdf]

GigaScience

ASaiM: a Galaxy-based framework to analyze microbiota data

--Manuscript Draft--

**Manuscript Number:**	GIGA-D-17-00230R2
**Full Title:**	ASaiM: a Galaxy-based framework to analyze microbiota data
**Article Type:**	Technical Note
**Funding Information:**	Conseil Régional d'Auvergne — Dr Bérénice Batut<br>European Regional Development Fund — Not applicable
**Abstract:**	New generation of sequencing platforms coupled to numerous bioinformatics tools has led to rapid technological progress in metagenomics and metatranscriptomics to investigate complex microorganism communities. Nevertheless, a combination of different bioinformatic tools remains necessary to draw conclusions out of microbiota studies. Modular and user-friendly tools would greatly improve such studies.<br><br>We therefore developed ASaiM, an Open-Source Galaxy-based framework dedicated to microbiota data analyses. ASaiM provides an extensive collection of tools to assemble, extract, explore and visualize microbiota information from raw metataxonomic, metagenomic or metatranscriptomic sequences. To guide the analyses, several customizable workflows are included and are supported by tutorials and Galaxy interactive tours, which guide users through the analyses step by step. ASaiM is implemented as Galaxy Docker flavour. It is scalable to thousands of datasets, but also can be used on a normal PC. The associated source code is available under Apache 2 license at https://github.com/ASaiM/framework and documentation can be found online (http://asaim.readthedocs.io)<br><br>Based on the Galaxy framework, ASaiM offers a sophisticated environment with a variety of tools, workflows, documentation and training to scientists working on complex microorganism communities. It makes analysis and exploration analyses of microbiota data easy, quick, transparent, reproducible and shareable.
**Corresponding Author:**	Bérénice Batut, Ph.D.<br>University of Freiburg<br>Freiburg, GERMANY
**Corresponding Author Secondary Information:**	
**Corresponding Author's Institution:**	University of Freiburg
**Corresponding Author's Secondary Institution:**	
**First Author:**	Bérénice Batut, Ph.D.
**First Author Secondary Information:**	
**Order of Authors:**	Bérénice Batut, Ph.D.<br>Kévin Gravouil<br>Clémence Defois<br>Saskia Hiltemann<br>Jean-François Brugère<br>Eric Peyretaillade<br>Pierre Peyret
**Order of Authors Secondary Information:**	
**Response to Reviewers:**	We thanked the reviewers for their new reviews. We tried to answer all of them, but we are not sure about the point raised by the Reviewer #2.

Powered by Editorial Manager® and ProduXion Manager® from Aries Systems Corporation

Reviewer #2

Please make sure to double check the Figures before publication. Figure 3 seems to have its title overlap with some other text.

We are sorry for the inconvenience, but can not see this locally. We will wait for the proofs and make sure this does not end up in the final PDF.

Reviewer #3

While this revised version of the manuscript improves on the previously submitted one, this Reviewer believes that a few points still need to be addressed:

1. While this Reviewer agrees that ASaiM allows users to overcome "the difficulty to find, configure, use and combine the dedicated bioinformatics tools", it is still true that "to extract useful information, a sequenced microbiota sample has to be processed by sophisticated workflows with numerous successive bioinformatics steps", that "Each step may require execution of several tools or software", that "[tools] may require extensive computational resources (memory, disk space)", and, finally, that "selecting the best tools, configuring them to use the correct parameters and appropriate computational resources and combining them together in an analysis chain is a complex and error-prone process.". This Reviewer suggests reframing the manuscript either stressing on ASaiM's strengths compared to state-of-the-art tools (that is, in this Reviewer's opinion, saving the users from the hassle of installing all the pieces of software, and implementing a few well-known pipelines
into Galaxy, an universally-acknowledged user-friendly platform), or clarifying, how ASaiM solves the issues raised above (that is, mostly, how i) ASaiM diminishes the memory/space requirements, ii) helps users in designing novel meaningful pipelines using the >100 tools included, and iii) helps users in setting meaningful parameters/resources in each of these steps). Following on this comment, the limitation of both QIIME and Mothur that is: "Designed for amplicon data, both QIIME and Mothur can not be directly applied to shotgun metagenomics data." is still not addressed by their ASaiM implementation and should, in this Reviewer's opinion, be removed.

The authors understand the first point of the reviewer and tried to clarify in the manuscript how ASaiM solves the raised issues. In the introduction of the workflow section, the authors add sentences to show how ASaiM helps users in setting meaningful parameters for tools and also in designing novel meaningful workflows. In the conclusion, the authors added few words to insist on the automated hassle of tool installation. Galaxy via ASaiM will not address the memory limitations, but Galaxy will efficiently schedule jobs as well as manage the memory usage. This information has also been added in the manuscript.

For Mothur and QIIME related question, ASaiM offered tools and workflows for amplicon or metataxomic data using QIIME and Mothur, but also for shotgun metagenomics data (using MetaPhlAn2 and HUMAnN2). ASaiM is not only then focused on amplicon data as QIIME and Mothur are.

2. In this Reviewer's opinion, the comparison between ASaiM and the EBI pipeline is irrelevant, since they use different tools (and it rather seems a comparison between these tools). If the authors cannot provide a fair comparison, this paragraph could, in this Reviewer's opinion, be removed without loss of information.

The idea of the comparison between ASaiM and EBI metagenomics was to demonstrate the limitation of one approach against the other on the analysis of shotgun metagenomic data. We are not benchmarking the tools, just trying to illustrate the possibilities of ASaiM.

3. This Reviewer agrees that time and other computational requirements greatly depend on the input data, and thus suggests carrying on a benchmarking of all the implemented pipelines using multiple datasets, with different numbers of reads (many, as those belonging to the Hunan Metagenome Project, are freely available). This will

	help users in "selecting the best tools, configuring them to use the correct parameters and appropriate computational resources", and give them more useful information than that which can be extracted by only two datasets.<br><br>Such general benchmarking would be interesting and we are working currently together with other researchers to establish a general benchmarking, as mentioned by the reviewer. Using the information of the benchmarking, we would like to build an environment where users could be helped with tool selection and configuration and jobs/workflows automatically tweaks in Galaxy. We feel that this is out of scope for the manuscript but we are working on this as a more general framework, probably not only for metagenomics.<br><br>4. Minor comment: since there is no agreement yet on some of the terms used, it may be worth using 16S rRNA marker gene sequencing or amplicon sequencing, instead of metataxonomic, and whole metagenomic shotgun sequencing, instead of simply metagenomics.<br><br>The authors agree that there is a confusion in vocabulary used in the field of microbial community analysis. Marchesi & Ravel in their 2015 paper (Microbiome: (https://microbiomejournal.biomedcentral.com/articles/10.1186/s40168-015-0094-5) tried to establish a consensus vocabulary. To support this initiative, the authors decided to use the terms and definitions given in this paper. We hope this makes our paper more readable in the long run and supports the initiative started by Marchesi & Ravel.
**Additional Information:**	
**Question**	**Response**
Are you submitting this manuscript to a special series or article collection?	No
**Experimental design and statistics**<br><br>Full details of the experimental design and statistical methods used should be given in the Methods section, as detailed in our Minimum Standards Reporting Checklist. Information essential to interpreting the data presented should be made available in the figure legends.<br><br>Have you included all the information requested in your manuscript?	Yes
**Resources**<br><br>A description of all resources used, including antibodies, cell lines, animals and software tools, with enough information to allow them to be uniquely identified, should be included in the Methods section. Authors are strongly encouraged to cite Research Resource Identifiers (RRIDs) for antibodies, model organisms and tools, where possible.<br><br>Have you included the information requested as detailed in our Minimum Standards Reporting Checklist?	Yes

Powered by Editorial Manager® and ProduXion Manager® from Aries Systems Corporation

**Availability of data and materials**<br><br>All datasets and code on which the conclusions of the paper rely must be either included in your submission or deposited in publicly available repositories (where available and ethically appropriate), referencing such data using a unique identifier in the references and in the “Availability of Data and Materials” section of your manuscript.<br><br>Have you have met the above requirement as detailed in our Minimum Standards Reporting Checklist?	Yes

Powered by Editorial Manager® and ProduXion Manager® from Aries Systems Corporation

Manuscript Click here to download Manuscript ASaiM_GigaSciences.docx

ASaiM: a Galaxy-based framework to analyze microbiota data

Bérénice Batut[1,*], Kévin Gravouil[2,3,4], Clémence Defois[2], Saskia Hiltemann[5], Jean-François Brugère[2], Eric Peyretaillade[2] and Pierre Peyret[2,*]

Author affiliations

[1]Bioinformatics Group, Department of Computer Science, University of Freiburg, Germany

[2]Université Clermont Auvergne, INRA, MEDIS, F-63000 Clermont-Ferrand, France

[3]Université Clermont Auvergne, CNRS, LMGE, F-63000 Clermont–Ferrand, France

[4]Université Clermont Auvergne, CNRS, LIMOS, F-63000 Clermont–Ferrand, France

[5]Department of Bioinformatics, Erasmus University Medical Center, Rotterdam, 3015 CE, Netherlands

Correspondence should be addressed to B.B. (berenice.batut@gmail.com, ORCID: 0000-0001-9852-1987) and P.P. (pierre.peyret@uca.fr, ORCID: 0000-0003-3114-0586)

Abstract

Background

New generations of sequencing platforms coupled to numerous bioinformatics tools has led to rapid technological progress in metagenomics and metatranscriptomics to investigate complex microorganism communities. Nevertheless, a combination of different bioinformatic tools remains necessary to draw conclusions out of microbiota studies. Modular and user-friendly tools would greatly improve such studies.

Findings

We therefore developed ASaiM, an Open-Source Galaxy-based framework dedicated to microbiota data analyses. ASaiM provides an extensive collection of tools to assemble, extract, explore and visualize microbiota information from raw metataxonomic, metagenomic or metatranscriptomic sequences. To guide the analyses, several customizable workflows are included and are supported by tutorials and Galaxy interactive tours, which guide users through the analyses step by step. ASaiM is implemented as a Galaxy Docker flavour. It is scalable to thousands of datasets, but also can be used on a normal PC. The associated source code is available under Apache 2 license at https://github.com/ASaiM/framework and documentation can be found online (http://asaim.readthedocs.io)

Conclusions

Based on the Galaxy framework, ASaiM offers a sophisticated environment with a variety of tools, workflows, documentation and training to scientists working on complex microorganism communities. It makes analysis and exploration analyses of microbiota data easy, quick, transparent, reproducible and shareable.

Keywords

Metagenomics, Metataxonomics, User-friendly, Galaxy, Docker, Microbiota, Training.

Findings

Background

The study of microbiota and microbial communities has been facilitated by the evolution of sequencing techniques and the development of metataxonomics, metagenomics and metatranscriptomics. These techniques are giving insight into taxonomic profiles and genomic components of microbial communities. However, meta'omic data exploitation is not trivial due to the large amount of data, their complexity, the incompleteness of reference databases, the difficulty to find, configure, use and combine the dedicated bioinformatics tools, etc. Hence, to extract useful information, a sequenced microbiota sample has to be processed by sophisticated workflows with numerous successive bioinformatics steps [1]. Each step may require execution of several tools or software. For example, to extract taxonomic information with the widely used QIIME [2] or Mothur [3], at least 10 different tools with at least 4 parameters each are needed. Designed for amplicon data, both QIIME and Mothur can not be directly applied to shotgun metagenomics data. In addition, the tools can be complex to use; they are command-line tools and may require extensive computational resources (memory, disk space). In this context, selecting the best tools, configuring them to use the correct parameters and appropriate computational resources and combining them together in an analysis chain is a complex and error-prone process. These issues and the involved complexity are prohibiting scientists from participating in the analysis of their own data. Furthermore, bioinformatics tools are often manually executed and/or patched together with custom scripts. These practices raise doubts about a science gold standard: reproducibility [3,4]. Web services and automated pipelines such as MG-RAST [5] and EBI metagenomics [6] offer solutions to the accessibility issue. However, these web services

work as a black box and are lacking in transparency, flexibility and even reproducibility as the version and parameters of the tools are not always available. Alternative approaches to improve accessibility, modularity and reproducibility can be found in open-source workflow systems such as Galaxy [6–8]. Galaxy is a lightweight environment providing a web-based, intuitive and accessible user interface to command-line tools, while automatically managing computation and transparently managing data provenance and workflow scheduling [6–8]. More than 5,500 tools can be used inside any Galaxy environment. For example, the main Galaxy server (http://usegalaxy.org) integrates many genomic tools, and the few integrated metagenomics tools such as Kraken [9] or VSearch [10] have been showcased in the published windshield splatter analysis [11]. The tools can also be selected and combined to build Galaxy flavors focusing on specific type of analysis, *e.g.* the Galaxy RNA workbench [12] or the specialized Galaxy server of the Huttenhower lab (http://huttenhower.sph.harvard.edu/galaxy). However, none of these solutions are dedicated to microbiota data analysis in general, and with the community-standard tools.

In this context, we developed ASaiM (Auvergne Sequence analysis of intestinal Microbiota, RRID:SCR_015878), an Open-Source opinionated Galaxy-based framework. It integrates more than 100 tools and several workflows dedicated to microbiota analyses with an extensive documentation (http://asaim.readthedocs.io) and training support.

Goals of ASaiM

ASaiM is developed as a modular, accessible, redistributable, sharable and user-friendly framework for scientists working with microbiota data. This framework is unique in combining curated tools and workflows and providing easy access and support for scientists.

ASaiM is based on four pillars: 1) easy and stable dissemination via Galaxy, Docker and Conda, 2) a comprehensive set of microbiota related tools, 3) a set of predefined and tested workflows, and 4) extensive documentation and training to help scientists in their analyses.

A framework built on the shoulders of giants

The ASaiM framework is built on existing tools and infrastructures and combine all their forces to create an easily accessible and reproducible analysis platform.

ASaiM is implemented as a portable virtualized container based on the Galaxy framework [8]. Galaxy provides researchers with means to reproduce their own workflows analyses, rerun entire pipelines, or publish and share them with others. Based on Galaxy, ASaiM is scalable from single CPU installations to large multi-node high performance computing environments and manages efficiently job submission as well as memory consumption of the tools. Deployments can be achieved by using a pre-built ASaiM Docker image, which is based on the Galaxy Docker project (http://bgruening.github.io/docker-galaxy-stable). This ASaiM Docker flavour is customized with a variety of selected tools, workflows, interactive tours and data that have been added as additional layers on top of the generic Galaxy Docker instance. The containerization keeps the deployment task to a minimum. The selected Galaxy tools are automatically installed from the Galaxy ToolShed [13] (https://toolshed.g2.bx.psu.edu) using the Galaxy API BioBlend [14] and the installation of the tools and their dependencies are automatically resolved using packages available through Bioconda [15] (https://bioconda.github.io). To populate ASaiM with the selected microbiota tools, we migrated then 12 tools/suites of tools and their dependencies to Bioconda (*e.g.* HUMAnN2), integrated 16 suites (>100 tools) into Galaxy (*e.g.* HUMAn2 or QIIME with its approximately forty tools) and updated the already available ones (Table 1).

Tools for microbiota data analyses

The tools integrated in ASaiM can be seen in Table 1. They are expertly selected for their relevance with regard to microbiota studies, such as Mothur (mothur , RRID:SCR_011947)[3], QIIME (QIIME, RRID:SCR_008249)[2], MetaPhlAn2 (MetaPhlAn, RRID:SCR_004915)[16], HUMAnN2 [17] or tools used in existing pipelines such as EBI

Metagenomics' one. We also added general tools used in sequence analysis such as quality control, mapping or similarity search tools.

Table 1: Available tools in ASaiM

**Section**	**Subsection**	**Tools**
File and meta tools	Data retrieval	EBISearch, ENASearch [18], SRA Tools
	Text manipulation	Tools from Galaxy ToolShed
	Sequence file manipulation	Tools from Galaxy ToolShed
	BAM/SAM file manipulation	SAM tools [19–21]
	BIOM file manipulation	BIOM-Format tools [22]
Genomics tools	Quality control	FastQC, PRINSEQ [23], Trim Galore!, Trimmomatic [24], MultiQC [25]
	Clustering	CD-Hit [26], Format CD-HIT outputs
	Sorting and prediction	SortMeRNA [27], FragGeneScan [28]
	Mapping	BWA [29], Bowtie [30]
	Similarity search	NCBI Blast+ [31,32], Diamond [33]
	Alignment	HMMER3 [34]
Microbiota dedicated tools	Metagenomics data manipulation	VSEARCH [10], Nonpareil [35]
	Assembly	MEGAHIT [36], metaSPAdes [37], metaQUAST [38], VALET
	Metataxonomic sequence analysis	Mothur [3], QIIME [2]
	Taxonomy assignation on WGS sequences	MetaPhlAn2 [16], Format MetaPhlan2, Kraken [9]
	Metabolism assignation	HUMAnN2 [17], Group HUMAnN2 to GO slim terms, Compare HUMAnN2 outputs, PICRUST [39], InterProScan
	Combination of functional and taxonomic results	Combine MetaPhlAn2 and HUMAnN2 outputs

Visualization	Export2graphlan, GraPhlAn [40], KRONA [41]

This table presents the tools, organized in section and subsections to help users. A more detailed table of the available tools and some documentation can be found in the online documentation (http://asaim.readthedocs.io/en/latest/tools/)

An effort in development was made to integrate these tools into Conda and the Galaxy environment (> 100 tools integrated), with the help and support of the Galaxy community. We also developed two new tools to search and get data from EBI Metagenomics and ENA databases (EBISearch and ENASearch [18]) and a tool to group HUMAnN2 outputs into Gene Ontology Slim Terms. Tools inside ASaiM are documented (http://asaim.readthedocs.io/en/latest/tools/) and organized to make them findable.

Diverse source of data

An easy way to upload user-data into ASaiM is provided by a web-interface or more sophisticated via FTP or SFTP. Moreover, we added specialised tools that can interact with external databases like NCBI, ENA or EBI Metagenomics to query them and download data into the ASaiM environment.

Visualization of the data

An analysis often ends with summarizing figures that conclude and represent the findings. ASaiM includes standard interactive plotting tools to draw bar charts and scatter plots for all kinds of tabular data. Phinch visualization is also included to interactively visualize and explore any BIOM file, and generate different types of ready-to-publish figures. We also integrated two other tools to explore and represent the community structure: KRONA [41] and GraPhlAn. Moreover, as in any Galaxy instance, other visualizations are included such as Phyloviz for phylogenetic trees or the genome browser Trackster for visualizing SAM/BAM, BED, GFF/GTF, WIG, bigWig, bigBed, bedGraph, and VCF datasets.

Workflows

Each tool can be used separately in an explorative manner, the Galaxy tool form helping users in meaningful parameter settings. Tools can be also orchestrated inside workflows using the powerful Galaxy workflow manager. To assist in microbiota analyses, several workflows, including a few well-known pipelines, are offered and documented (tools and their default parameters) in ASaiM. These workflows can be used as is, customized either on the fly to tune the parameters or globally to change the tools, their order and their default parameters, or even used as subworkflows. Moreover, users can also design novel meaningful workflows via the Galaxy workflow interface using the >100 available tools.

Analysis of raw metagenomic or metatranscriptomic shotgun data

The workflow quickly produces, from raw metagenomic or metatranscriptomic shotgun data, accurate and precise taxonomic assignations, wide extended functional results and taxonomically related metabolism information (Figure 1). This workflow consists of i) processing with quality control/trimming (FastQC and Trim Galore!) and dereplication (VSearch [10]; ii) taxonomic analyses with assignation (MetaPhlAn2 [16]) and visualization (KRONA , GraPhlAn); iii) functional analyses with metabolic assignation and pathway reconstruction (HUMAnN2 [17]); iv) functional and taxonomic combination with developed tools combining HUMAnN2 and MetaPhlAn2 outputs.

This workflow has been tested on two mock metagenomic datasets with controlled communities (Supplementary material). We have compared the extracted taxonomic and functional information to such information extracted with the EBI metagenomics' pipeline and to the expectations from the mock datasets, to illustrate the potential of the ASaiM workflow. With ASaiM, we generate accurate and precise data for taxonomic analyses (Figure 2) and we can access information at the level of the species. More functional information (*e.g.* gene families, gene ontologies, pathways) are also extracted with ASaiM compared to the ones available on EBI metagenomics. With this workflow, we can go one step further and

investigate which taxons are involved in a specific pathway or a gene family (*e.g.* involved species and their relative involvement in different step of fatty acid biosynthesis pathways, Figure 3).

For the tests, ASaiM was deployed on a computer with Debian GNU/Linux System, 8 cores Intel(R) Xeon(R) at 2.40 GHz and 32 Go of RAM. The workflow processed the 1,225,169 and 1,386,198 454 GS FLX Titanium reads of each datasets, with a stable memory usage, in 4h44 and 5h22 respectively (Supplementary material). The execution time is logarithmically linked to the input data size. With this workflow, it is then easy and quick to process raw microbiota data and extract diverse useful information.

Assembly of metagenomics data

Microbiota data usually come with quite short reads. To reconstruct genomes or to get longer sequences for further analysis, microbiota sequences have to be assembled with dedicated metagenome assemblers. To help in this task, two workflows have been developed in ASaiM, each one using one of the well-performing assemblers [42–48]: MEGAHIT [36] and MetaSPAdes [37]. Both workflows consists of: 1) processing with quality control/trimming (FastQC and Trim Galore!); ii) assembly with either MEGAHIT or MetaSPAdes; iii) estimation of the assembly quality statistics with MetaQUAST [38]; iv) identification of potential assembly error signature with VALET; v) determination of percentage of unmapped reads with Bowtie2 (Bowtie , RRID:SCR_005476)[31] combined with MultiQC [25] to aggregate the results.

Analysis of metataxonomic data

To analyze amplicon or ITS data, the Mothur and QIIME tool suites are available to ASaiM. We integrated the workflows described in tutorials of Mothur and QIIME, as example of metataxonomic data analyses as well as support for the training material.

Running as in EBI metagenomics

As the tools used in the EBI Metagenomics pipeline (version 3) are also available in ASaiM, we integrate them in a workflow with the same steps as the EBI Metagenomics pipeline. Analyses made in EBI Metagenomics website can be then reproduced locally, without having to wait for availability of EBI Metagenomics or to upload any data on EBI Metagenomics. However the parameters must be defined by the user as we can not find them on EBI Metagenomics documentation. In ASaiM, the entire provenance and every parameters are tracked to guarantee the reproducibility.

Documentation and training

A tool or software is easier to use if it is well documented. Hence extensive documentation helps the users to be familiar with the tool and also prevents mis-usage. For ASaiM, we developed an extensive online documentation (http://asaim.readthedocs.io), mainly to explain how to use it, how to deploy it, which tools are integrated with small documentation about these tools, which workflows are available and how to use them.

In addition to this online documentation, training materials have been developed. Some Galaxy interactive tours are included inside the Galaxy instance, to guide users through entire microbiota analyses in an interactive (step-by-step) way. We also developed several step-by-step tutorials to explain the concepts of microbiota analyses, the different tools and parameters and ASaiM workflows with toy datasets. Hosted in the Galaxy Training Network (GTN) GitHub repository (https://github.com/galaxyproject/training-material), the tutorials are available online at http://training.galaxyproject.org/topics/metagenomics and also directly accessible from ASaiM and its documentation for self-training. These tutorials and ASaiM have been used during several workshops on metagenomics data analysis, and some undergrads courses to explain and use the EBI Metagenomics workflow in a reproducible way. ASaiM is also used as support for a citizen science and education project (Beer DeCoded [49]).

Installation and running ASaiM

Running the containerized ASaiM simply requires to install Docker and to start the ASaiM image with:

```
$ docker run -d -p 8080:80 quay.io/bebatut/asaim-framework:latest
```

As Galaxy, ASaiM is production-ready and can be configured to use external accessible computer clusters or cloud environments. It is also possible and easy to install all or only a subset of tools of the ASaiM framework on existing Galaxy instances, as we did on the European Galaxy instance (https://metagenomics.usegalaxy.eu). More details about the installation and the use of ASaiM are available on the online documentation (http://asaim.readthedocs.io/en/latest/installation.html).

Conclusion

ASaiM provides a powerful framework to easily and quickly analyze microbiota data in a reproducible, accessible and transparent way. Built on a Galaxy instance wrapped in a Docker image, ASaiM can be easily deployed with its extensive set of tools and their dependencies, saving users from the hassle of installing all software. These tools are complemented with a set of predefined and tested workflows to address the main questions of microbiota research (assembly, community structure and function). All these tools and workflows are extensively documented online (http://asaim.readthedocs.io) and supported by Interactive Tours and tutorials.

With this complete infrastructure, ASaiM offers a sophisticated environment for microbiota analyses to any scientists, while promoting transparency, sharing and reproducibility.

Methods

For the tests, ASaiM was deployed on a computer with Debian GNU/Linux System, 8 cores Intel(R) Xeon(R) at 2.40 GHz and 32 Go of RAM. The workflow has been run on two mock community samples of Human Microbiome Project (HMP), containing a genomic mixture of

22 known microbial strains. The details of comparison analyses are described in the Supplementary Material.

Availability of supporting data

Archival copies of the code and mock data are available in the GigaScience GigaDB repository [50].

Availability of supporting source code and requirements

- Project name: ASaiM
- Project home page: https://github.com/ASaiM/framework
- Operating system(s): Platform independent
- Other requirements: Docker
- License: Apache 2
- RRID: SCR_015878GTN

All tools described herein are available in the Galaxy Toolshed (https://toolshed.g2.bx.psu.edu). The Dockerfile to automatically install deploy ASaiM is provided in the GitHub repository and a pre-built Docker image is available at https://quay.io/repository/bebatut/asaim-framework.

Declarations

Abbreviations

API: application programming interface; AsaiM: Auvergne Sequence analysis of intestinal Microbiota; CPU: central processing unit; Galaxy Training Network; HMP: Human Microbiome Project; ITS: Internal transcribed spacer

Competing interests

The author(s) declare that they have no competing interests.

Funding

The Auvergne Regional Council and the European Regional Development Fund have supported this work.

Authors' contributions

BB, KG, CD, SH, JFB, EP, PP contributed equally to the conceptualization, to the methodology and to the writing process. JFB, PP contributed equally to the funding acquisition. BB, KG, SH contributed equally to the software development and BB, KG, CD and JFB to the validation.

Acknowledgements

The authors would like to thank EA 4678 CIDAM, UR 454 INRA, M2iSH, LIMOS, AuBi, Mésocentre, de.NBI for their involvement in this project, as well as Réjane Beugnot, Thomas Eymard, David Parsons and Björn Grüning for their help.

References

1. Ladoukakis E, Kolisis FN, Chatziioannou AA. Integrative workflows for metagenomic analysis. Front Cell Dev Biol. 2014;2:70.

2. Kuczynski J, Stombaugh J, Walters WA, González A, Gregory Caporaso J, Knight R. Using QIIME to Analyze 16S rRNA Gene Sequences from Microbial Communities. Current Protocols in Microbiology. 2012. p. 1E.5.1–1E.5.20.

3. Schloss PD, Westcott SL, Ryabin T, Hall JR, Hartmann M, Hollister EB, et al. Introducing mothur: open-source, platform-independent, community-supported software for describing and comparing microbial communities. Appl Environ Microbiol. 2009;75:7537–41.

4. Nekrutenko A, Taylor J. Next-generation sequencing data interpretation: enhancing reproducibility and accessibility. Nat Rev Genet. 2012;13:667–72.

5. Meyer F, Paarmann D, D'Souza M, Olson R, Glass EM, Kubal M, et al. The metagenomics RAST server – a public resource for the automatic phylogenetic and functional analysis of metagenomes. BMC Bioinformatics. 2008;9:386.

6. Hunter S, Corbett M, Denise H, Fraser M, Gonzalez-Beltran A, Hunter C, et al. EBI metagenomics--a new resource for the analysis and archiving of metagenomic data. Nucleic Acids Res. 2014;42:D600–6.

7. Goecks J, Nekrutenko A, Taylor J, Galaxy Team. Galaxy: a comprehensive approach for supporting accessible, reproducible, and transparent computational research in the life sciences. Genome Biol. 2010;11:R86.

8. Afgan E, Baker D, van den Beek M, Blankenberg D, Bouvier D, Čech M, et al. The Galaxy platform for accessible, reproducible and collaborative biomedical analyses: 2016 update. Nucleic Acids Res. 2016;44:W3–10.

9. Wood DE, Salzberg SL. Kraken: ultrafast metagenomic sequence classification using exact alignments. Genome Biol. 2014;15:R46.

10. Rognes T, Flouri T, Nichols B, Quince C, Mahé F. VSEARCH: a versatile open source tool for metagenomics. PeerJ. 2016;4:e2584.

11. Kosakovsky Pond S, Wadhawan S, Chiaromonte F, Ananda G, Chung W-Y, Taylor J, et al. Windshield splatter analysis with the Galaxy metagenomic pipeline. Genome Res. 2009;19:2144–53.

12. Grüning BA, Fallmann J, Yusuf D, Will S, Erxleben A, Eggenhofer F, et al. The RNA workbench: best practices for RNA and high-throughput sequencing bioinformatics in Galaxy. Nucleic Acids Res. 2017; doi:10.1093/nar/gkx409

13. Blankenberg D, Von Kuster G, Bouvier E, Baker D, Afgan E, Stoler N, et al. Dissemination of scientific software with Galaxy ToolShed. Genome Biol. 2014;15:403.

14. Sloggett C, Goonasekera N, Afgan E. BioBlend: automating pipeline analyses within Galaxy and CloudMan. Bioinformatics. 2013;29:1685–6.

15. Grüning B, Dale R, Sjödin A, Rowe J, Chapman BA, Tomkins-Tinch CH, et al. Bioconda: A sustainable and comprehensive software distribution for the life sciences. bioRxiv. 2017. http://dx.doi.org/10.1101/207092

16. Truong DT, Franzosa EA, Tickle TL, Scholz M, Weingart G, Pasolli E, et al. MetaPhlAn2 for enhanced metagenomic taxonomic profiling. Nat Methods. 2015;12:902–3.

17. Abubucker S, Segata N, Goll J, Schubert AM, Izard J, Cantarel BL, et al. Metabolic reconstruction for metagenomic data and its application to the human microbiome. PLoS Comput Biol. 2012;8:e1002358.

18. Batut B, Grüning B. ENASearch: A Python library for interacting with ENA's API. The Journal of Open Source Software. 2017;2:418.

19. Li H. A statistical framework for SNP calling, mutation discovery, association mapping and population genetical parameter estimation from sequencing data. Bioinformatics. 2011;27:2987–93.

20. Li H. Improving SNP discovery by base alignment quality. Bioinformatics. 2011;27:1157–8.

21. Li H, Handsaker B, Wysoker A, Fennell T, Ruan J, Homer N, et al. The Sequence Alignment/Map format and SAMtools. Bioinformatics. 2009;25:2078–9.

22. McDonald D, Clemente JC, Kuczynski J, Rideout JR, Stombaugh J, Wendel D, et al. The Biological Observation Matrix (BIOM) format or: how I learned to stop worrying and love the ome-ome. Gigascience. 2012;1:7.

23. Schmieder R, Edwards R. Quality control and preprocessing of metagenomic datasets. Bioinformatics. 2011;27:863–4.

24. Bolger AM, Lohse M, Usadel B. Trimmomatic: a flexible trimmer for Illumina sequence data. Bioinformatics. 2014;30:2114–20.

25. Ewels P, Magnusson M, Lundin S, Käller M. MultiQC: summarize analysis results for multiple tools and samples in a single report. Bioinformatics. 2016;32:3047–8.

26. Fu L, Niu B, Zhu Z, Wu S, Li W. CD-HIT: accelerated for clustering the next-generation sequencing data. Bioinformatics. 2012;28:3150–2.

27. Kopylova E, Noé L, Touzet H. SortMeRNA: fast and accurate filtering of ribosomal RNAs in metatranscriptomic data. Bioinformatics. 2012;28:3211–7.

28. Rho M, Tang H, Ye Y. FragGeneScan: predicting genes in short and error-prone reads. Nucleic Acids Res. 2010;38:e191–e191.

29. Li H, Durbin R. Fast and accurate long-read alignment with Burrows-Wheeler transform. Bioinformatics. 2010;26:589–95.

30. Press AR. Ultrafast and Memory-Efficient Alignment of Short DNA Sequences to the Human Genome. CreateSpace; 2015.

31. Camacho C, Coulouris G, Avagyan V, Ma N, Papadopoulos J, Bealer K, et al. BLAST+: architecture and applications. BMC Bioinformatics. 2009;10:421.

32. Cock PJA, Chilton JM, Grüning B, Johnson JE, Soranzo N. NCBI BLAST+ integrated into Galaxy. Gigascience. 2015;4:39.

33. Buchfink B, Xie C, Huson DH. Fast and sensitive protein alignment using DIAMOND. Nat Methods. 2015;12:59–60.

34. Mistry J, Finn RD, Eddy SR, Bateman A, Punta M. Challenges in homology search: HMMER3 and convergent evolution of coiled-coil regions. Nucleic Acids Res. 2013 Jul;41(12):e121. doi: 10.1093/nar/gkt263.

35. Rodriguez-R LM, Konstantinidis KT. Nonpareil: a redundancy-based approach to assess the level of coverage in metagenomic datasets. Bioinformatics. 2014;30:629–35.

36. Li D, Luo R, Liu C-M, Leung C-M, Ting H-F, Sadakane K, et al. MEGAHIT v1.0: A fast and scalable metagenome assembler driven by advanced methodologies and community practices. Methods. 2016;102:3–11.

37. Nurk S, Meleshko D, Korobeynikov A, Pevzner PA. metaSPAdes: a new versatile metagenomic assembler. Genome Res. 2017;27:824–34.

38. Mikheenko A, Saveliev V, Gurevich A. MetaQUAST: evaluation of metagenome assemblies. Bioinformatics. 2016;32:1088–90.

39. Langille MGI, Zaneveld J, Caporaso JG, McDonald D, Knights D, Reyes JA, et al. Predictive functional profiling of microbial communities using 16S rRNA marker gene sequences. Nat Biotechnol. 2013;31:814–21.

40. Asnicar F, Weingart G, Tickle TL, Huttenhower C, Segata N. Compact graphical representation of phylogenetic data and metadata with GraPhlAn. PeerJ. 2015;3:e1029.

41. Ondov BD, Bergman NH, Phillippy AM. Interactive metagenomic visualization in a Web browser. BMC Bioinformatics. 2011;12:385.

42. Awad S, Irber L, Titus Brown C. Evaluating Metagenome Assembly on a Simple Defined Community with Many Strain Variants [Internet]. 2017. Available from: http://dx.doi.org/10.1101/155358

43. Greenwald WW, Klitgord N, Seguritan V, Yooseph S, Venter JC, Garner C, et al. Utilization of defined microbial communities enables effective evaluation of meta-genomic assemblies. BMC Genomics. 2017;18:296.

44. Olson ND, Treangen TJ, Hill CM, Cepeda-Espinoza V, Ghurye J, Koren S, et al. Metagenomic assembly through the lens of validation: recent advances in assessing and improving the quality of genomes assembled from metagenomes. Brief Bioinform. 2017; http://dx.doi.org/10.1093/bib/bbx098

45. Quince C, Walker AW, Simpson JT, Loman NJ, Segata N. Shotgun metagenomics, from sampling to analysis. Nat Biotechnol. 2017;35:833–44.

46. Sczyrba A, Hofmann P, Belmann P, Koslicki D, Janssen S, Dröge J, et al. Critical Assessment of Metagenome Interpretation-a benchmark of metagenomics software. Nat Methods. 2017;14:1063–71.

47. van der Walt AJ, Van Goethem MW, Ramond J-B, Makhalanyane TP, Reva O, Cowan DA. Assembling Metagenomes, One Community At A Time [Internet]. 2017. Available from: http://dx.doi.org/10.1101/120154

48. Vollmers J, Wiegand S, Kaster A-K. Comparing and Evaluating Metagenome Assembly Tools from a Microbiologist’s Perspective - Not Only Size Matters! PLoS One. 2017;12:e0169662.

49. Sobel J, Henry L, Rotman N, Rando G. BeerDeCoded: the open beer metagenome project. F1000Res. 2017;6:1676.

50. Batut, B; Gravouil, K; Defois, C; Hiltemann, S; Brugère, J; Peyretaillade, E; Peyre, P (): Supporting data for "ASaiM: a Galaxy-based framework to analyze microbiota data" GigaScience Database. http://dx.doi.org/10.5524/100451

Figure 1: Main ASaiM workflow to analyze raw sequences.

This workflow takes as input a dataset of raw shotgun sequences (in FastQ format) from microbiota, preprocess it (yellow boxes), extracts taxonomic (red boxes) and functional (purple boxes) assignations and combines them (green boxes).

Image available under CC-BY license (https://doi.org/10.6084/m9.figshare.5371396.v3)

Figure 2: Comparisons of the community structure for SRR072233.

This figure compares the community structure between the expectations (mapping of the sequences on the expected genomes), data found on EBI Metagenomics database (extracted with the EBI Metagenomics pipeline) and the results of the main ASaiM workflow (Figure 1).

Figure 3: Example of an investigation of the relation between community structure and functions. The involved species and their relative involvement in fatty acid biosynthesis pathways have been extracted with ASaiM workflow (Figure 1) for SRR072233

Figure 1: Main ASaiM workflow to analyze raw sequences.

Click here to download Figure main_workflow.pdf

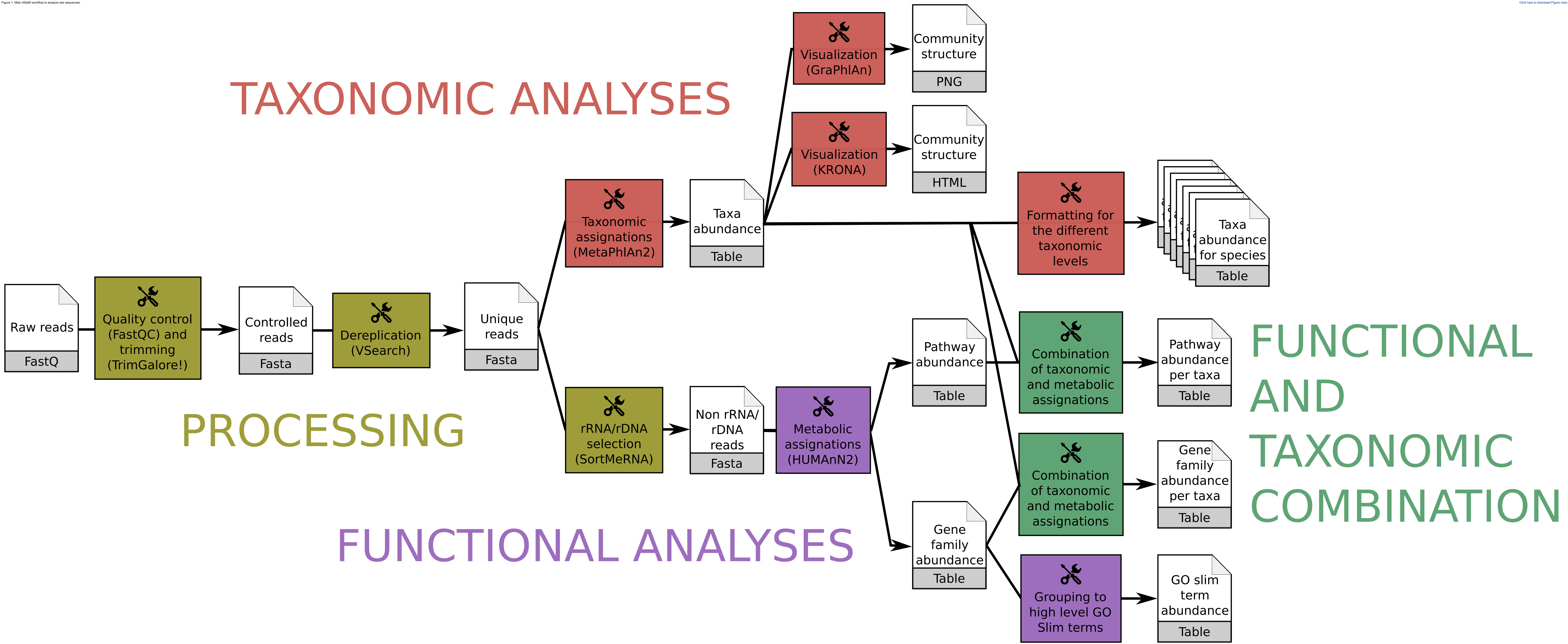

Figure 2: Comparisons of the community structure for SRR072233

Click here to download Figure hmp_taxonomic_results.pdf

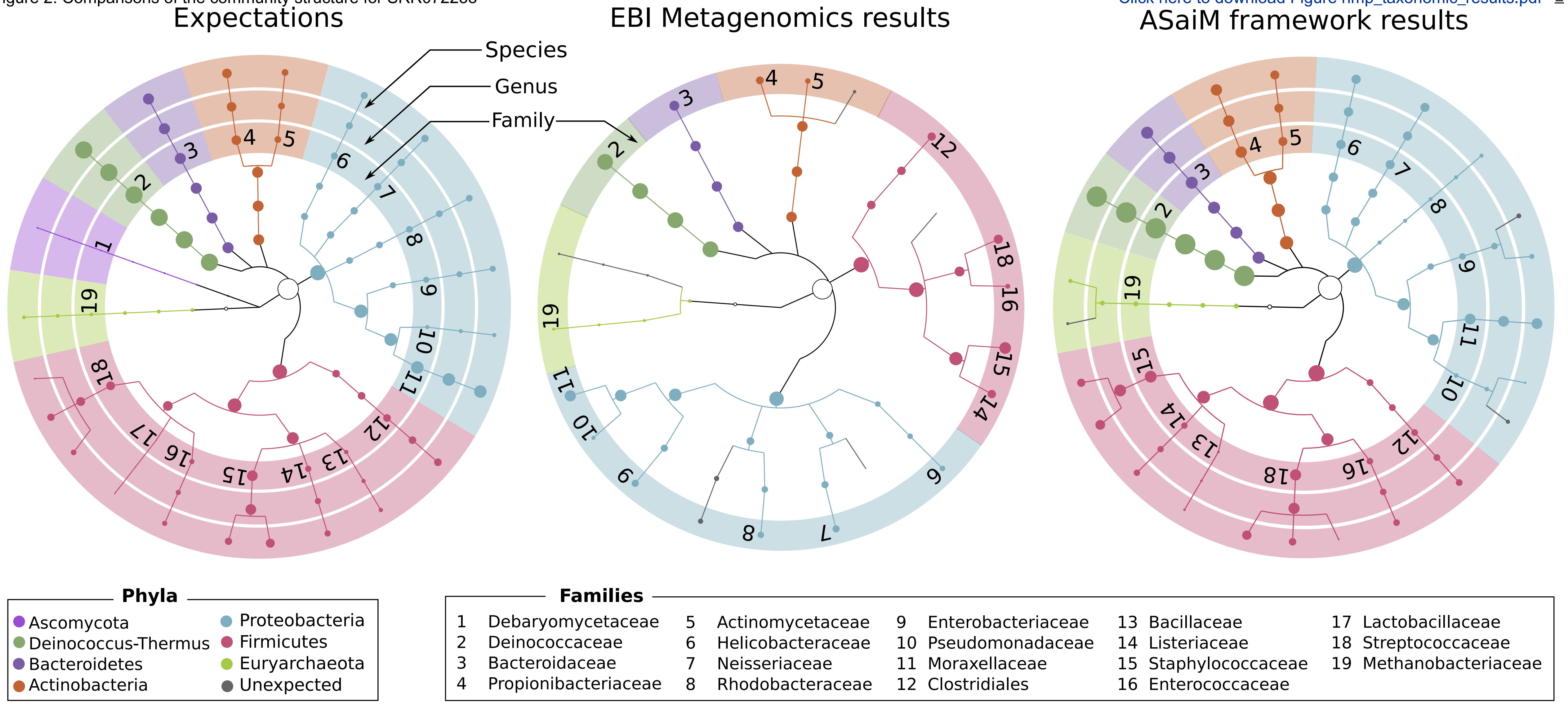

Figure 3: Example of an investigation of the relation between community structure and functions

Click here to download Figure hmp_taxonomically_related_functional_results.pdf

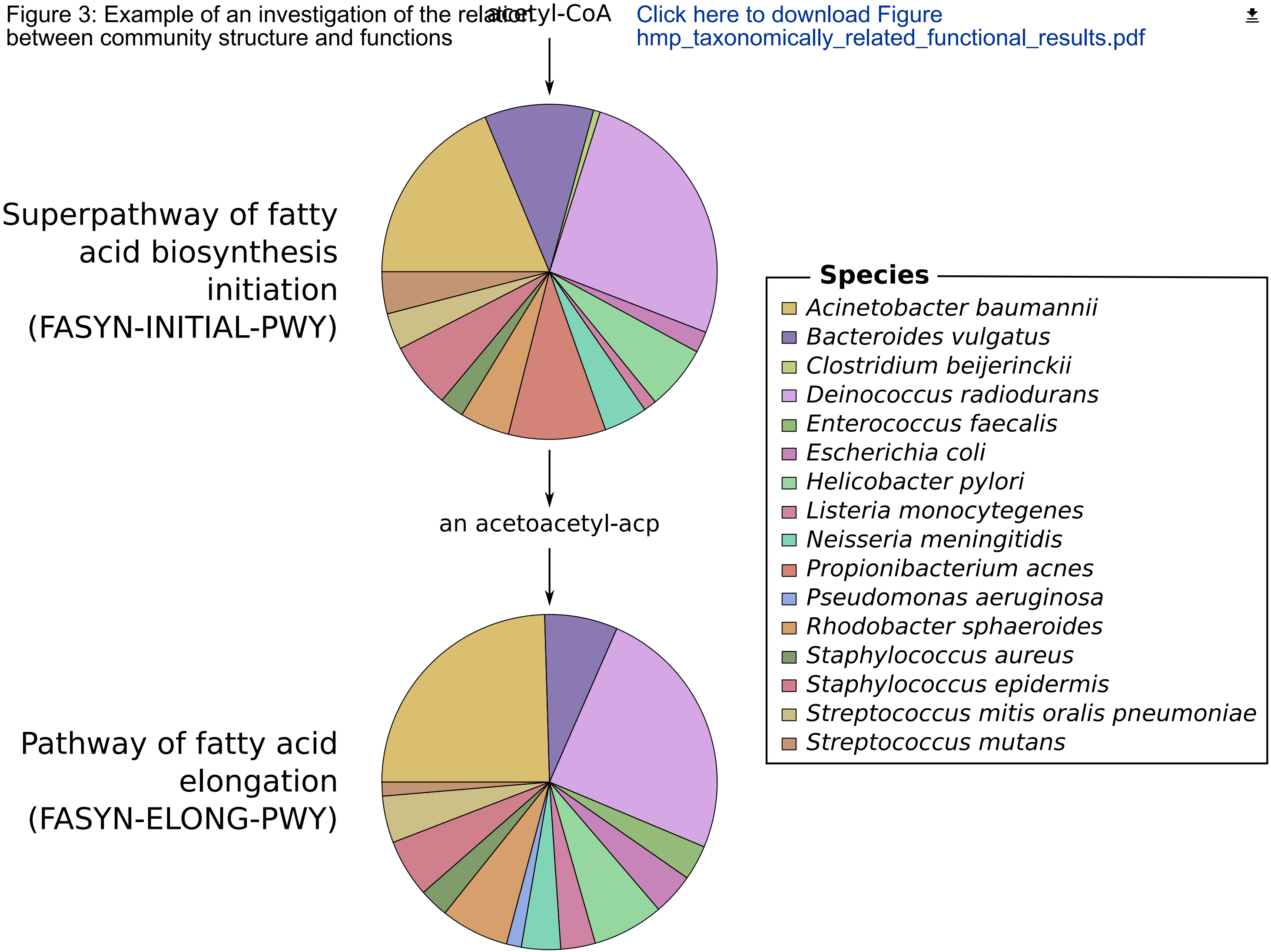

Click here to access/download
Supplementary Material
sup_mat_1.pdf
