# Supplementary material for: ASaiM: a Galaxy-based framework to analyze microbiota data
Source: Gigascience. 2018 May 15;7(6):giy057. doi: 10.1093/gigascience/giy057 (PMC6007547; doi:10.1093/gigascience/giy057)
Supplement: Supplemental material [file giy057_supplemental_file.pdf]

Validation of the ASaiM framework and its workflows on HMP mock community samples

The ASaiM framework and its workflows have been tested and validated on two mock metagenomic data of an artificial community (with 22 known microbial strains). The datasets are available on *EBI metagenomics* database (project accession number: SRP004311). First we checked that the targeted abundances (based on number of PCR product) from both mock datasets were similar to the effective abundance (by mapping reads on reference genomes). Second, taxonomic and functional results produced by the ASaiM framework have been extensively analyzed and compared to expectations and to results obtained with the *EBI metagenomics* pipeline (S. Hunter et al. 2014).

For these datasets, the ASaiM framework produces accurate and precise taxonomic assignations, different functional results (gene families, pathways, GO slim terms) and results combining taxonomic and functional information. Despite almost 1.4 million of raw metagenomic sequences, these analyses were executed in less than 6h on a commodity computer. Hence, the ASaiM framework and its workflows are proven to be relevant for the analysis of microbiota datasets.

1 Data

On *EBI metagenomics* database, two mock community samples for Human Microbiome Project (HMP) are available. Both samples contain a genomic mixture of 22 known microbial strains. Relative abundance of each strain has been targeted using the number of PCR product of their respective 16S sequences (Table 1). In first sample (SRR072232), the targeted 16S copies of the strains vary by up to four orders of magnitude between the strains (Table 1), whereas in second sample (SRR072233) the same 16S copy number is targeted for each strain (Table 1). After pooling, the DNA of the strains of both samples were sequenced using 454 GS FLX Titanium. 1,225,169 and 1,386,198 raw metagenomic sequences are then respectively obtained for the first dataset (SRR072232) and the second dataset (SRR072233).

Kingdom	Phylum	Class	Order	Taxonomy Family	Genus	Species	Strains	Targeted abundances (%) SRR072232	SRR072233
Archaea	Euryarchaeota	Methanobacteria	Methanobacteriales	Methanobacteriaceae	*Methanobrevibacter*	*Methanobrevibacter smithii*	ATCC 35061	$1.797 \cdot 10^{1}$	4.545
Bacteria	Actinobacteria	Actinobacteria	Actinomycetales	Actinomycetaceae	*Actinomyces*	*Actinomyces odontolyticus*	ATCC 17982	$1.797 \cdot 10^{-2}$	4.545
				Propionibacteriaceae	*Propionibacterium*	*Propionibacterium acnes*	DSM 16379	$1.797 \cdot 10^{-1}$	4.545
	Bacteroidetes	Bacteroidia	Bacteroidales	Bacteroidaceae	*Bacteroides*	*Bacteroides vulgatus*	ATCC 8482	$1.797 \cdot 10^{-2}$	4.545
	Deinococcus-Thermus	Deinococci	Deinococcales	Deinococcaceae	*Deinococcus*	*Deinococcus radiodurans*	DSM 20539	$1.797 \cdot 10^{-2}$	4.545
	Firmicutes	Bacilli	Bacillales	Bacillaceae	*Bacillus*	*Bacillus cereus thuringiensis*	ATCC 10987	1.797	4.545
				Listeriaceae	*Listeria*	*Listeria monocytogenes*	ATCC BAA-679	$1.797 \cdot 10^{-1}$	4.545
				Staphylococcaceae	*Staphylococcus*	*Staphylococcus aureus*	ATCC BAA-1718	1.797	4.545
						Staphylococcus epidermidis	ATCC 12228	$1.797 \cdot 10^{1}$	4.545
			Lactobacillales	Enterococcaceae	*Enterococcus*	*Enterococcus faecalis*	ATCC 47077	$1.797 \cdot 10^{-2}$	4.545
				Lactobacillaceae	*Lactobacillus*	*Lactobacillus gasseri*	DSM 20243	$1.797 \cdot 10^{-2}$	4.545
				Streptococcaceae	*Streptococcus*	*Streptococcus agalactiae*	ATCC BAA-611	1.797	4.545
						Streptococcus mutans	ATCC 700610	$1.797 \cdot 10^{1}$	4.545
						Streptococcus mitis oralis pneumoniae	ATCC BAA-334	$1.797 \cdot 10^{-2}$	4.545
		Clostridia	Clostridiales	Clostridiaceae	*Clostridium*	*Clostridium beijerinckii*	ATCC 51743	1.797	4.545
	Proteobacteria	Alphaproteobacteria	Rhodobacterales	Rhodobacteraceae	*Rhodobacter*	*Rhodobacter sphaeroides*	ATCC 17023	$1.797 \cdot 10^{1}$	4.545
		Betaproteobacteria	Neisseriales	Neisseriaceae	*Neisseria*	*Neisseria meningitidis*	ATCC BAA-335	$1.797 \cdot 10^{-1}$	4.545
		Epsilonproteobacteria	Campylobacterales	Helicobacteraceae	*Helicobacter*	*Helicobacter pylori*	ATCC 700392	$1.797 \cdot 10^{-1}$	4.545
		Gammaproteobacteria	Pseudomonadales	Moraxellaceae	*Acinetobacter*	*Acinetobacter baumannii*	ATCC 17978	$1.797 \cdot 10^{-1}$	4.545
				Pseudomonadaceae	*Pseudomonas*	*Pseudomonas aeruginosa*	ATCC 47085	1.797	4.545
			Enterobacteriales	Enterobacteriaceae	*Escherichia*	*Escherichia coli*	ATCC 70096	$1.797 \cdot 10^{1}$	4.545
Fungi	Ascomycota	Saccharomycetes	Saccharomycetales	Debaryomycetaceae	*Candida*	*Candida albicans*	SC5314	$1.797 \cdot 10^{-2}$	4.545

Table 1: Expected strains, their taxonomy and their targeted relative abundance (percentage) based on 16S gene copy counts (abundance, from metadata on *EBI metagenomics* database) on both samples (SRR072232 and SRR072233)

2 Methods

Both datasets have been analyzed using the ASaiM framework. The results are extensively analyzed and compared to expected results from reference genome information and to *EBI metagenomics* results. Details about these analyses (workflows, scripts) are available on a dedicated GitHub repository (https://github.com/ASaiM/hmp_mock_tests).

2.1 Abundance computation using mapping on reference genomes

In both datasets, abundance of each strain is targeted based on 16S quantity to build the genomic mixture before sequencing. But these targeted abundances may not reflect the final abundances (*e.g.* 16S copy number variation, sequencing bias). We have a known composition of the datasets with an expected abundances before sequencing but no information about the real abundance of the strains after sequencing.

Before any analysis of *EBI metagenomics* and ASaiM results, we need more insights in the real abundance of the strains after sequencing. We mapped raw reads on reference genomes of expected strains using BWA 0.7.12 (Li and Durbin 2009; Li and Durbin 2010) (using default parameters). We then extract the "exact" abundances of expected strains in the metagenomic datasets, after DNA pooling and sequencing (*i.e.* not based on targeted rRNA operon counts in PCR).

Similar community compositions are observed using mapping-based relative abundances of strains or targeted relative abundance (Figure 1): the Bray-Curtis dissimilarity scores are smaller than 0.5 (0.338 for SRR02232 and 0.479 for SRR072233). However, for SRR072233 (Figure 1), identical targeted abundances are expected for all species, but variations are observed for mapping based abundances. The variation of 16S gene copy number between the species can explain the differences between targeted abundances and mapping-based abundances. Indeed, the targeted abundances are based on 16S copy number targeted in PCR to build the DNA pool. But, the number of 16S gene copies is not identical in the strains (from 1 for *Candida albicans* to 14 for *Clostridium beijerinckii*). Hence, even with identical targeted abundances (*e.g.* for SRR072233), we expect that a species with two 16S gene copies in its genome would be found twice less abundant in mapping-based relative abundance results. The 16S gene copy number variation induces then a difference between the relative abundance based on mapping reads on whole genome and the expected relative abundance based on the targeted 16S gene counts.

Taxonomic analyses in *EBI metagenomics* and ASaiM workflows are executed on metagenomic sequences, *i.e.* on data after DNA pooling and sequencing. Mapping-based relatives abundances computed on raw metagenomic sequences are then more appropriate expected abundance information than the relative abundances based on 16S counts. We will then use this information in the next sections.

2.2 Analyses using *EBI Metagenomics*

In *EBI metagenomics* database, both datasets have been analysed with *EBI metagenomics* pipeline (Version 1.0) (Figure 2).

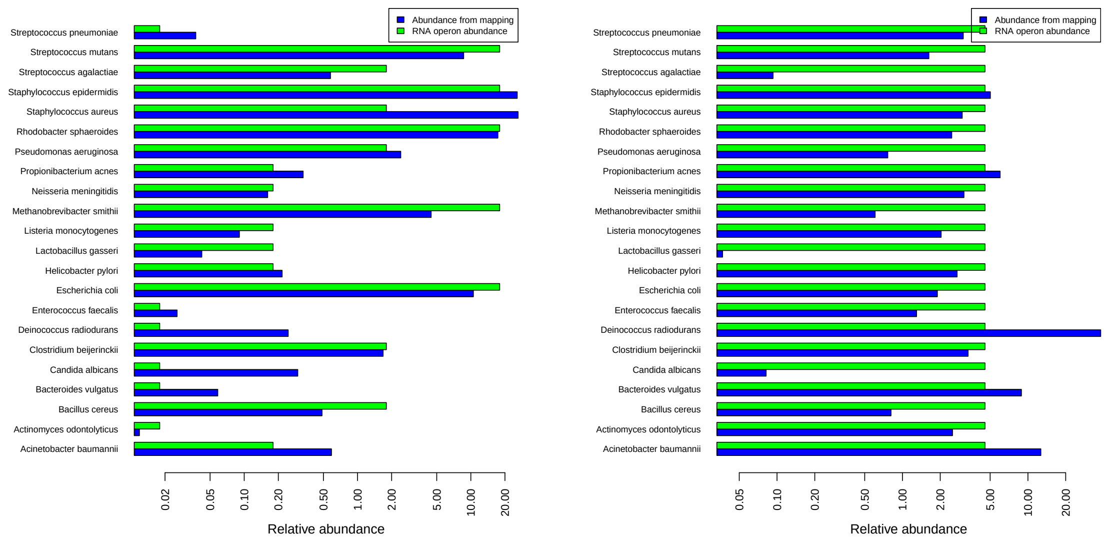

Figure 1: Comparison of relative abundances (percentage, in log scale) between expectation given the ribosomal RNA operon counts (green, Table 1) and mapping against reference genomes for both samples (SRR072232 on left, SRR072233 on right)

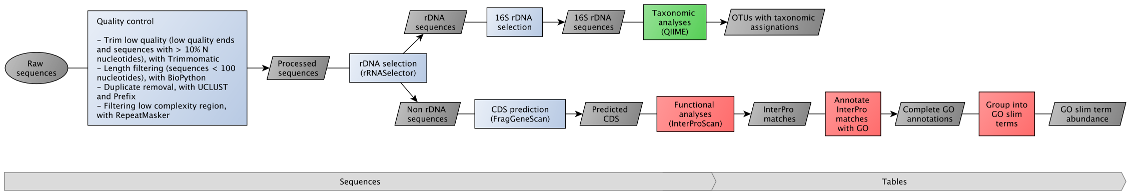

Figure 2: *EBI metagenomics* pipeline (version 1.0). The grey boxes correspond to data, the blue boxes to pretreatment steps, the red boxes to functional analysis steps and the green boxes to taxonomic analysis steps.

To ease comparison with ASaiM results, *EBI metagenomics* pipeline results were downloaded from *EBI metagenomics* database and formatted. First, to compute relative abundances of each clade at all taxonomic levels, OTUs with taxonomic assignation are extracted and aggregated. Second, *EBI metagenomics* pipeline generates 3 types of functional results (Figure 2): matches with InterPro, complete GO annotations and GO slim annotations. Here, we focus on GO slim annotations. The annotations are formatted to extract relative abundances (in percentage) of GO slim term annotations inside each GO slim term category (cellular components, biological processes and molecular functions).

2.3 Analyses using ASaiM framework

Main workflow (Supplementary material 1) of the ASaiM framework is used to analyze both datasets. The ASaiM framework were deployed on a computer with Debian GNU/Linux System, 8 cores Intel(R) Xeon(R) at 2.40 GHz and 32 Go of RAM. On this computer, the workflow execution is relatively fast: $<$ 5h and $<$ 5h30 for datasets with 1,225,169 and 1,386,198 sequences respectively (Table 2). The most time-consuming step is functional profiling using *HUMAnN2* (Abubucker et al. 2012) which last $\simeq$ 64% of overall time execution (Table 2). Size of the process in memory is stable over workflow execution (variability inferior to 40 kb) (Table 2).

Statistics		SRR072232	SRR072233
Execution time	**Whole workflow**	**4h44**	**5h22**
	PRINSEQ	0h38	0h44
	Vsearch	16s	19s
	SortMeRNA	0h55	0h58
	MetaPhlAN2	0h09	0h10
	HUMAnN2	3h01	3h26
Size of the process in memory (kb)	Min	1,515,732	1,515,732
	Mean	1,515,744	1,515,743
	Max	1,515,768	1,515,764

Table 2: Computation statistics on ASaiM for both samples (SRR072233 and SRR072233)

To compare taxonomic and functional results of both datasets, we used the comparative analysis workflows available with the ASaiM framework (Supplementary material 1).

To check the taxonomic results, we checked that each expected organism can be found using same tools and databases than in ASaiM. A dataset is then built for each reference genome. To build these datasets, the reference genome of each expected organism is randomly cut in smaller sequences such as the size distribution of sequences is identical to the one in SRR072232 database after quality control and dereplication, with same sequence number. Taxonomic assignation for each dataset is then extracted using MetaPhlAN (Truong et al. 2015; Segata et al. 2012).

2.4 Comparison of *EBI metagenomics* results and ASaiM results

The first step in the comparison of *EBI metagenomics* results and ASaiM results is the comparison of rDNA sequences extracted with both methods to determine if similar rDNA sequences are found with both methods. We first compare the extracted rDNA sequences using the names of the corresponding raw sequences. However, rDNA sequence extraction process is executed after quality treatment and dereplication in both pipelines. Some duplicated sequences were then eliminated during dereplication process and the pool of sequences are then not comparable using only their names. To compare rDNA sequences, we run blastn 2.2.31 (Camacho et al. 2009) on rDNA sequences found with *EBI metagenomics* against rDNA sequences found with ASaiM. Sequences are considered as similar between both pipelines if the similarity percentage is higher than 98% on more than 98% of the sequence length and if the e-value is below $1{\cdot}10^{-16}$. We also compare to expected rDNA sequences: we run SortMeRNA with same parameters as in ASaiM but with a database made of rDNAs extracted from the reference genomes of the expected organisms.

In ASaiM framework, *MetaPhlAn* computes the relative abundance of clades only on assigned reads. No count is made of non assigned reads unlike *EBI metagenomics* pipeline. To compare relative abundances between both pipelines, we focus on relative abundances computed on OTUs or reads with a complete taxonomic assignation from kingdom to family. These results are also compared to relative mapping-based abundances.

Both *EBI metagenomics* and ASaiM workflows group functional matches into GO slim terms, a subset of the terms in the whole Gene Ontology focusing on microbial metabolic functions. These GO slim terms give a broad overview of the ontology content. To compare *EBI metagenomics* and ASaiM results, relative abundance of GO slim terms for both samples and both workflows are concatenated and compared, given the workflow depicted in Figure 3.

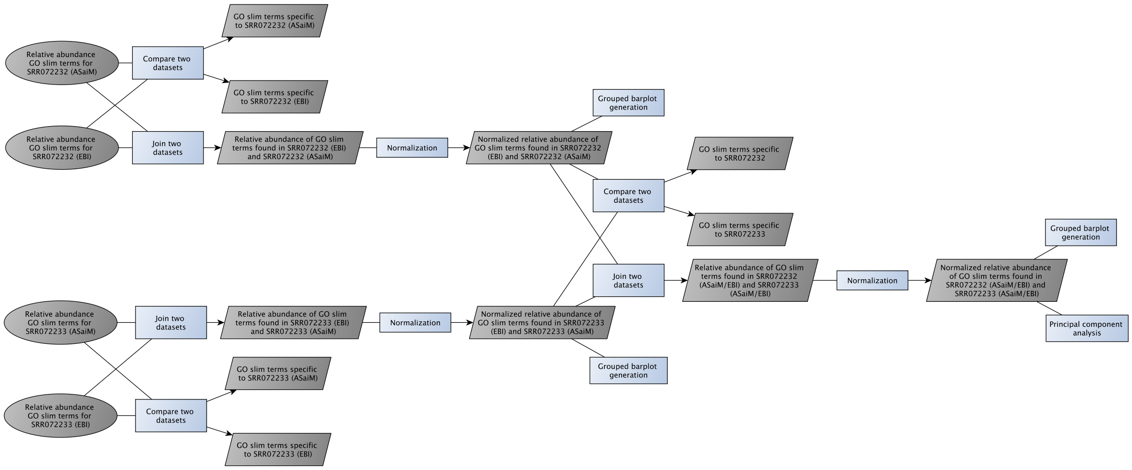

Figure 3: Workflow to compare GO slim annotation abundances between samples (SRR072232, SRR072233) and workflows (*EBI metagenomics*, ASaiM). This workflow is available with ASaiM Galaxy instance. The grey boxes correspond to data, the blue boxes to processing steps.

3 Results

3.1 Preprocessing steps

In both workflows, raw sequences are pre-processed before any taxonomic or functional analysis. These preprocessing steps include a quality control to remove low quality, small or duplicated sequences and also a step to sort rNA/rDNA sequences from non rRNA/rDNA sequences.

The tools and the parameters in the ASaiM framework differ from the ones used in *EBI metagenomics* pipeline. We then observe different preprocessing outputs (Table 3).

The number of sequence after quality control and dereplication are different between both pipelines (Table 3). ASaiM framework conserves more sequences (> 96 %) during these first steps of quality

	SRR072232				SRR072233			
Sequences	EBI		ASaiM		EBI		ASaiM	
Raw sequences	1,225,169				1,386,198			
Sequences after quality control and dereplication	997,622	81.4%	1,175,853	96%	1,197,748	86.4%	1,343,451	96.9%
rDNA sequences	9,453	0.95%	16,016	1.4%	9,698	0.81%	13,850	1%
non rDNA sequences	988,169	99.05%	1,159,837	98.6%	1,188,050	99.19%	1,329,601	99%

Table 3: Statistics of pretreatments for EBI and ASaiM on both samples (SRR072233 and SRR072233)

control and dereplication than *EBI metagenomics* does (< 87 %, Table 3). These differences may come from the difference of minimal length sequence. In *EBI metagenomics* pipeline, sequences with less than 100 nucleotides are removed, while in ASaiM the threshold is fixed to 60 nucleotides. More sequences are then conserved with ASaiM. However, this threshold difference explain only small part of observed difference in sequence number after quality control and dereplication. Indeed, if quality control in ASaiM framework is run with same length threshold as in *EBI metagenomics* pipeline, more sequences are eliminated (7.4% and 5.9%) than with standard length threshold (Table 3). These proportions remain lower than the one observed with *EBI metagenomics* pipeline (Table 3). Sequence number differences after quality control and dereplication are then induced moderately by smaller length thresholds in ASaiM. Main sequence number difference are more probably induced by the different used tools, their underlying algorithms and implementations.

In both datasets and with both workflows, few rDNA sequences are found in datasets (Table 3). These datasets are composed of whole genome metagenomic sequences. Few copies of rDNA genes are present in organisms (bacteria, archeae or eukaryotes) and are then expected in metagenomic sequences, as observed for the datasets. Nevertheless, higher proportions of rDNA sequences are found with ASaiM framework (1-1.4%) than with *EBI metagonomics* (0.8-0.9%, Table 3). In *EBI metagenomics* pipeline (Figure 2) *rRNASelector* (Lee, Yi, and Chun 2011) is used to select rDNA bacterial and archaeal sequences. In ASaiM framework, sequences are sorted using *SortMeRNA* (Kopylova, Noé, and Touzet 2012) and databases with bacteria, archaea and also eukaryotes rDNA sequences. Differences of rDNA reference databases, particularly the use of eukaryotic database in ASaiM, may then explain the differences in rDNA sequence proportions extracted by both workflows. In ASaiM framework, 0.03-0.05% of all sequences are matched against databases dedicated to eukaryotic rDNA sequences, but this small proportion does however not explain the whole difference of rDNA sequence proportion between *EBI metagenomics* and ASaiM framework. The rDNA sequences found with *EBI metagenomics* correspond to a subset of rDNA sequences found with ASaiM: more than 97% of rDNA sequences found with *EBI metagenomics* are similarly also found as rDNA sequences with ASaiM framework, less than 2.5% of rDNA sequences found with *EBI metagenomics* are identified as non rDNA sequences with ASaiM framework, the other sequences (<60) may correspond to sequences differentially filtered or trimmed during quality control. *EBI metagenomics* and ASaiM pipelines extract then similar rDNA sequences and these sequences corresponds to sequences from expected organisms. A high proportion (80-86%) of rDNA sequences found with ASaiM and its general rDNA databases are also found with SortMeRNA and databases constitued only of rRNA sequences from expected organisms, and 98.8-99.3% of rDNA sequences found with expected organism rRNA sequence databases are found with ASaiM general databases. Hence, sequence sorting in ASaiM extracts rRNA/rDNA sequences close to rDNA sequences of

expected organisms.

3.2 Taxonomic analyses

Both metagenomic datasets come from a genomic mixture of 22 known microbial strains whose abundance is known (based on mapping on reference genomes, Figure 4). The expected community structures inside the datasets are then known. This information can then be used to analyze ASaiM framework taxonomic results and compare them to *EBI metagenomics* pipeline taxonomic results.

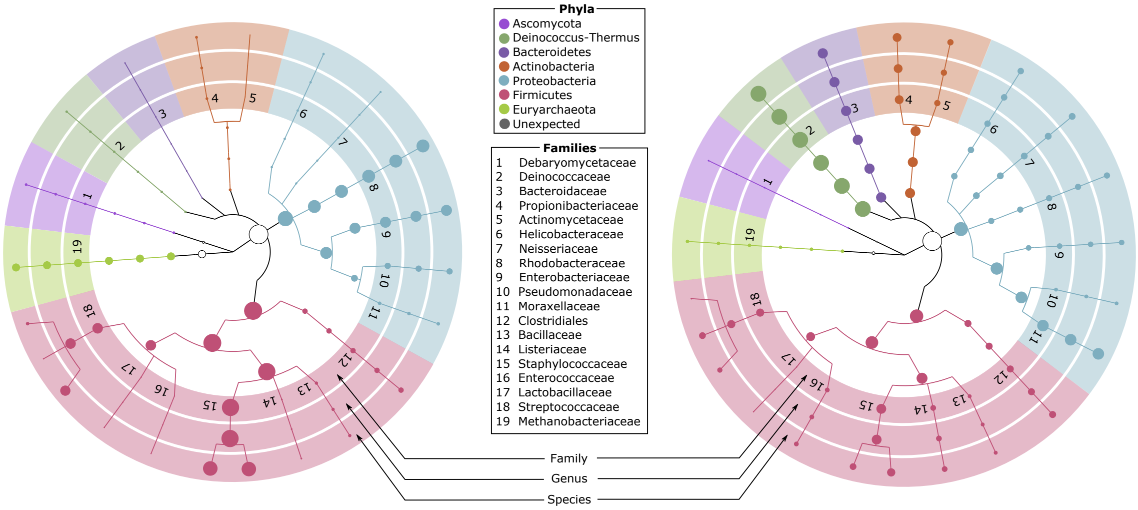

Figure 4: Expected taxonomy for SRR072232 (left) and SRR072233 (right) from domains to species. Circle diameters at each taxonomic levels are proportional to mapping-based relative abundance of corresponding taxon.

3.2.1 ASaiM taxonomic results

ASaiM workflow uses *MetaPhlAN* (2.2.5) (Truong et al. 2015; Segata et al. 2012) for taxonomic analyses. *MetaPhlAn* profiles the microbial community structure using a database of unique clade-specific marker genes identified from 17,000 reference genomes. *MetaPhlAn* runs fast within ASaiM framework: less than 10 minutes to assign taxonomy on > 1,100,000 sequences (Table 2).

MetaPhlAn generates a plain text file with relative abundance of clades at different taxonomic levels. To visualize *MetaPhlAn* results, *Krona* (Ondov, Bergman, and Phillippy 2011) generates interactive representations of taxonomic assignation and *GraPhlan* for static representations. Original static representations are modified (*e.g.* colors, legend) to help comparison with expected taxonomy (Figure 5).

Same species are expected in both dataset, but the taxonomic diversity in SRR072232 dataset is reduced compared to the one in SRR072233 dataset (Figure 5) with less taxons found at each

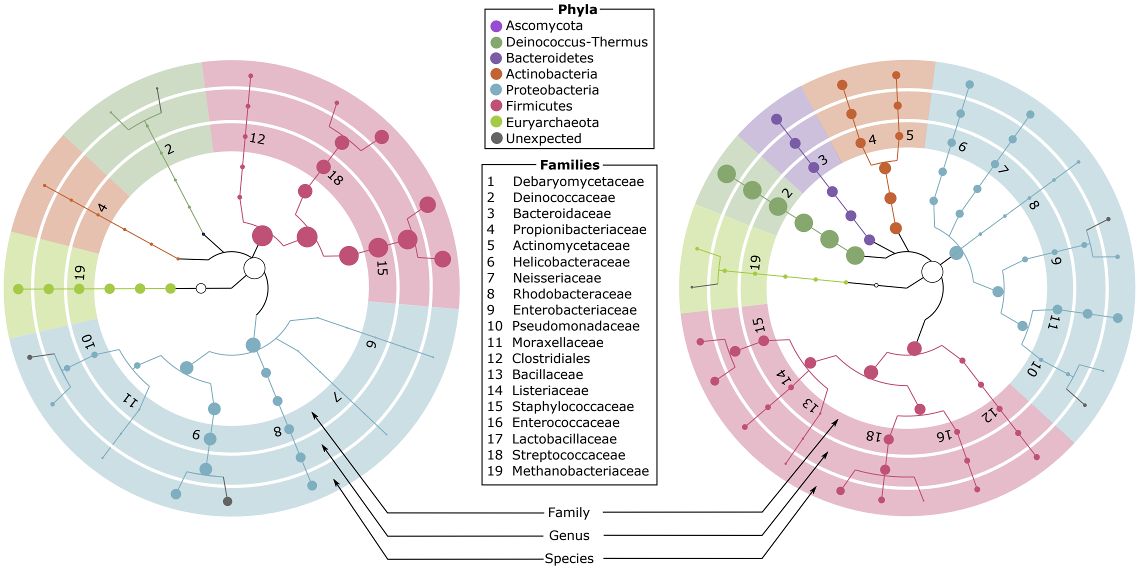

Figure 5: Taxonomy for SRR072232 (left) and SRR072233 (right) from domains to species, found with ASaiM framework. Circle diameters at each taxonomic levels are proportional to relative abundance of corresponding taxon. Colors and family numbers are the same as the ones used in Figure 4. Gray circles and lines represent unexpected lineages.

taxonomic levels. 17 and 20 of the 22 expected species are found for SRR072232 and SRR072233 respectively (Figure 6).

The expected species *Candida albicans* is missing in both dataset results, because of the used *MetaPhlAn2* database. The *MetaPhlAn2* database is built on ~17,000 reference genomes, but only ~110 eukaryotic reference genomes and no *Candida albicans*. The phylogenetic markers for this species are missing: even on a dataset with only sequences extracted from *Candida albicans* reference genomes, this species is not found with *MetaPhlAn2*.

Other missing species (*e.g.* *Enterococcus faecalis* or *Lactobacillus gasseri*) correspond to under-represented species, *i.e.* species whose few sequences are found using mapping (Figure 6). The phylogenetic signal is then too low to detect these species using *MetaPhlAn2*. Indeed, for both datasets, ASaiM framework can not detect any species with mapping-based relative abundance smaller than 0.1% (Figure 6).

One species with mapping-based relative abundance close to 0.1% is not found for SRR072232 dataset but for SRR072233 dataset: *Bacillus cereus thuringiensis*. Few phylogenetic markers for this species are found in *MetaPhlAn2* database. Indeed, on dataset composed only of *Bacillus cereus thuringiensis* reference genome's sequences, phylogenetic markers for *Bacillus cereus thuringiensis* are found, but on a very low percentage of sequences (0.14% of sequences against 2.28% on average for other expected species). The phylogenetic signal are be then to be highest than a certain threshold to detect this species inside a whole metagenomic sequence dataset.

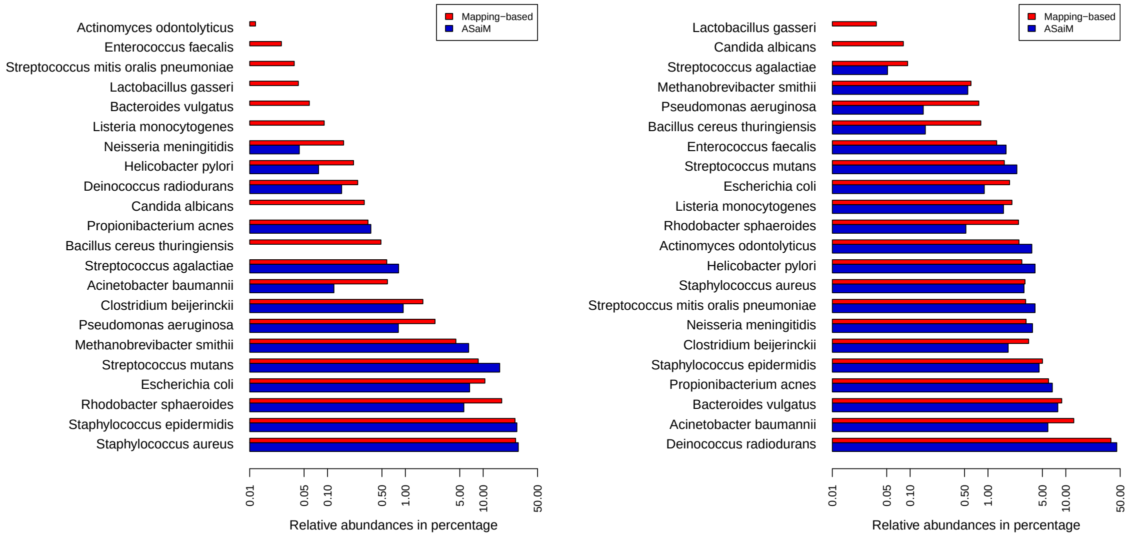

Figure 6: Relative abundances (percentage in log scale) of expected species for SRR072232 (left) and SRR072232 (right) with comparison between expected abundances (based on mapping counts, red thin bars) and abundances obtained with ASaiM (blue wide bars)

3.2.2 Comparison of ASaiM taxonomic results with EBI metagenomics taxonomic results

We can now compare ASaiM taxonomic results to *EBI metagenomics* ones.

EBI metagenomics pipeline uses *QIIME* 1.5.0 (Caporaso et al. 2010) to identify Operational Taxonomic Units (OTUs) and taxonomic assignation for these OTUs. *EBI metagenomics* focuses on taxonomic assignations of 16S sequences. In ASaiM framework, *MetaPhlAn* is executed on quality treated sequences, before any sorting step. Diverse phylogenetic markers are searched on all sequence types (rDNA, non rDNA), not only 16S ones as *QIIME* does. In both datasets, 16S sequences represent a low proportion of sequences (Table 3). Taxonomic assignations of *EBI metagenomics* rely then on less sequences than the ones of ASaiM framework: they are less statistically supported.

The taxonomic assignations in *EBI metagenomics* go from kingdom to family (Figure 7), while in ASaiM framework *MetaPhlAn* outputs taxonomic assignations from kingdom to species (Figure 5). In ASaiM framework, the taxonomic assignations are more precise and more statistically supported than in *EBI metagenomics*.

In both *EBI metagenomics* and ASaiM framework, we observe taxonomic assignations which are unexpected (Table 4, Figures 5 and 7). While only species are found as unexpected in ASaiM framework, *EBI metagenomics* founds unexpected classes, orders and families (Table 4, Figure 7). For ASaiM framework, 4 species in each sample are identified as “unclassified” (Table 4): they are affiliated to the correct genus but not to correct species. Corresponding sequences may be highly similar in several species from the same genus and can not then be assigned to a specific species. Taxonomic assignations with *MetaPhlAN* are then more accurate (less unexpected clades and for a

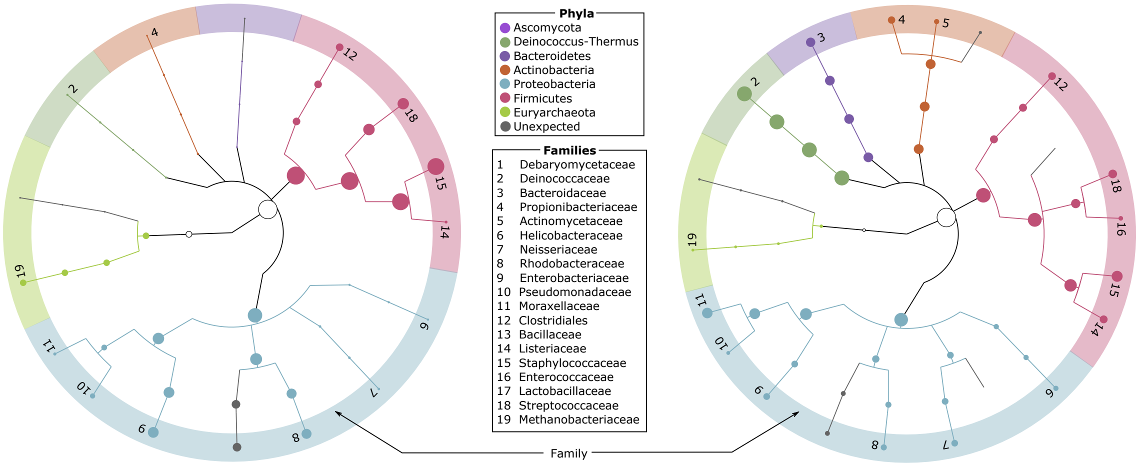

Figure 7: Taxonomy for SRR072232 (left) and SRR072233 (right) from domains to families, found with EBI metagenomics pipeline. Circle diameters at each taxonomic levels are proportional to relative abundance of corresponding taxon. Colors and family numbers are the same as the ones used in Figure 4. Gray circles and lines represent unexpected lineages.

higher level).

		SRR072232		SRR072233	
Taxonomic level	Clade	EBI	ASaiM	EBI	ASaiM
Class					
	Methanopyri	0.09%	-	0.21%	-
Order					
	Rickettsiales	5.71%	-	1.43%	-
	Methanopyrales	0.09%	-	0.21%	-
Family					
	Rickettsiales mitochondria	5.71%	-	1.43%	-
	Methanopyraceae	0.09%	-	0.21%	-
	Paraprevotellaceae	-	-	0.09%	-
	Cryptosporangiaceae	-	-	0.5%	-
Genus		NA	-	NA	-
Species		NA		NA	
	Escherichia unclassified	NA	4.85%	NA	0.8%
	Pseudomonas unclassified	NA	1.12%	NA	0.56%
	Methanobrevibacter unclassified	NA	-	NA	0.24%
	Deinococcus unclassified	NA	0.16%	NA	-

Table 4: Relative abundances of unexpected clades at different taxonomic levels in taxonomic results of *EBI metagenomics* and ASaiM framework for both samples (SRR072233 and SRR072233). NA stands for "Not Applicable"

As mentioned before, the most precise taxonomic level for *EBI metagenomics* is family (Figure 7). The further comparisons between *EBI metagenomics* and ASaiM framework results are then focused

on this level (Figure 8).

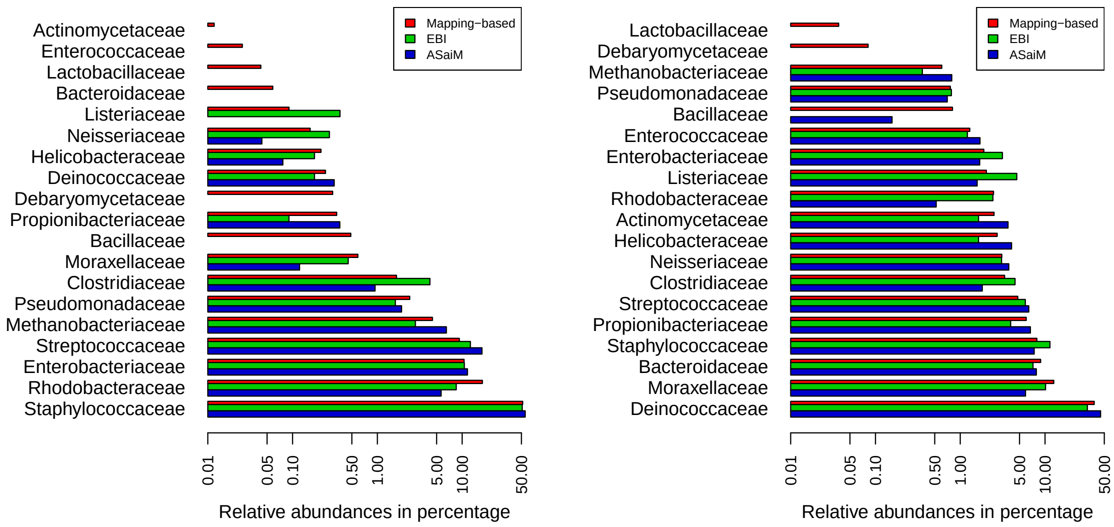

Figure 8: Relative abundances (percentage, log scale) of expected families for SRR072232 (left) and SRR072233 (right) with comparison between mapping-based relative abundances (red thin bars), abundances obtained with *EBI metagenomics* (green wide bars) and abundances obtained with ASaiM (blue wide bars).

Neither ASaiM nor *EBI metagenomics* found any low abundance families (mapping-based abundance smaller than 0.1%, Figure 8), similarly to the previous observations on raw ASaiM results. Some families with higher abundances are not found too:

- Listeriaceae family

  The expected abundance for this family is close to the 0.1% threshold. This family is detected with *EBI metagenomics* but not with ASaiM. *EBI metagenomics* may be then better to detect families with expected low abundance than ASaiM, at least for Listeriaceae family (Figure 8).

- Bacillaceae and Debaryomycetaceae families

  Both of these families are not found with *EBI metagenomics* for both datasets (Figure 8), despite mapping-based abundance higher than 0.1%. Bacillaceae and Debaryomycetaceae correspond to the family level of *Bacillus cereus thuringiensis* and *Candida albicans* species, respectively. Both species are either not found or hardly found with ASaiM (Figure 6): few phylogenetic markers for these species in *MetaPhlAn2* database. Similarly, the used databases in *EBI metagenomics* may be incomplete regarding phylogenetic markers for the missing families.

Despite fewer sequences used for the taxonomic assignations, *EBI metagenomics* seems better to detect low abundance families than ASaiM framework.

More generally, variations in observed abundances for *EBI metagenomics* or ASaiM framework

		SRR072232			SRR072233		
		Expected	EBI	ASaiM	Expected	EBI	ASaiM
Family	Expected	-	0.101	0.146	-	0.132	0.133
	EBI		-	0.111		-	0.213
	ASaiM			-			-
Species	Expected	-	1	0.178	-	1	0.140
	EBI		-	1		-	1
	ASaiM			-			-

Table 5: Bray-Curtis dissimilarity scores on relative abundances of families and species for both samples (SRR072233 and SRR072233)

correspond to variations in mapping-based abundances (Figure 8). For a broader comparison, Bray-Curtis dissimilarity scores are computed on relative abundances of families (Table 5). With scores close to 0, the communities based family compositions are then similar for mapping, *EBI metagenomics* or ASaiM framework results. Bray-Curtis dissimilarity scores are close but higher than 0, small differences are then observed between the different tools. *EBI metagenomics* results on family relative abundances are closer to expected abundances than ASaiM framework results (Table 5). But the differences are small, particularly for SRR072233 datasets, and the scores remain close to 0 (Table 5).

For species, the observations are different (Table 5). No information is available on species composition with *EBI metagenomics* and dissimilarity scores are then equal to 1. With ASaiM framework, dissimilarity scores are slightly higher for species than for families but they remain close to 0. Thus the ASaiM framework is almost as good as the EBI metagenomic pipeline at family level, but contrary to EBI metagenomic pipeline ASaiM performs very well at the species level when compared to mapping-based communities.

ASaiM framework gives taxonomic results which are accurate, complete, precise and statistically supported. Moreover, the community structure found with the ASaiM framework is close to the expected community structure of the mock community.

3.3 Functional analyses

Contrary to taxonomic results, no expected results are available as a framework to help comparison.

3.3.1 ASaiM functional results

ASaiM framework uses *HUMAnN*2 (Abubucker et al. 2012) for functional analyses. This tool profiles presence/absence and abundance of UniRef50 gene families (Suzek et al. 2015) and MetaCyc pathways (Caspi et al. 2014). The metabolic profile of a microbial community is described in three outputs: abundances of UniRef50 gene families, coverage and abundance of MetaCyc pathways.

More than 50,000 UniRef50 gene families and 480 MetaCyc pathways (Table 6) are reconstructed from > 1,100,000 non rDNA sequences of both samples (Table 3).

	UniRef50 gene families		MetaCyc pathways	
	SRR072232	SRR072233	SRR072232	SRR072233
Number	50,700	69,357	473	481
Similar	26,354		466	
% of similar inside all	51.98%	39%	98.52%	96.88%
Relative abundance (%)	91.78%	63.76%	99.98%	99.94%

Table 6: Global information about UniRef50 gene families and MetaCyc pathways obtained with *HUMAnN2* for both samples (SRR072233 and SRR072233). For each characteristics (gene families and pathways), several information is extracted: all number, number percentage and relative abundance (%) of similar characteristics.

Both datasets come from a genomic mixture of 22 identical microbial strains (Table 1). Same species of both datasets are implied in same metabolic functions. The same metabolic functions are then supposed to be found in both datasets, but with different abundances in both datasets (Table 1),

The sets of gene families are slightly different between both datasets: $< 52\%$ identical gene families (26,354) are found in both samples (Table 6). These identical gene families correspond to the most abundant ones ($> 63\%$ of relative abundance of gene families for each dataset, Table 6). The non similar gene families may correspond to gene families which are in low abundance, are differentially or partially sequenced or made by species which are in small abundance.

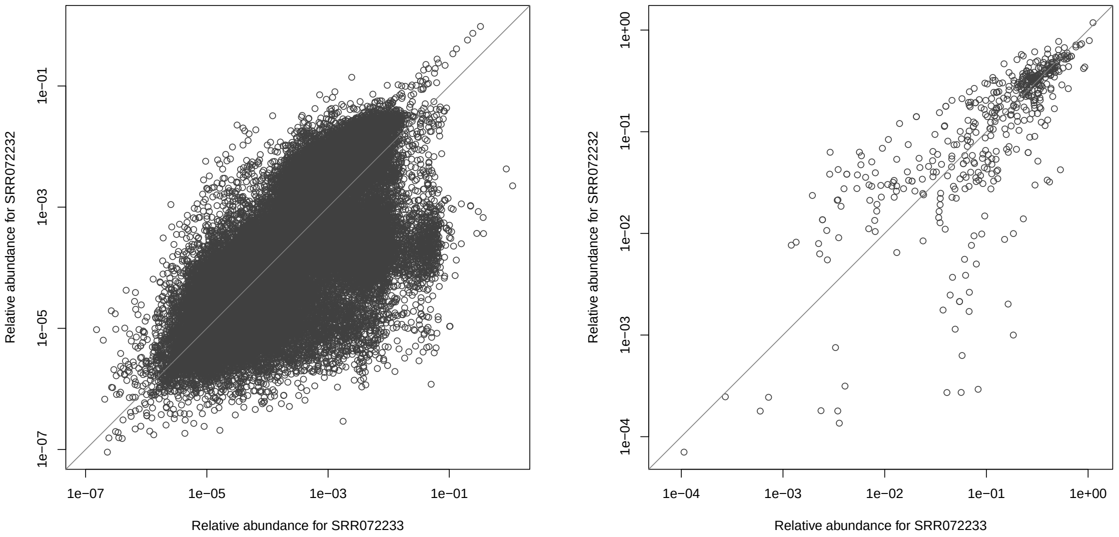

Figure 9: Normalized relative abundances (%) for similar UniRef50 gene families (left) and MetaCyc pathways (right) for both samples (SRR072233 and SRR072233). The relative abundances of each similar characteristics (gene families or pathways) is computed with *HUMAnN2* and normalized by the sum of relative abundance for all similar characteristics.

Global metabolism information in pathways are highly similar in both datasets: $> 96\%$ of similar pathways representing $> 99.9\%$ of overall abundance (Table 6). A pathway is identified if a high

proportion of gene families involved in this pathway is found. Not all involved gene families are then needed to identify a pathway. The impact on metagenomic sequencing are then reduced and similar pathway sets are then found in both datasets.

UniRef50 gene families and MetaCyc pathways are somehow too specific to obtain a broad overview of the metabolic processes. In ASaiM framework, UniRef50 gene families and their abundances are grouped into Gene Ontology (GO) slim terms (Figure 10).

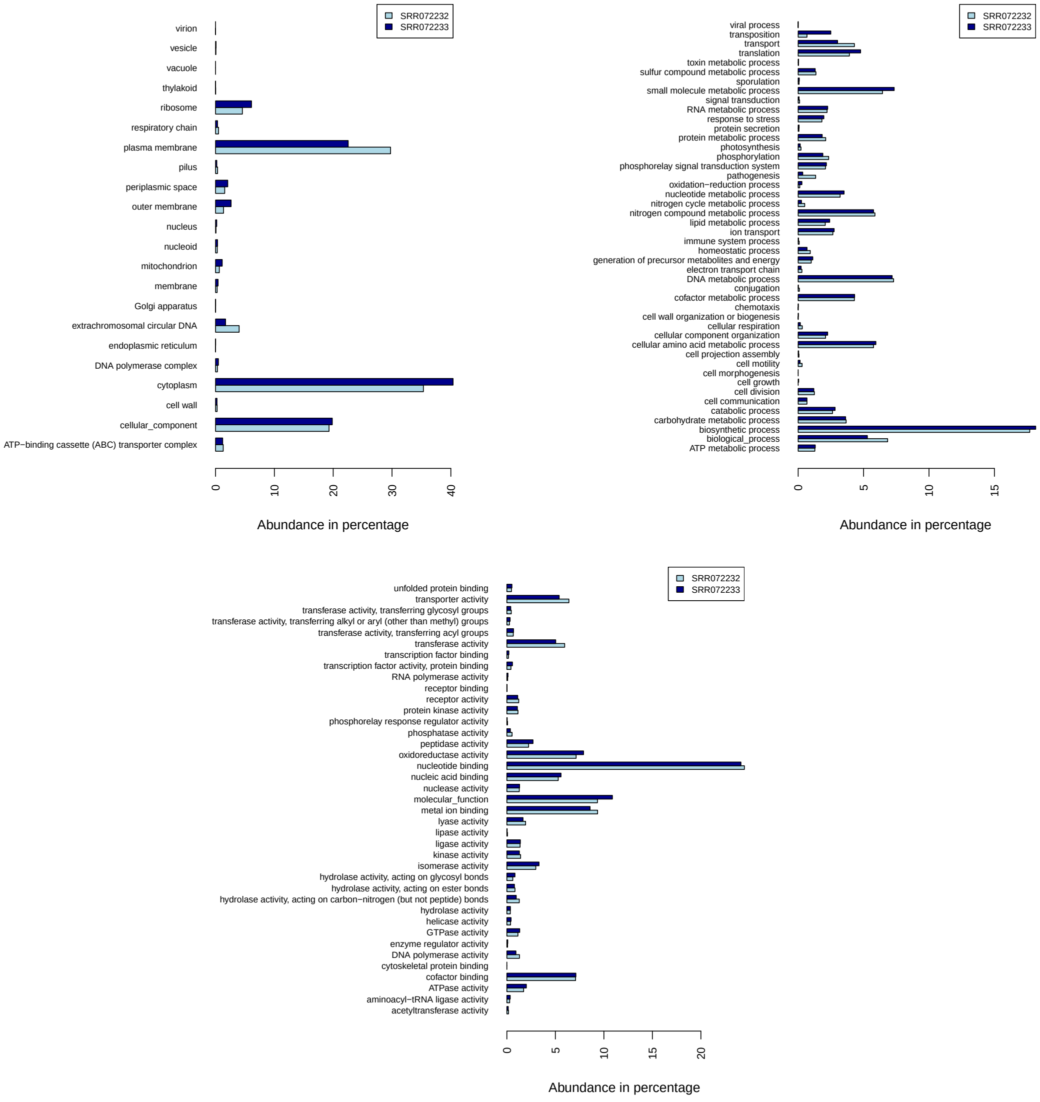

Figure 10: Relative abundances of GO slim terms in SRR072232 and SRR072233 for cellular components (top left), biological processes (top right) and molecular function (bottom)

The abundances of identical metabolic functions are different (Figure 9), as expected. The differential abundance of species involved in function metabolization lead to differential abundance of these functions.

Both communities (with same expected strains but in different abundances) have different metabolic profiles: similar metabolic functions but in different abundances, as expected.

3.3.2 Comparison of ASaiM functional results with *EBI metagenomics* results

In ASaiM framework, *HUMAnN2* computes UniRef50 gene families and their abundances. In *EBI metagenomics* pipeline (Figure 2), functional analyses are based on InterPro database. We can not directly compare these functional results. As in ASaiM framework, *EBI metagenomics* pipeline groups InterPro proteins into Gene Ontology slim terms.

Barplot representations of GO slim term abundances for both samples and both workflows can be difficult to interpret (*e.g.* for cellular components, Figure 11). We compute then the Bray-Curtis dissimilarity scores on normalized relative abundance of GO slim term abundance inside each category (Table 7).

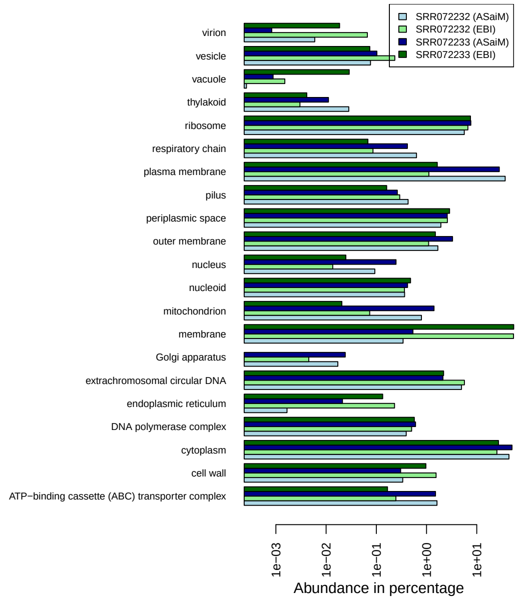

Figure 11: Barplot representation (logarithm scale) of the normalized relative abundances (in percentage) of the cellular component GO slim terms for both samples (SRR072233 and SRR072233) and both workflows (*EBI metagenomics* and ASaiM). The relative abundances of each GO slim terms is normalized by the sum of relative abundance for the found cellular component GO slim terms in both samples and with both workflows.

			SRR072232		SRR072233	
			EBI	ASaiM	EBI	ASaiM
Biological processes	SRR072232	EBI	-	0.319	0.041	0.332
		ASaiM		-	0.327	0.053
	SRR072233	EBI			-	0.338
		ASaiM				-
Cellular components	SRR072232	EBI	-	0.578	0.047	0.587
		ASaiM		-	0.580	0.121
	SRR072233	EBI			-	0.552
		ASaiM				-
Molecular functions	SRR072232	EBI	-	0.309	0.036	0.311
		ASaiM		-	0.307	0.042
	SRR072233	EBI			-	0.305
		ASaiM				-

Table 7: Bray-Curtis dissimilarity scores on relative abundances of families and species for both samples (SRR072232 and SRR072233)

Inside each category, compositions are more similar (dissimilarity scores closer to 0) for both samples analyzed with the same method (*EBI metagenomics* or ASaiM framework) than for same sample analyzed with different methods (*e.g.* SRR072232 analyzed *EBI metagenomics* and ASaiM framework). These composition differences between *EBI metagenomics* and ASaiM framework may come from the different tools, the different databases (InterPro for *EBI metagenomics*, UniRef50 for ASaiM framework) and their way to be grouped into GO slim terms.

3.4 Taxonomically-related functional results

HUMAnN2 stratifies the abundances of gene families and pathways at the community level. Around 35% of gene families (> 90% of relative abundance) and > 80% pathways (> 50% of relative abundance) can be then related to the community structure (species and their abundance, Table 8). We can exploit this information to relate functional results to taxonomic results and answer questions such as “Which taxa contribute to which metabolic functions? And, in which proportion?”.

For both samples, we observe a significant correlation between CDS number in the species and number of gene families found for these species (Table 9). The correlation is significant (*p-value* $< 5.09 \cdot 10^{-3}$) but it is yet not perfect ($r^2 < 0.71$). Gene families can not be then directly mapped to CDS (*e.g.* to obtain expected results).

The relative abundances of the gene families and the pathways are highly correlated to the observed relative abundance of the involved species (Table 9). The sequences of an abundant species in a community are supposed to be abundant in the metagenomic sequences of the community. This relation holds for all sequences, particularly sequences corresponding to gene families. For pathways, the relation is more tricky: a pathway is identified if most of the gene families that constitute them are found. The abundance of a pathway is proportional to the number of complete “copies” of this pathway in the species. Then, a pathway is abundant if its parts are all found in numerous copies, leading to a tricky relation between species abundance and pathway abundance. But, the high correlations between species relative abundance and mean relative pathway abundance (Figure

	UniRef50 gene families		MetaCyc pathways	
	SRR072232	SRR072233	SRR072232	SRR072233
Number	26,219	41,005	402	400
% of associated to a species inside all	26.60%	31.62%	82.56%	80%
Relative abundance (%)	93.40%	90.24%	61.08%	51.52%
Identical characteristics	19,815		363	
% of identical characteristics inside characteristics associated to a species	68.02%	48.32%	90.30%	90.75%
Relative abundance of identical characteristics inside characteristics associated to a species (%)	89.17%	44.75%	91.87%	42.70%

Table 8: Global information about UniRef50 gene families and MetaCyc pathways related to species for both samples (SRR072233 and SRR072233). For each characteristics (gene families and pathways), several information is extracted: all number, number percentage and relative abundance (%) of identical characteristics and *p-value* of Wilcoxon test on relative abundance normalized by the sum of relative abundance for all identical characteristics.

		UniRef50 gene families		MetaCyc pathways	
		SRR072232	SRR072233	SRR072232	SRR072233
Number					
Correlation with species CDS number	r^2	0.71	0.60		
	p-value	$4.67 \cdot 10^{-3}$	$5.09 \cdot 10^{-3}$		
Mean abundance (Figure 12)					
Correlation with species abundance	r^2	0.95	0.98	0.90	0.93
	p-value	$1.51 \cdot 10^{-7}$	$2.9 \cdot 10^{-13}$	$1.91 \cdot 10^{-7}$	$5.88 \cdot 10-12$
Difference of mean abundance					
Correlation with species abundance difference	r^2	0.89		0.84	
	p-value	$4.12 \cdot 10^{-7}$		$4.65 \cdot 10^{-6}$	

Table 9: Correlation coefficients and p-values (Pearson's test) for UniRef50 gene families and MetaCyc pathways related to species for both samples (SRR072233 and SRR072233). CDS number for each strain has been extracted from GenBank given the links in Table 1

12, Table 9) confirm good pathway reconstructions in our datasets, particularly for the abundant species. To confirm the previous observations and conclusion, we also observe a strong and significant correlation between species abundance difference and difference of gene family and pathway mean abundance between both samples (Figure 12, Table 9).

Hence, ASaiM framework approach based on *MetaPhlAn2* and *HUMAnN2* results gives accurate and relevant taxonomically-related functional results.

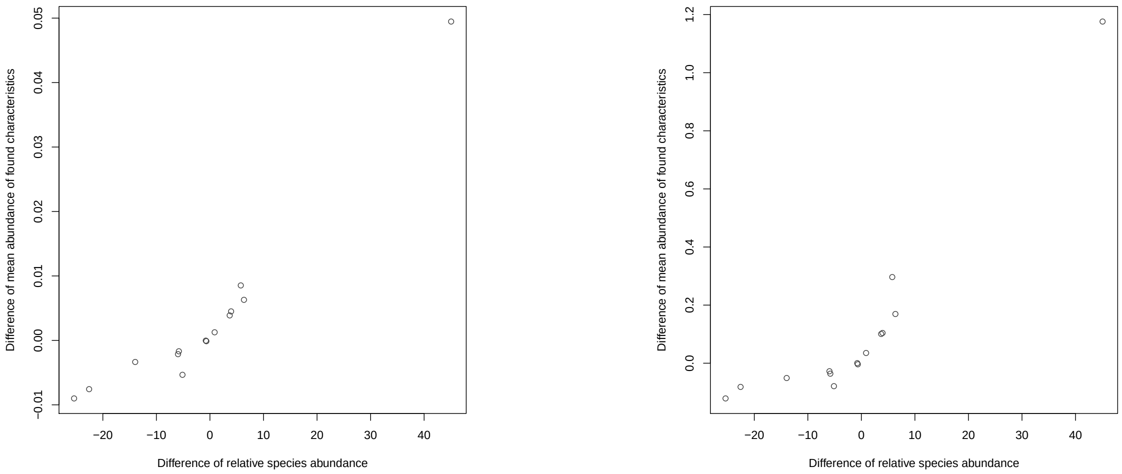

Figure 12: Difference in mean abundances for gene families (left) and pathways (right) in function of difference of related species abundance between both samples. Correlation coefficients and p-values are detailed in Table 9

4 Conclusion

ASaiM framework quickly analyses a raw metagenomic dataset (in few hours in a commodity computer). Taxonomic analysis using *MetaPhlAn2* gives a great insight of the community structure with complete, accurate and statistically supported information. *HUMAnN2* and extraction of GO slim terms give a broad overview of metabolic profile of studied microbial community. Furthermore, this metabolic profile can be related to the community structure to obtain information such as which species might be involved in which metabolic function. This relation between function and taxonomy is specific to the ASaiM framework and not available with solutions such as *EBI metagenomics* pipeline.

Based on Galaxy, ASaiM framework has all Galaxy's strength: accessibility, reproducibility and modularity. Numerous intermediary results can also be accessed during or after workflow execution, allowing deep investigation of taxonomic and functional analyses of microbial communities. The numerous tools and the workflows make ASaiM a powerful framework to analyze microbiota from shotgun raw sequence data and give a global overview of the community structure, its functionnal capabilities and potential links between community structure and biological functions.

References

Abubucker, Sahar, Nicola Segata, Johannes Goll, Alyxandria M. Schubert, Jacques Izard, Brandi L. Cantarel, Beltran Rodriguez-Mueller, et al. 2012. "Metabolic Reconstruction for Metagenomic Data and Its Application to the Human Microbiome." *PLoS Comput Biol* 8 (6): e1002358.

doi:10.1371/journal.pcbi.1002358.

Camacho, Christiam, George Coulouris, Vahram Avagyan, Ning Ma, Jason Papadopoulos, Kevin Bealer, and Thomas L. Madden. 2009. "BLAST +: Architecture and Applications." *BMC Bioinformatics* 10 (1): 421. doi:10.1186/1471-2105-10-421.

Caporaso, J. Gregory, Justin Kuczynski, Jesse Stombaugh, Kyle Bittinger, Frederic D. Bushman, Elizabeth K. Costello, Noah Fierer, et al. 2010. "QIIME Allows Analysis of High-Throughput Community Sequencing Data." *Nature Methods* 7 (5): 335–36. doi:10.1038/nmeth.f.303.

Caspi, Ron, Tomer Altman, Richard Billington, Kate Dreher, Hartmut Foerster, Carol A. Fulcher, Timothy A. Holland, et al. 2014. "The MetaCyc Database of Metabolic Pathways and Enzymes and the BioCyc Collection of Pathway/Genome Databases." *Nucleic Acids Research* 42 (D1): D459–D471. doi:10.1093/nar/gkt1103.

Hunter, Sarah, Matthew Corbett, Hubert Denise, Matthew Fraser, Alejandra Gonzalez-Beltran, Christopher Hunter, Philip Jones, et al. 2014. "EBI Metagenomics—a New Resource for the Analysis and Archiving of Metagenomic Data." *Nucleic Acids Research* 42 (D1): D600–D606. doi:10.1093/nar/gkt961.

Kopylova, Evguenia, Laurent Noé, and Hélène Touzet. 2012. "SortMeRNA: Fast and Accurate Filtering of Ribosomal RNAs in Metatranscriptomic Data." *Bioinformatics (Oxford, England)* 28 (24): 3211–7. doi:10.1093/bioinformatics/bts611.

Lee, Jae-Hak, Hana Yi, and Jongsik Chun. 2011. "rRNASelector: A Computer Program for Selecting Ribosomal RNA Encoding Sequences from Metagenomic and Metatranscriptomic Shotgun Libraries." *The Journal of Microbiology* 49 (4): 689–91. doi:10.1007/s12275-011-1213-z.

Li, Heng, and Richard Durbin. 2009. "Fast and Accurate Short Read Alignment with Burrows-Wheeler Transform." *Bioinformatics (Oxford, England)* 25 (14): 1754–60. doi:10.1093/bioinformatics/btp324.

———. 2010. "Fast and Accurate Long-Read Alignment with Burrows–Wheeler Transform." *Bioinformatics* 26 (5): 589–95. doi:10.1093/bioinformatics/btp698.

Ondov, Brian D., Nicholas H. Bergman, and Adam M. Phillippy. 2011. "Interactive Metagenomic Visualization in a Web Browser." *BMC Bioinformatics* 12 (1): 385. doi:10.1186/1471-2105-12-385.

Segata, Nicola, Levi Waldron, Annalisa Ballarini, Vagheesh Narasimhan, Olivier Jousson, and Curtis Huttenhower. 2012. "Metagenomic Microbial Community Profiling Using Unique Clade-Specific Marker Genes." *Nature Methods* 9 (8): 811–14. doi:10.1038/nmeth.2066.

Suzek, Baris E., Yuqi Wang, Hongzhan Huang, Peter B. McGarvey, Cathy H. Wu, and the UniProt Consortium. 2015. "UniRef Clusters: A Comprehensive and Scalable Alternative for Improving Sequence Similarity Searches." *Bioinformatics* 31 (6): 926–32. doi:10.1093/bioinformatics/btu739.

Truong, Duy Tin, Eric A. Franzosa, Timothy L. Tickle, Matthias Scholz, George Weingart, Edoardo Pasolli, Adrian Tett, Curtis Huttenhower, and Nicola Segata. 2015. "MetaPhlAn2 for Enhanced Metagenomic Taxonomic Profiling." *Nature Methods* 12 (10): 902–3. doi:10.1038/nmeth.3589.
