# Supplementary material for: ASaiM: a Galaxy-based framework to analyze microbiota data
Source: Gigascience. 2018 May 15;7(6):giy057. doi: 10.1093/gigascience/giy057 (PMC6007547; doi:10.1093/gigascience/giy057)
Supplement: Response_to_Reviewer_Comments_Original_Submission.pdf [file giy057_response_to_reviewer_comments_original_submission.pdf]

Author's Response To Reviewer Comments

Close

We thanked the editor and the reviewers for their suggestions and constructive critiques.

Editor

“Your manuscript "ASaiM: a Galaxy-based framework to analyze raw shotgun data from microbiota" (GIGA-D-17-00230) has been assessed by our reviewers. Although it is certainly of interest, we are unable to consider it for publication without some revisions. The reviewers have raised a number of points which we believe would improve the manuscript and may allow a revised version to be published in GigaScience. Particularly there are some suggestions to change the focus, so you will need to decide to focus purely on the shotgun sequencing, or take a broader approach and potentially change it to a more general toolkit (potentially also stressing the educative aspects too).”

We think that ASaiM should be general toolkit for the analysis of microbiota data. Indeed, it is currently used for diverse metagenomics projects (either shotgun or amplicon), like the Beer DeCoded project which analyzes the ITS sequences of the beer microbiota in a pedagogic way or the assembly of metagenomics datasets from EBI Metagenomics to extract CRISPR subtypes. So, we added some tools and workflows for ITS analysis and metagenomic assembly and are currently working on integration of binning tools. We also changed the title to indicate the general purpose of ASaiM: “ASaiM: a Galaxy-based framework to analyze microbiota data”.
To stress the educative aspects, we also added a short paragraph explaining in more detail how ASaiM is used for a citizen science project (Beer DeCoded) or in training courses, for example to understand and use the EBI metagenomic workbench in a reproducible way for teaching undergrads.

“In addition, we are now asking authors to register any new software application or pipeline in the SciCrunch.org database to receive a RRID (Research Resource Identification Initiative ID) number, and include this in your manuscript. This will facilitate tracking, reproducibility and re-use of your tool.”

The tool has been submitted to SciCrunch: RRID:SCR_015878. We added the information in the manuscript.

Reviewer #1

“Excellent paper, useful collections of tools, focused approach and well organized with great documentation.”

We thank the reviewer for this nice comment.

“Enough background for a software paper, my suggestion would be if you can mention a little more on metagenomics pipelines available on the main Galaxy server, in addition to an example of

specialized Galaxy servers for metagenomics - for example the Metaphlan group they have such a specialized server: https://huttenhower.sph.harvard.edu/galaxy/"

We added in the introduction a sentence about the main Galaxy server and the metagenomics tools available there. We also mentioned the server of the Huttenhower lab. Moreover, we are in contact with the administrators of usegalaxy.org and we will ensure that all workflows and trainings will also work on their server.

"In addition I was really excited to see the provenance mentioned. Since the documentation is so extensive (and excellent!), perhaps the authors could add a section on how to save a docker container where data has been processed with their tool (also how to bundle the volumes with the data), so that the whole package and be distributed (and provide analysis provenance), to collaborators, with a publication etc."

We tried to keep the documentation on the Docker usage simple and not redundant with the already extensive documentation available for the Galaxy Docker project (https://github.com/bgruening/docker-galaxy-stable). In the online documentation, we added more links in the documentation to this Docker documentation, especially with the questions the reviewer asked, and added a sentence to refer to this online documentation in the manuscript.
To answer the question directly, it is possible to store, archive and share the entire /export folder of a Galaxy Docker image. This can then be easily shared, uploaded to Zenodo etc. and reused with any other Galaxy Docker container.

"Overall an excellent paper !"

Reviewer #2

"Some spelling errors:

line 56: blocking scientist -> blocking scientists
line 124 an web-interface -> a web-interface
line 135: visualization -> visualizations
line 135: such Phyloviz -> such as Phyloviz
line 157 Figure 2): we -> Figure2) and we
line 175-176: We integrate then also a workflow -> We also integrated them in a workflow

in report (supp. material) targeted abundances may be not reflect -> targeted abundances may not reflect"

Thanks for reporting these mistakes, we addressed all of them in the revised version.

Further remarks:

1) "The title is a bit lacking in context. ASAIM is clearly dedicated only towards the taxonomic and functional analysis of metagenomic data (either from amplicon sequencing or from shotgun sequencing). It would be beneficial for the reader to deduce that from the title."

ASaiM is a community starting point for all people interested in metagenomic research. During the last months other tools related to metagenomic assembly as MetaSPAdes or MEGAHIT and some tools for binning were added, partially by the community, but also on request from collaborators. The objectives of ASaiM is to offer a comprehensive and general workbench for microbiota analysis and thus we would like to have a slightly more general title. However, we changed the title slightly to: “ASaiM: a Galaxy-based framework to analyze microbiota data”

2) “It's not quite clear the innovative part of the platform. Besides collecting all those preexisting tools in an organized manner under Galaxy's umbrella what was the added contribution of ASAIM's team? Did you develop new wrapper/parser scripts for some/all of these tools in order to integrate them with Galaxy? What is the added value of the 3 new tools you developped? The GO slim term tool seems to be one of the final tools (purple) in your workflow (is that correct?). What about the other two for searching EBI and ENA databases? Are they part of one of the workflows or just additional standalone tools?”

The ASaiM team migrated 12 tools/suites of tools and their dependencies to Bioconda (e.g. HUMAnN2, MetaPhlAn2, GraPhlAN), integrated 16 suites (>100 tools) into Galaxy (e.g. HUMAn2 or QIIME with its around forty tools), i.e. developing the wrappers for these tools. We also checked and updated the wrappers of the existing tools. Moreover, several Galaxy datatypes, (interactive) training material and a visualization were developed and integrated into Galaxy.
The 3 tools we developed were needed to close missing steps in workflows or to make it more convenient for users to access publicly available data.
The GO slim tool is used to aggregate the gene family abundances into GO terms and is indeed one of the final steps in the workflow.
The EBISearch and ENASearch tools are standalone tools to allow users to query ENA and EBI Metagenomics databases (data, metadata) and transfer to directly into Galaxy. They are not integrated into the one of our predefined workflows because the inputs of the workflows could be local data or data from external database such as ENA and we can not determine that before.
To complement the tools and workflows, the ASaiM team created also documentation and tutorials.

3) ”The comparison between ASAIM and EBI analysis seems rather trivial. It's not a comparison of the two platforms rather than a comparison of the two different tools they are using (QIIME and Metaphlan). It would make much more sense a comparison between EBI's workflow run in the exact same way as an ASAIM/Galaxy workflow with the same tools.”

We would like to do this, but currently it is not possible to know the exact parameters which are used in the EBI Metagenomics workflow. This latter workflow is, unfortunately, currently a blackbox in contrary to ASaiM whose one of the objectives is to make microbiota research more transparent and reproducible.

4) “The same goes for functional analysis (where you mention comparison is not feasible). You just present results derived from two different methods with no comparable points.”

For the same reason as stated above we are very limited in what we can compare. Moreover. the functional information are extracted with two different types of information. EBI Metagenomics extracts the InterProScan gene families. In ASaiM, we extract with HUMAnN2 the UniRef gene families. It complexifies any comparisons.

5) “In line 200 the command you state
docker run -d -p 8080:80 quay.io/bebatut/asaim

is different than the one stated in your webpage where the installation instructions are:

docker run -d -p 8080:80 quay.io/bebatut/asaim-framework

while the "asaim" command doesn't work (not authorized error) the "asaim-framework" seems to work”

We apologize for this mistake. We fixed the command mentioned in the manuscript to fit to the one in the instructions.

6) “In supplementary material report page 3 contains a table that is not well displayed”

Thanks for reporting this. We fixed the table.

7) “Installation was not succesful so actual testing of the tool was not possible. Installation in a new CentOS distribution (3.10.0-514) under a Virtuabox engine failed. It could be useful to mention in your docs how to install and start the docker engine before attempting to download the ASAIM package especially for those with little or no command line knowledge.”

As the installation of the Docker engine can vary between different operating systems and is changing over time we think the best way is to link to the upstream documentation under https://docs.docker.com/engine/installation. We also added a link to a video explaining how to use Kitematic for Galaxy Docker, for the non-linux users.

“At some point during the installation process there was an error saying:

"failed to register layer: ApplyLayer exit status 1 stdout: stderr: write /tool_deps/_conda/envs/__picrust@1.1.1/lib/python2.7/site-packages/mpi4py/MPI.so: no space left on device."

Not sure how that's possible with 34GB available free space. Does ASAIM include databases that take up more space than that? If that's the case you should probably include that in the Requirements section in your webpage and inform the reviewers as well in order for us to be able to succesfully install and properly test it.”

We apologize for this unfortunate experience.
ASaiM includes numerous tools and reference databases for HUMAnN2 and MetaPhlAn2 and this increases the required disk space to 40GB. We forgot to mention this in our documentation and addressed this issue. In the meantime we are working hard to make this experience easier in the near future. The latest ASaiM Docker release already supports the CVMFS filesystem, with which we can easily mount in TB of reference data into every image. The data is then only downloaded if it get accessed by tools. We will extend this over the next releases.

Reviewer #3

“The manuscript describes an alternative workflow for the processing of shotgun metagenomics and metatranscriptomic data, called ASaiM.
ASaiM integrates multiple tools for the analysis and manipulation of raw metagenomics and metatranscriptomic data, that are available, both as single tools and combined in multiple pipelines, within the Galaxy workflow and with a Docker and conda support. ASaiM comes with a very impressive documentation and it is of high importance in the metagenomics community, where most of the analyses are carried out using in-house scripts that, as pointed out by the authors, hinder reproducibility.

However, several other metagenomics pipelines are already available: MG-RAST and the EBI metagenomics pipeline, that the authors briefly discuss in the Introduction, but also MOCAT2, MetAMOS, and another Galaxy metagenomic pipeline. How does ASaiM compare within this wider ecosystem? MOCAT2, for instance, comes with a set of preset parameters, stored in a single file, that already improve reproducibility, and the EBI metagenomics pipeline clearly shows the software version (e.g., https://www.ebi.ac.uk/metagenomics/pipelines/3.0), allowing provenance.”

Provenance is way more than just the version of the used tool in a workflow. Every single parameter or the version of the used reference database can have a huge influence on the results.
But even if the various webservers would allow for a complete provenance it’s hard or impossible to run those pipelines locally or on a local cluster. ASaiM is changing this by offering all tools of the different pipelines in one workbench, that can be deployed locally, on a cluster or in a cloud. The different pipelines can even be mixed if necessary, allowing for a unmatched flexibility and reproducibility. Moreover, ASaiM will ensure that the entire provenance is tracked and every single parameter, the exact version of the tools and input data is tracked and can be reproduced and compared.
The reviewer mentioned MOCAT2. This command line tool is a great tool. However, it focuses only on metagenomic data (not for metataxonomic or metatranscriptomic data, as we would like) and its command-line use is a limitation for its use for all scientists working with microbiota data. We will work on integrating it into ASaiM.
With EBI Metagenomics, the versions of software are available but not the parameters or the versions of databases used. For this reason, we did not set up any parameters in the workflow developed to reproduce the one on EBI Metagenomics. We think it is a big issue for reproducibility, as the parameters and the databases can have a big impact on the results.

“Also, the authors point out that the main problems in analysing metagenomics data are, first, the selection and configuration of the necessary tools, then the definition of the correct computational resources, and, finally, the definition of a correct analysis workflow. However, in this reviewer's opinion, ASaiM does not fully address these limitations. The authors implement about 25 tools for the processing of metagenomics data but give little explanation on the reasons these specific tools have been selected, or which tools should be used when multiple tools within the same class are available. Novices in the field would surely appreciate these pieces of information as a way to select the correct software for the problem at hand.”

Information about this was added in the documentation and in the tutorials we developed with the Galaxy Training Network. We follow the idea to offer a variety of different tools, even if they have overlapping functionality, to enable a lot of flexibility and freedom in data analysis. In this regard we want to offer easy access to a lot of different software. If a user needs guidance and the amount of tools is just overwhelming, we provide workflows for different use-cases and training material, in which we choose specialised tools and leave other out. However, we think the power of an analysis

should be in the hand of the user and different steps in a workflow should/could be interchangeable.

“Regarding the workflows included in ASaiM, one is a reimplementation of the EBI workflow, one cannot be used for analysing metagenomic shotgun data, and only one is novel (that this reviewer supposes is the one called very generally "ASaiM"). This reviewer would suggest the authors to focus more on describing this novel workflow, and to remove all the references to QIIME and Mothur tools (or to 16S data analysis in general) since these are not able to analyse shotgun metagenomics data and may generate confusion.”

We think that ASaiM should be general toolkit for the analysis of microbiota data, not only for shotgun data. Microbiota analyses are usually not only focused on one type of analysis (metagenomics, metatranscriptomics, metataxonomics). We usually need to combine tools developed for different purposes to analyze our data. For example to compute abundance statistics such as alpha or beta diversity, we can apply the QIIME tools on the BIOM files generated by metagenomics tools such as MetaPhlAn. ASaiM is currently used in diverse microbiota projects (shotgun, amplicon and ITS data). We would like then to keep the mention of the QIIME and Mothur tools, and their workflow. We also added two workflows for metagenomic assembly (one using MEGAHIT and one using MetaSPAdes), including quality control, assembly and assembly checking (statistics, mapping and identification of potential assembly error signatures).

“For instance, it would be interesting to know how the workflow can be customised, whether default parameters are available and how they have been selected, and have more detailed and exhaustive information on time and computational requirements (and not only on two samples).”

We clarified the customization of workflows in the manuscript:
“To assist in microbiota analyses, several default workflows are proposed and documented (tools, default parameters) in ASaiM. These workflows can be used as they are, customized either on the fly to tune the parameters or globally to change the tools, their order and their default parameters, or even used as subworkflows.”.
We added more details in the documentation and also in the tutorials about the choices of default parameters for the tools.
Exhaustive information on time and computational requirements are difficult to extract. They greatly depend on the input data. Currently for the shotgun workflow, the main time-consuming task is HUMAnN2 and its execution time is not linear with input size. We added a sentence in the manuscript to mention that.
In general ASaiM is configured by default to run on normal personal computers, but because ASaiM is utilizing the Galaxy framework all tools and workflows can be easily configured to scale out and use entire clusters or other available compute resources. Here, we are referring to the upstream documentation of Galaxy or the Docker Galaxy project.

“Also, it is not clear what improvements are brought by ASaiM and what are due to the usage of Galaxy (reproducibility, provenance, being user-friendly), or of HUMAnN2 (ability to infer the taxonomic profiles up to the species level, availability of genes and pathways abundances tables). For instance, how the proposed 'functional and taxonomic combination analysis' block differs with that proposed within the HUMAnN2 pipeline?”

ASaiM is a collection of existing tools that are combined into a dedicated Galaxy instance. On top of

these tools we have build workflows and training material. Thanks to Galaxy and Docker, ASaiM can be easily shipped, deployed, but also customized for anyone. The ASaiM team maintains the tools, updates them, integrates new tools (> 100), datatypes and visualizations and develops documentation and training to help researchers to deal with microbiota data. We clarified the manuscript in this direction.
The "functional and taxonomic combination analysis" block is the Galaxy implementation of the HUMAnN2 pipeline, but inside a workflow to help its execution on many samples and after several pre-processing steps (quality control, sorting, MetaPhlAn2), without the need to care about the computational details. It is a turnkey solution.

"More in general, this reviewer's main concern regards the focus of the manuscript. Are the authors interested in presenting the Galaxy implementation of a variety of metagenomics tools? Or to present a novel reproducible pipeline for the analysis of metagenomics data? Are they interested in metagenomics or metagenetics (16S) analysis? In this reviewer's opinion, the manuscript would surely benefit in focusing on a single message, while additional features (such as the analysis of metagenetics data) should be only briefly mentioned."

We are interesting in presenting ASaiM as an environment for people working on any type of microbiota data: a Galaxy implementation including a variety of microbiota related tools, workflow, documentation and training, whch is easy to distribute with its Docker image, for example for a publication of an analysis. We tried to make this message clearer in the manuscript, with for example a slightly different title "ASaiM: a Galaxy-based framework to analyze microbiota data"

"The manuscript includes some imprecision, with several concepts repeated multiple times, and would surely benefit from a proofreading by a native speaker:"

1. "Lines 40-43. Metagenomics and metatranscriptomics techniques do not allow to get insight into metabolic components, but only on the inferred functions of the micro-organisms present in one sample (as done, for instance, by HUMAnN2). To measure the metabolic components, one should use another approach, namely metametabolomics. It is also not clear what 'phylogenetic properties' are. Do the authors mean taxonomical profiles?"

We changed the sentence to clarify it: "These techniques are giving insight into taxonomic profiles and genomic components of microbial communities."

2. "Line 44. The authors mention 'high variability'. What is the feature showing this 'high variability'?"

High variability is referring to the diversity of organisms in one sample, uneven sequencing depth of the different organisms and other things that makes metagenomic research hard. We changed the word to use "their complexity".

3. "Line 52. Can the authors give examples of what they call 'computational resources specially for the metagenomics datasets'?"

We meant need for lot of memory and disk space, the use of cluster or cloud. They are not specific for metagenomic datasets, but probably highly required for metagenomics. We changed the

sentence to:
“They are command-line tools and may require extensive computational resources (memory, disk space)”.

4. “Line 140. What is a 'data reduction step'?”

A data reduction step is the reduction of the input data: removal of bad quality sequences and trimming, removal of duplicated sequences (dereplication), sorting of the sequences. We removed this term, to avoid confusion.

4. “This reviewer suggests removing the 'Installation and running section' and simply refers to the documentation, as done in other cases.”

We decided to have this section because it shows that using ASaiM is not really difficult and also to mention that tools and workflows can be added to any already existing Galaxy instance. We significantly shortened this section and referenced the documentation. Thanks for this recommendation.

Close
