# Supplementary material for: ASaiM: a Galaxy-based framework to analyze microbiota data
Source: Gigascience. 2018 May 15;7(6):giy057. doi: 10.1093/gigascience/giy057 (PMC6007547; doi:10.1093/gigascience/giy057)
Supplement: Response_to_Reviewer_Comments_Revision_1.pdf [file giy057_response_to_reviewer_comments_revision_1.pdf]

Author's Response To Reviewer Comments

Close

We thanked the reviewers for their new reviews. We tried to answer all of them, but we are not sure about the point raised by the Reviewer #2.

Reviewer #2

Please make sure to double check the Figures before publication. Figure 3 seems to have its title overlap with some other text.

We are sorry for the inconvenience, but can not see this locally. We will wait for the proofs and make sure this does not end up in the final PDF.

Reviewer #3

While this revised version of the manuscript improves on the previously submitted one, this Reviewer believes that a few points still need to be addressed:

1. While this Reviewer agrees that ASaiM allows users to overcome "the difficulty to find, configure, use and combine the dedicated bioinformatics tools", it is still true that "to extract useful information, a sequenced microbiota sample has to be processed by sophisticated workflows with numerous successive bioinformatics steps", that "Each step may require execution of several tools or software", that "[tools] may require extensive computational resources (memory, disk space)", and, finally, that "selecting the best tools, configuring them to use the correct parameters and appropriate computational resources and combining them together in an analysis chain is a complex and error-prone process.". This Reviewer suggests reframing the manuscript either stressing on ASaiM's strengths compared to state-of-the-art tools (that is, in this Reviewer's opinion, saving the users from the hassle of installing all the pieces of software, and implementing a few well-known pipelines
into Galaxy, an universally-acknowledged user-friendly platform), or clarifying, how ASaiM solves the issues raised above (that is, mostly, how i) ASaiM diminishes the memory/space requirements, ii) helps users in designing novel meaningful pipelines using the >100 tools included, and iii) helps users in setting meaningful parameters/resources in each of these steps). Following on this comment, the limitation of both QIIME and Mothur that is: "Designed for amplicon data, both QIIME and Mothur can not be directly applied to shotgun metagenomics data." is still not addressed by their ASaiM implementation and should, in this Reviewer's opinion, be removed.

The authors understand the first point of the reviewer and tried to clarify in the manuscript how ASaiM solves the raised issues. In the introduction of the workflow section, the authors add sentences to show how ASaiM helps users in setting meaningful parameters for tools and also in designing novel meaningful workflows. In the conclusion, the authors added few words to insist on the automated hassle of tool installation. Galaxy via ASaiM will not address the memory limitations, but Galaxy will efficiently schedule jobs as well as manage the memory usage. This information has also been added in the manuscript.
For Mothur and QIIME related question, ASaiM offered tools and workflows for amplicon or metataxomic data using QIIME and Mothur, but also for shotgun metagenomics data (using MetaPhlAn2 and HUMAnN2). ASaiM is not only then focused on amplicon data as QIIME and Mothur

are.

2. In this Reviewer's opinion, the comparison between ASaiM and the EBI pipeline is irrelevant, since they use different tools (and it rather seems a comparison between these tools). If the authors cannot provide a fair comparison, this paragraph could, in this Reviewer's opinion, be removed without loss of information.

The idea of the comparison between ASaiM and EBI metagenomics was to demonstrate the limitation of one approach against the other on the analysis of shotgun metagenomic data. We are not benchmarking the tools, just trying to illustrate the possibilities of ASaiM.

3. This Reviewer agrees that time and other computational requirements greatly depend on the input data, and thus suggests carrying on a benchmarking of all the implemented pipelines using multiple datasets, with different numbers of reads (many, as those belonging to the Hunan Metagenome Project, are freely available). This will help users in "selecting the best tools, configuring them to use the correct parameters and appropriate computational resources", and give them more useful information than that which can be extracted by only two datasets.

Such general benchmarking would be interesting and we are working currently together with other researchers to establish a general benchmarking, as mentioned by the reviewer. Using the information of the benchmarking, we would like to build an environment where users could be helped with tool selection and configuration and jobs/workflows automatically tweaks in Galaxy. We feel that this is out of scope for the manuscript but we are working on this as a more general framework, probably not only for metagenomics.

4. Minor comment: since there is no agreement yet on some of the terms used, it may be worth using 16S rRNA marker gene sequencing or amplicon sequencing, instead of metataxonomic, and whole metagenomic shotgun sequencing, instead of simply metagenomics.

The authors agree that there is a confusion in vocabulary used in the field of microbial community analysis. Marchesi & Ravel in their 2015 paper (Microbiome: (https://microbiomejournal.biomedcentral.com/articles/10.1186/s40168-015-0094-5) tried to establish a consensus vocabulary. To support this initiative, the authors decided to use the terms and definitions given in this paper. We hope this makes our paper more readable in the long run and supports the initiative started by Marchesi & Ravel.

Close
