# Supplementary material for: ASaiM: a Galaxy-based framework to analyze microbiota data
Source: Gigascience. 2018 May 15;7(6):giy057. doi: 10.1093/gigascience/giy057 (PMC6007547; doi:10.1093/gigascience/giy057)
Supplement: Reviewer_1_Report_(Original_Submission) -- Konstantinos Krampis, PhD — 9/13/2017 Reviewed [file giy057_reviewer_1_report_(original_submission).pdf]

Reviewer Report

Title: **ASaiM: a Galaxy-based framework to analyze microbiota data**

Version: **Original Submission** **Date:** 9/13/2017

Reviewer name: **Konstantinos Krampis, PhD**

Reviewer Comments to Author:

Excellent paper, useful collections of tools, focused approach and well organized with great documentation. Enough background for a software paper, my suggestion would be if you can mention a little more on metagenomics pipelines available on the main Galaxy server, in addition to an example of specialized Galaxy servers for metagenomics - for example the Metaphlan group they have such a specialized server: https://huttenhower.sph.harvard.edu/galaxy/In addition I was really excited to see the provenance mentioned. Since the documentation is so extensive (and excellent!), perhaps the authors could add a section on how to save a docker container where data has been processed with their tool (also how to bundle the volumes with the data), so that the whole package and be distributed (and provide analysis provenance), to collaborators, with a publication etc.Overall an excellent paper !

Level of Interest

Please indicate how interesting you found the manuscript: An exceptional article

Quality of Written English

Please indicate the quality of language in the manuscript: Acceptable

Declaration of Competing Interests

Please complete a declaration of competing interests, considering the following questions:

- Have you in the past five years received reimbursements, fees, funding, or salary from an organisation that may in any way gain or lose financially from the publication of this manuscript, either now or in the future?
- Do you hold any stocks or shares in an organisation that may in any way gain or lose financially from the publication of this manuscript, either now or in the future?
- Do you hold or are you currently applying for any patents relating to the content of the manuscript?
- Have you received reimbursements, fees, funding, or salary from an organization that holds or has applied for patents relating to the content of the manuscript?
- Do you have any other financial competing interests?
- Do you have any non-financial competing interests in relation to this paper?

If you can answer no to all of the above, write 'I declare that I have no competing interests' below. If your reply is yes to any, please give details below.

No competing interests.

I agree to the open peer review policy of the journal. I understand that my name will be included on my report to the authors and, if the manuscript is accepted for publication, my named report including any attachments I upload will be posted on the website along with the authors' responses. I agree for my report to be made available under an Open Access Creative Commons CC-BY license (http://creativecommons.org/licenses/by/4.0/). I understand that any comments which I do not wish to be included in my named report can be included as confidential comments to the editors, which will not be published.

I agree to the open peer review policy of the journal

To further support our reviewers, we have joined with Publons, where you can gain additional credit to further highlight your hard work (see: https://publons.com/journal/530/gigascience). On publication of this paper, your review will be automatically added to Publons, you can then choose whether or not to claim your Publons credit. I understand this statement.

Yes
