# Supplementary material for: ASaiM: a Galaxy-based framework to analyze microbiota data
Source: Gigascience. 2018 May 15;7(6):giy057. doi: 10.1093/gigascience/giy057 (PMC6007547; doi:10.1093/gigascience/giy057)
Supplement: Reviewer_2_Report_(Original_Submission) -- Efthymios Ladoukakis, Ph.D — 10/4/2017 Reviewed [file giy057_reviewer_2_report_(original_submission).pdf]

Reviewer Report

Title: ASaiM: a Galaxy-based framework to analyze microbiota data

Version: Original Submission **Date:** 10/4/2017

Reviewer name: Efthymios Ladoukakis, Ph.D

Reviewer Comments to Author:

Some spelling errors:line 56: blocking scientist -> blocking scientistsline 124 an web-interface -> a web-interfaceline 135: visualization -> visualizationsline 135: such Phyloviz -> such as Phylovizline 157 Figure 2): we -> Figure2) and we line 175-176: We integrate then also a workflow -> We also integrated them in a workflowin report (supp. material) targeted abundances may be not reflect -> targeted abundances may not reflect--Further remarks:1)The title is a bit lacking in context. ASAIM is clearly dedicated only towards the taxonomic and functional analysis of metagenomic data (either from amplicon sequencing or from shotgun sequencing). It would be beneficial for the reader to deduce that from the title.2)It's not quite clear the innovative part of the platform. Besides collecting all those preexisting tools in an organized manner under Galaxy's umbrella what was the added contribution of ASAIM's team? Did you develop new wrapper/parser scripts for some/all of these tools in order to integrate them with Galaxy? What is the added value of the 3 new tools you developped? The GO slim term tool seems to be one of the final tools (purple) in your workflow (is that correct?). What about the other two for searching EBI and ENA databases? Are they part of one of the workflows or just additional standalone tools?3)The comparison between ASAIM and EBI analysis seems rather trivial. It's not a comparison of the two platforms rather than a comparison of the two different tools they are using (QIIME and Metaphlan). It would make much more sense a comparison between EBI's workflow run in the exact same way as an ASAIM/Galaxy workflow with the same tools. 4)The same goes for functional analysis (where you mention comparison is not feasible). You just present results derived from two different methods with no comparable points.5)In line 200 the command you state docker run -d -p 8080:80 quay.io/bebatut/asaimis different than the one stated in your webpage where the installation instructions are:docker run -d -p 8080:80 quay.io/bebatut/asaim-frameworkwhile the "asaim" command doesn't work (not authorized error) the "asaim-framework" seems to work6)In supplementary material report page 3 contains a table that is not well displayed7)Installation was not succesful so actual testing of the tool was not possible. Installation in a new CentOS distribution (3.10.0-514) under a Virtuabox engine failed. It could be useful to mention in your docs how to install and start the docker engine before attempting to download the ASAIM package especially for those with little or no command line knowledge. At some point during the installation process there was an error saying:"failed to register layer: ApplyLayer exit status 1 stdout: stderr: write /tool_deps/_conda/envs/__picrust@1.1.1/lib/python2.7/site-packages/mpi4py/MPI.so: no space left on device."Not sure how that's possible with 34GB available free space. Does ASAIM include databases that take up more space than that? If that's the case you should probably include that in the Requirements section in your webpage and inform the reviewers as well in order for us to be able to succesfully install and properly test it.

Level of Interest

Please indicate how interesting you found the manuscript: An article whose findings are important to those with closely related research interests

Quality of Written English

Please indicate the quality of language in the manuscript: Needs some language corrections before being published

Declaration of Competing Interests

Please complete a declaration of competing interests, considering the following questions:

- Have you in the past five years received reimbursements, fees, funding, or salary from an organisation that may in any way gain or lose financially from the publication of this manuscript, either now or in the future?
- Do you hold any stocks or shares in an organisation that may in any way gain or lose financially from the publication of this manuscript, either now or in the future?
- Do you hold or are you currently applying for any patents relating to the content of the manuscript?
- Have you received reimbursements, fees, funding, or salary from an organization that holds or has applied for patents relating to the content of the manuscript?
- Do you have any other financial competing interests?
- Do you have any non-financial competing interests in relation to this paper?

If you can answer no to all of the above, write 'I declare that I have no competing interests' below. If your reply is yes to any, please give details below.

I declare that I have no competing interests

I agree to the open peer review policy of the journal. I understand that my name will be included on my report to the authors and, if the manuscript is accepted for publication, my named report including any attachments I upload will be posted on the website along with the authors' responses. I agree for my report to be made available under an Open Access Creative Commons CC-BY license (http://creativecommons.org/licenses/by/4.0/). I understand that any comments which I do not wish to be included in my named report can be included as confidential comments to the editors, which will not be published.

I agree to the open peer review policy of the journal

To further support our reviewers, we have joined with Publons, where you can gain additional credit to further highlight your hard work (see: https://publons.com/journal/530/gigascience). On publication of this paper, your review will be automatically added to Publons, you can then choose whether or not to claim your Publons credit. I understand this statement.

Yes
