# Supplementary material for: ASaiM: a Galaxy-based framework to analyze microbiota data
Source: Gigascience. 2018 May 15;7(6):giy057. doi: 10.1093/gigascience/giy057 (PMC6007547; doi:10.1093/gigascience/giy057)
Supplement: Reviewer_3_Report_(Original_Submission) -- Alessia Visconti — 10/9/2017 Reviewed [file giy057_reviewer_3_report_(original_submission).pdf]

Reviewer Report

Title: ASaiM: a Galaxy-based framework to analyze microbiota data

Version: Original Submission **Date:** 10/9/2017

Reviewer name: Alessia Visconti

Reviewer Comments to Author:

The manuscript describes an alternative workflow for the processing of shotgun metagenomics and metatranscriptomic data, called ASaiM.ASaiM integrates multiple tools for the analysis and manipulation of raw metagenomics and metatranscriptomic data, that are available, both as single tools and combined in multiple pipelines, within the Galaxy workflow and with a Docker and conda support. ASaiM comes with a very impressive documentation and it is of high importance in the metagenomics community, where most of the analyses are carried out using in-house scripts that, as pointed out by the authors, hinder reproducibility. However, several other metagenomics pipelines are already available: MG-RAST and the EBI metagenomics pipeline, that the authors briefly discuss in the Introduction, but also MOCAT2, MetAMOS, and another Galaxy metagenomic pipeline. How does ASaiM compare within this wider ecosystem? MOCAT2, for instance, comes with a set of preset parameters, stored in a single file, that already improve reproducibility, and the EBI metagenomics pipeline clearly shows the software version (e.g., https://www.ebi.ac.uk/metagenomics/pipelines/3.0), allowing provenance. Also, the authors point out that the main problems in analysing metagenomics data are, first, the selection and configuration of the necessary tools, then the definition of the correct computational resources, and, finally, the definition of a correct analysis workflow. However, in this reviewer's opinion, ASaiM does not fully address these limitations. The authors implement about 25 tools for the processing of metagenomics data but give little explanation on the reasons these specific tools have been selected, or which tools should be used when multiple tools within the same class are available. Novices in the field would surely appreciate these pieces of information as a way to select the correct software for the problem at hand. Regarding the workflows included in ASaiM, one is a reimplementation of the EBI workflow, one cannot be used for analysing metagenomic shotgun data, and only one is novel (that this reviewer supposes is the one called very generally "ASaiM"). This reviewer would suggest the authors to focus more on describing this novel workflow, and to remove all the references to QIIME and Mothur tools (or to 16S data analysis in general) since these are not able to analyse shotgun metagenomics data and may generate confusion. For instance, it would be interesting to know how the workflow can be customised, whether default parameters are available and how they have been selected, and have more detailed and exhaustive information on time and computational requirements (and not only on two samples).Also, it is not clear what improvements are brought by ASaiM and what are due to the usage of Galaxy (reproducibility, provenance, being user-friendly), or of HUMAnN2 (ability to infer the taxonomic profiles up to the species level, availability of genes and pathways abundances tables). For instance, how the proposed 'functional and taxonomic combination analysis' block differs with that proposed within the HUMAnN2 pipeline? More in general, this reviewer's main concern regards the focus of the manuscript. Are the authors interested in presenting the Galaxy implementation of a variety of metagenomics tools? Or to present a novel reproducible pipeline for the analysis of metagenomics data? Are they interested in metagenomics or metagenetics (16S) analysis? In this reviewer's opinion, the manuscript would surely benefit in focusing on a single message, while additional features (such as the analysis of metagenetics data) should be only briefly mentioned. The manuscript includes some imprecision, with several concepts repeated multiple times, and would surely benefit from a proofreading by a native speaker:1. Lines 40-43. Metagenomics and metatranscriptomics techniques do not allow to get insight into metabolic components, but only on the inferred functions of the micro-organisms present in one sample (as done, for instance, by HUMAnN2). To measure the metabolic components, one should use another approach, namely metametabolomics. It is also not clear what 'phylogenetic properties' are. Do the authors mean taxonomical profiles?2. Line 44. The authors mention 'high variability'. What is the feature showing this 'high variability'?3. Line 52. Can the authors give examples of what they call

'computational resources specially for the metagenomics datasets'?4. Line 140. What is a 'data reduction step'?4. This reviewer suggests removing the 'Installation and running section' and simply refers to the documentation, as done in other cases.

Level of Interest

Please indicate how interesting you found the manuscript: An article whose findings are important to those with closely related research interests

Quality of Written English

Please indicate the quality of language in the manuscript: Needs some language corrections before being published

Declaration of Competing Interests

Please complete a declaration of competing interests, considering the following questions:

- Have you in the past five years received reimbursements, fees, funding, or salary from an organisation that may in any way gain or lose financially from the publication of this manuscript, either now or in the future?
- Do you hold any stocks or shares in an organisation that may in any way gain or lose financially from the publication of this manuscript, either now or in the future?
- Do you hold or are you currently applying for any patents relating to the content of the manuscript?
- Have you received reimbursements, fees, funding, or salary from an organization that holds or has applied for patents relating to the content of the manuscript?
- Do you have any other financial competing interests?
- Do you have any non-financial competing interests in relation to this paper?

If you can answer no to all of the above, write 'I declare that I have no competing interests' below. If your reply is yes to any, please give details below.

None

I agree to the open peer review policy of the journal. I understand that my name will be included on my report to the authors and, if the manuscript is accepted for publication, my named report including any attachments I upload will be posted on the website along with the authors' responses. I agree for my report to be made available under an Open Access Creative Commons CC-BY license

(http://creativecommons.org/licenses/by/4.0/). I understand that any comments which I do not wish to be included in my named report can be included as confidential comments to the editors, which will not be published.

I agree to the open peer review policy of the journal

To further support our reviewers, we have joined with Publons, where you can gain additional credit to further highlight your hard work (see: https://publons.com/journal/530/gigascience). On publication of this paper, your review will be automatically added to Publons, you can then choose whether or not to claim your Publons credit. I understand this statement.

Yes
