# Supplementary material for: ASaiM: a Galaxy-based framework to analyze microbiota data
Source: Gigascience. 2018 May 15;7(6):giy057. doi: 10.1093/gigascience/giy057 (PMC6007547; doi:10.1093/gigascience/giy057)
Supplement: Reviewer_3_Report_(Revision_1) -- Alessia Visconti — 1/29/2018 Reviewed [file giy057_reviewer_3_report_(revision_1).pdf]

Reviewer Report

Title: ASaiM: a Galaxy-based framework to analyze microbiota data

Version: Revision 1 Date: 1/29/2018

Reviewer name: Alessia Visconti

Reviewer Comments to Author:

While this revised version of the manuscript improves on the previously submitted one, this Reviewer believes that a few points still need to be addressed:1. While this Reviewer agrees that ASaiM allows users to overcome "the difficulty to find, configure, use and combine the dedicated bioinformatics tools", it is still true that "to extract useful information, a sequenced microbiota sample has to be processed by sophisticated workflows with numerous successive bioinformatics steps", that "Each step may require execution of several tools or software", that "[tools] may require extensive computational resources (memory, disk space)", and, finally, that "selecting the best tools, configuring them to use the correct parameters and appropriate computational resources and combining them together in an analysis chain is a complex and error-prone process.". This Reviewer suggests reframing the manuscript either stressing on ASaiM's strengths compared to state-of-the-art tools (that is, in this Reviewer's opinion, saving the users from the hassle of installing all the pieces of software, and implementing a few well-known pipelines into Galaxy, an universally-acknowledged user-friendly platform), or clarifying, how ASaiM solves the issues raised above (that is, mostly, how i) ASaiM diminishes the memory/space requirements, ii) helps users in designing novel meaningful pipelines using the >100 tools included, and iii) helps users in setting meaningful parameters/resources in each of these steps). Following on this comment, the limitation of both QIIME and Mothur that is: "Designed for amplicon data, both QIIME and Mothur can not be directly applied to shotgun metagenomics data." is still not addressed by their ASaiM implementation and should, in this Reviewer's opinion, be removed.2. In this Reviewer's opinion, the comparison between ASaiM and the EBI pipeline is irrelevant, since they use different tools (and it rather seems a comparison between these tools). If the authors cannot provide a fair comparison, this paragraph could, in this Reviewer's opinion, be removed without loss of information.3. This Reviewer agrees that time and other computational requirements greatly depend on the input data, and thus suggests carrying on a benchmarking of all the implemented pipelines using multiple datasets, with different numbers of reads (many, as those belonging to the Hunan Metagenome Project, are freely available). This will help users in "selecting the best tools, configuring them to use the correct parameters and appropriate computational resources", and give them more useful information than that which can be extracted by only two datasets.4. Minor comment: since there is no agreement yet on some of the terms used, it may be worth using 16S rRNA marker gene sequencing or amplicon sequencing, instead of metataxonomic, and whole metagenomic shotgun sequencing, instead of simply metagenomics.

Level of Interest

Please indicate how interesting you found the manuscript: An article whose findings are important to those with closely related research interests

Quality of Written English

Please indicate the quality of language in the manuscript: Acceptable

Declaration of Competing Interests

Please complete a declaration of competing interests, considering the following questions:

- Have you in the past five years received reimbursements, fees, funding, or salary from an organisation that may in any way gain or lose financially from the publication of this manuscript, either now or in the future?
- Do you hold any stocks or shares in an organisation that may in any way gain or lose financially from the publication of this manuscript, either now or in the future?
- Do you hold or are you currently applying for any patents relating to the content of the manuscript?
- Have you received reimbursements, fees, funding, or salary from an organization that holds or has applied for patents relating to the content of the manuscript?
- Do you have any other financial competing interests?
- Do you have any non-financial competing interests in relation to this paper?

If you can answer no to all of the above, write 'I declare that I have no competing interests' below. If your reply is yes to any, please give details below.

I declare that I have no competing interests

I agree to the open peer review policy of the journal. I understand that my name will be included on my report to the authors and, if the manuscript is accepted for publication, my named report including any attachments I upload will be posted on the website along with the authors' responses. I agree for my report to be made available under an Open Access Creative Commons CC-BY license (http://creativecommons.org/licenses/by/4.0/). I understand that any comments which I do not wish to be included in my named report can be included as confidential comments to the editors, which will not be published.

I agree to the open peer review policy of the journal

To further support our reviewers, we have joined with Publons, where you can gain additional credit to further highlight your hard work (see: https://publons.com/journal/530/gigascience). On publication of this paper, your review will be automatically added to Publons, you can then choose whether or not to claim your Publons credit. I understand this statement.

Yes
